# Calcium signaling pathway implicates a shared genetic basis between psychiatric and cardiovascular diseases

**DOI:** 10.1038/s41398-026-04222-z

**Published:** 2026-07-11

**Authors:** Yunqing Zhu, Guorui Zhao, Yuyanan Zhang, Zhe Lu, Zhewei Kang, Xiaoyang Feng, Yundan Liao, Junyuan Sun, Rui Yuan, Yang Yang, Jing Guo, Bing Liu, Yaoyao Sun, Weihua Yue

**Affiliations:** 1 Peking University Sixth Hospital, Peking University Institute of Mental Health, NHC Key Laboratory of Mental Health (Peking University), National Clinical Research Center for Mental Disorders (Peking University Sixth Hospital), Beijing, 100191 China; 2 State Key Laboratory of Cognitive Neuroscience and Learning, Beijing Normal University, Beijing, 100875 China; 3 PKU-IDG/McGovern Institute for Brain Research, Peking University, Beijing, 100871 China; 4 Institute of Advanced Clinical Medicine, Peking University, Beijing, 100191 China

**Keywords:** Psychiatric disorders, Genetics

## Abstract

Psychiatric and cardiovascular diseases (CVDs) are frequently comorbid and are interconnected through the brain-heart axis. However, the underlying shared genetic etiology remains unknown in East Asians. To address this critical gap, we conducted a genome-wide pairwise trait pleiotropy study by leveraging genome-wide association studies of three major psychiatric disorders (schizophrenia [SCZ], bipolar disorder [BIP], major depressive disorder [MDD]) and ten cardiovascular traits (including eight CVDs) in East Asians. We identified genetic overlaps across seven disease pairs, such as SCZ with coronary artery disease. Through this pairwise approach, six of a total of 18 pleiotropic loci demonstrated tissue-specific expression in brain and cardiovascular systems. In the cross-ancestry replication, nine of the pleiotropic loci were validated. Among the novel pleiotropic genes, *TPCN1, CACNA2D2, CACNA1D*, and *ATP2B1* are involved in voltage-dependent calcium channel activity, regulation of calcium influx, enriched in calcium-related pathway. We validated association with calcium signal pathway in an independent cohort. Calcium pathway-specific polygenic risk score for SCZ was associated with prolonged corrected QT (QTc) interval, which remained robust among individuals free from QTc-affecting drugs. Given that calcium-channel blockers are commonly prescribed for heart and blood vessel conditions, we performed drug target analysis by integrating gene expression profiles from the brain and cardiovascular tissues. Our findings implicated that calcium-channel blockers and peripheral vasodilators elevated SCZ risk, diuretics reduced the risks of SCZ, BIP, and MDD. Our study reveals extensive shared genetic architectures underlying psychiatric and CVDs, which warrant prudence in the use of calcium channel blockers among patients with concurrent psychiatric and CVDs.

## Introduction

The burden of comorbidity between psychiatric disorders and cardiovascular diseases (CVDs) is substantial worldwide. A meta-analysis including 92 studies reported that the prevalence of CVD in patients with severe mental illness reached 9.9% [1]. In Asia, an 11-year cohort study of 0.2 million individuals in Taiwan indicated that patients with schizophrenia (SCZ) or bipolar disorder (BIP) had 41 and 51% higher risks of acute myocardial infarction (MI), respectively [2]. They also showed elevated risks of stroke [3, 4]. A prospective cohort study of 0.5 million Chinese reported that depression was associated with 33% higher risk of ischemic heart disease (IHD) [5]. Conversely, CVD also increased the risk of depression among Asians [6, 7].

The interplay between CVD and mental health, conceptualized as the brain-heart axis, has prompted ongoing investigation into its underlying mechanisms, including neural pathways such as communication between the central and autonomic nervous systems, mechanical signaling through mechanoreceptor-mediated hemodynamic sensing, and biochemical pathways involving the hypothalamic-pituitary-adrenal axis and inflammatory signals [8]. It also suggests the existence of common genetic determinants that confer susceptibility to both diseases [9]. Conventional genetic research had predominantly adopted a single-disease analysis, elucidating the etiology and therapeutic prospects of either psychiatric or cardiovascular conditions [10, 11]. There remains a critical knowledge gap regarding their shared pathological mechanisms when applying trans-disease approaches, particularly in East Asian cohorts. Investigating the underlying genetic basis of the brain-heart axis could facilitate the prevention and treatment targeting both psychiatric and cardiovascular diseases.

Shared genetic liability underlying the brain-heart axis has been characterized among Europeans. Overall, genetic overlap has been observed between schizophrenia (SCZ), bipolar disorder (BIP), major depressive disorder (MDD), and coronary artery disease (CAD) [12]. Hundreds of pleiotropic loci shared between major psychiatric disorders and CAD were identified. The pleiotropic loci were enriched in metabolic, neurodevelopmental, and other biological pathways [12–14]. However, such findings have not been explored in East Asian populations.

Single-disease genome-wide association studies (GWASs) implicated an obvious ancestry discrepancy of the genetic basis for psychiatric disorders and CVDs [15–17]. As European populations only host a proportion of human genetic variants, such bias costs our ability to discover shared genetic etiology that may be enriched in non-European populations. Evaluating the shared genetic components in East Asians based on large-scale GWASs was essential for understanding the genetic etiology and identifying those at high genetic risk for both diseases in the Asian population.

However, none of the previous studies have estimated the genetic overlaps and pleiotropic loci underlying the brain-heart axis among East Asian populations, nor did they include CVDs with serious inpatient burden in Asia (myocardial infarction [MI], ischemic stroke [IS], angina) [18]. Meanwhile, potential causal variants for both diseases were still unclear [19, 20]. Several biological pathways, such as the calcium-related pathway, were related to the etiologies of psychiatric disorders and CVDs in separate studies. Dysregulated calcium signaling may affect neuronal excitability and synaptic function [21], as well as cardiovascular excitation–contraction coupling and electrophysiological homeostasis [22]. However, whether these pathways contribute to shared genetic components has not been validated. The pathway-specific polygenic risk score (PRS), a mechanistically aware PRS investigating the shared genetic component mediated by a chosen pathway, could fill this gap. Furthermore, whether the shared genetic components drive causal effects of anti-CVD drug target genes on risks of psychiatric disorders has strong clinical relevance. The drug-target Mendelian randomization (MR) analysis evaluates such causal effects by applying genetic instruments for drug exposures associated with the expression levels of drug targets. To date, only the effects of antihypertensive drug target genes on psychiatric disorders have been estimated in Europeans [23], leaving the impacts of other anti-CVD drugs (e.g., peripheral vasodilators) unknown.

Based on the GWAS summary statistics of psychiatric disorders and CVDs among East Asians and Han Chinese individual-level data of the Chinese Antipsychotics Pharmacogenomics Consortium study (CAPOC), we aimed to: (1) estimate the shared genetic components between the two types of diseases; (2) identify novel pleiotropic loci, causal variants, and specifically expressed tissues, neuron types, enriched phenotypes and biological pathways; (3) estimate the causal effects of multiple classes of anti-CVD drugs on psychiatric disorders.

## Materials and methods

### Study design and GWAS dataset

**Fig. 1:**
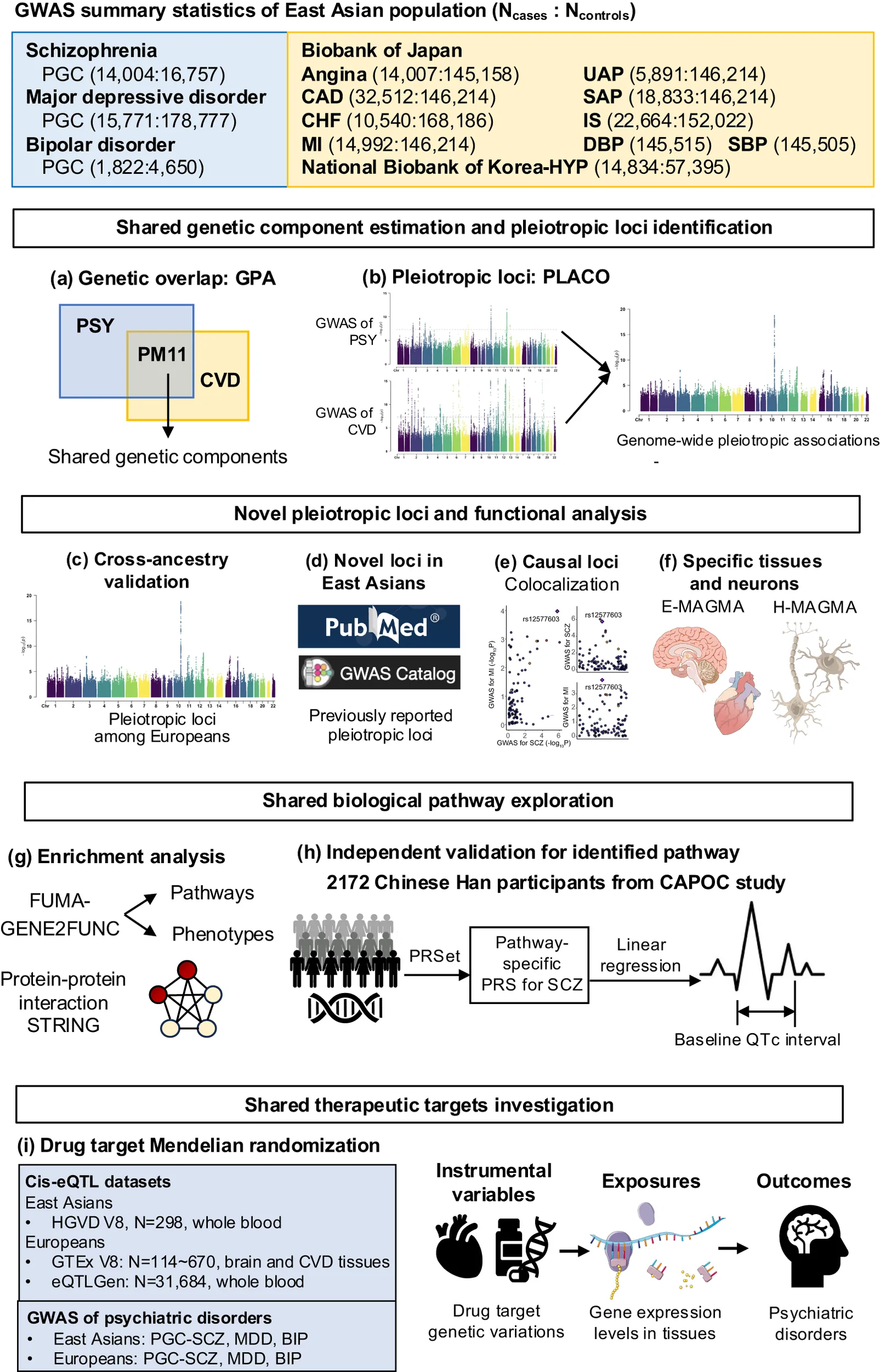
Study design. PGC Psychiatric Genomics Consortium, PSY psychiatric disorder, CVD cardiovascular disease, SCZ schizophrenia, BIP bipolar disorder, MDD major depressive disorder, CAD coronary artery disease, IS ischemic stroke, SBP systolic blood pressure, DBP diastolic blood pressure, HYP hypertension, MI myocardial infarction, SAP stable angina pectoris, UAP unstable angina pectoris, CHF chronic heart failure, GWAS genome-wide association study, GPA Genetic analysis incorporating Pleiotropy and Annotation, PM proportions of SNPs in the model, PLACO pleiotropic analysis under composite null hypothesis, MAGMA Multi-marker Analysis of GenoMic Annotation, eQTL expression quantitative trait loci, E-MAGMA eQTL-informed MAGMA, H-MAGMA Hi-C-coupled MAGMA, FUMA Functional Mapping and Annotation. PRS polygenic risk score, CAPOC Chinese Antipsychotics Pharmacogenomics Consortium. Genotype-Tissue Expression version 8 (GTEx V8) Human Genetic Variation Database (HGVD).

The overall study design is shown in Fig. 1. A genome-wide pairwise pleiotropy study was performed by leveraging GWAS summary statistics of three major psychiatric disorders and ten cardiovascular traits (including eight CVDs and two blood pressure phenotypes) in East Asians (Supplemental Table 1). The GWAS summary statistics of psychiatric disorders were obtained from the Psychiatric Genomics Consortium (PGC, 14,004 SCZ cases, 16,757 controls [24]; 15,771 MDD cases, 178,777 controls [16]) and GWAS of BIP among Han Chinese participants (1822 cases, 4650 controls) [25]. The GWAS summary statistics of cardiovascular diseases were obtained from the Biobank of Japan study [26, 27], including angina (14,007 cases, 145,158 controls), CAD (32,512 cases, 146,214 controls), chronic heart failure (10,540 cases, 168,186 controls), IS (22,664 cases, 152,022 controls), MI (14,992 cases, 146,214 controls), unstable angina pectoris (UAP, 5,891 cases, 146,214 controls), stable angina pectoris (SAP, 18,833 cases, 146,214 controls), systolic blood pressure (SBP, N = 145,505) and diastolic blood pressure (DBP, N = 145,515). GWAS for hypertension (HYP) was obtained from the National Biobank of Korea (14,834 cases, 57,395 controls) [28]. The GWAS populations across the two types of diseases were independent. A detailed description of the dataset is in Supplemental Methods and Supplemental Table 1.

All procedures contributing to the present study comply with the ethical standards of the relevant national and institutional committees on human experimentation and the Helsinki Declaration of 1975, as revised in 2008. All study protocols were approved by the institutional ethics review boards at each site. Written or verbal informed consent was obtained from all participants. Data analysis was performed from January 1^st^, 2024, to June 22^nd^, 2024. The present study followed the Strengthening the Reporting of Genetic Association Studies (STREGA) reporting guideline.

### Genetic overlap analysis

To assess the overall genetic overlap between three major psychiatric disorders and ten cardiovascular traits in East Asians, we applied the genetic analysis incorporating pleiotropy and annotation (GPA) analysis [29]. For each trait pair, GPA estimates the proportions of shared and trait-specific associated single-nucleotide polymorphisms (SNPs) and uses a likelihood ratio test to evaluate the overall genetic overlap. The pleiotropy association ratio (PAR) was calculated to evaluate the proportion of pleiotropic SNPs among SNPs associated with at least one trait. False discovery rate (FDR) was applied to correct for multiple tests (P_FDR_ < 0.05) [30] (Supplemental Methods).

### Genome-wide pleiotropic analysis

The psychiatric-cardiovascular disease pairs that showed shared genetic components in the GPA analyses were further included in the pleiotropic loci analysis. The pleiotropic analysis under the composite null hypothesis (PLACO) analysis [31] was applied for the pleiotropic loci identification on the genome-wide scale (P < 5 × 10^−8^). SNPs at linkage disequilibrium (LD) r^2^ > 0.1 or located within 250 kb were assigned to the same pleiotropic locus.

Pleiotropic genes were defined as the genes nearest to the pleiotropic loci (window = 10 kb) in PLINK 1.9. The Single Nucleotide Polymorphism Database (dbSNP) platform [32] was applied as a complementary approach. Functional annotation for the top SNPs was performed using ANNOVAR software [33]. The amino acid change information was obtained from RefSeq Gene and UCSC Known Gene. A Bayesian colocalization analysis was performed to pinpoint the possible causal SNP in each pleiotropic locus [19]. The posterior probability of H4 (PP.H4) larger than 0.6 was applied as the primary threshold to support causal SNPs [34, 35]. A sensitivity analysis using a more stringent threshold of PP.H4 > 0.8 was also performed [36] (Supplemental Methods).

### Cross-ancestry validation and identification of novel pleiotropic loci in East Asians

Cross-ancestry validation analysis was performed to replicate the pleiotropic loci identified in the present study. We performed genome-wide pleiotropic analysis between psychiatric and cardiovascular diseases among Europeans. For each pleiotropic locus identified in East Asians, the top SNP was considered replicated with the following criteria: (i) the top SNP was presented in the European dataset, if absent, at least one proxy SNP (LD r^2^ > 0.6) was required; (ii) the directions of T statistics for pleiotropic effects were consistent between East Asian and European populations; (iii) given known differences in genetic architecture across ancestries, we applied a nominal significance threshold of P < 0.05 to define replication. The locus-level validation was specifically referred to the corresponding disease pair in the European population.

Novel genes were defined as genes that did not overlap with the previously reported genes in pleiotropic studies among Europeans [12, 13, 37, 38] (searched by June 1^st^, 2024, on PubMed and GWAS catalog), and the corresponding loci were defined as the novel pleiotropic loci among East Asians. Considering the ancestral discrepancy, minor allele frequencies (MAFs) between the East Asians and Europeans were compared based on the allele frequencies reported in the 1000 Genomes Phase III-East Asian (1000 G EAS) [39]. Mann-Whitney U test was applied. The comparison was performed among the novel and known pleiotropic loci separately.

### Tissue and cell-type specificity, enrichment, and protein-protein interaction analyses

Based on the genome-wide pleiotropic loci identified in the PLACO analysis, expression quantitative trait loci (eQTL)-informed gene-level multimarker analysis of GenoMic annotation (E-MAGMA) [40] and Hi-C–coupled MAGMA (H-MAGMA) [41] analyses were performed to investigate tissue and cell-type specificity of the pleiotropic genes, respectively. Bonferroni correction for multiple testing of pleiotropic loci was applied. The gene-set enrichment analysis for the pleiotropic genes was performed using the functional mapping and annotation platform - GENE2FUNC function (https://fuma.ctglab.nl/gene2func) [42]. Gene Ontology (GO) and the GWAS catalog were selected as the gene sets. FDR was applied to multiple test corrections. The protein-protein interaction (PPI) analysis of the pleiotropic genes was performed using the STRING database (version 12.0, https://string-db.org/) [43]. The combined confidence score was used to define functional associations among proteins, and node degree was used to indicate the relative connectivity of each protein within the network.

### Pathway-Specific PRS analysis

The present enrichment analysis showed that shared loci between SCZ and CVD traits were overrepresented in the calcium pathway. Several studies separately reported calcium pathways related to the genetic basis of CVDs [22, 44] and SCZ [21, 45]. To emphasize the role of the calcium pathway, we estimated the associations between the calcium-specific pathway PRSs for SCZ with corrected QT (QTc) interval in the Han Chinese participants diagnosed with SCZ from the CAPOC study. The QT interval prolongation was a precise indicator of cardiac event [46].

The CAPOC study was conducted in 2010 based on five research centers in China [47]. A total of 3030 Han Chinese participants with diagnosed SCZ were enrolled at baseline (ChiCTR-TRC-10000934). The present study additionally excluded participants without qualified genotype data (N = 474), and without complete baseline information for the current analysis (N = 384), leaving 2172 participants for the PRS analysis.

We constructed a whole-genome PRS of SCZ using the PRS-CS [48]. PRSet was applied to calculate the gene-set-based PRS of SCZ for calcium-specific genetic pathways [49]. The genetic pathways related to calcium were selected from the GO in the MSigDB [50]. A P-value threshold of 1.00 was used for PRSet, considering a small proportion of SNPs in each gene set. Linear regression was used to estimate the association between whole-genome PRS of SCZ, and each of the calcium pathway-specific PRSs on the QTc interval at baseline. To account for potential medication effects on the QT interval, we performed a sensitivity analysis excluding participants previously prescribed anti-CVD drugs (N = 3), or antipsychotics with moderate to high effects on the QT interval (N = 89) [51], leaving 2080 participants for analysis. In addition, given that the clinical profiles of SCZ and CVD are likely to differ across age groups, we performed age-stratified analyses in a younger group (< 30 years, N = 1043) and an older group (≥ 30 years, N = 1129). Details are described in the Supplementary Methods and Supplemental Fig. 1.

### MR analysis for anti-CVD drug target gene expressions on psychiatric disorders

To explore the potential clinical relevance of the shared genetic basis between psychiatric disorders and CVDs, we performed a two-sample MR between the expression levels of anti-CVD drug target genes in the brain and cardiovascular tissues, with psychiatric disorders (SCZ, BIP, and MDD) in East Asians and Europeans.

Among the East Asians, eQTL data in the blood from the Human Genetic Variation Database (N = 298) were applied [52]. GWAS of psychiatric disorders were from the East Asian-ancestry datasets of PGC [16, 24] and BIP GWAS among Chinese [25]. In Europeans, brain and cardiovascular tissue eQTL data were obtained from Genotype-Tissue Expression version 8 (GTEx V8, N = 114–670) [53] and eQTLGen consortium (N = 31,684) [54]. Psychiatric disorders GWASs were obtained from the European-ancestry datasets of PGC [24, 55, 56] (Supplemental Table 1–2). The GWAS populations for exposures and outcomes were independent.

Six classes of common anti-CVD drug target genes (antiarrhythmics, diuretics, peripheral vasodilators, beta-blockers, calcium channel blockers, and agents acting on the renin-angiotensin system) were included (Supplemental Table 3). The Wald ratio method was applied, considering that only one genetic variant was applied for each drug target gene. The Bonferroni correction was applied for multiple tests of different drug target genes and tissues. A detailed description of eQTL and GWAS summary statistics, selection of the genetic instruments, and statistics methods applied for the MR analysis can be found in the Supplemental Methods.

## Results

### Genetic overlap

Based on the GWAS summary statistics of psychiatric disorders and CVDs in East Asians, we observed suggestively significant genetic overlaps across seven disease pairs: SCZ with angina, MI, SAP, CAD, HYP, and SBP, as well as BIP with SBP (P < 0.05). These pairs were included in the pleiotropic loci identification. Among them, four disease pairs showed significant genetic overlaps (P_FDR_ < 0.05): SCZ with MI, SAP, CAD, and SBP, with corresponding PARs of 0.014, 0.125, 0.075, and 0.148. In addition, the GPA analysis identified suggestive significant genetic overlaps for SCZ with angina, HYP, and BIP with SBP (P < 0.05). In contrast, MDD showed no significant genetic overlap with CVDs (Supplemental Table 4).

### Pleiotropic loci for psychiatric disorders and CVDs

**Fig. 2:**
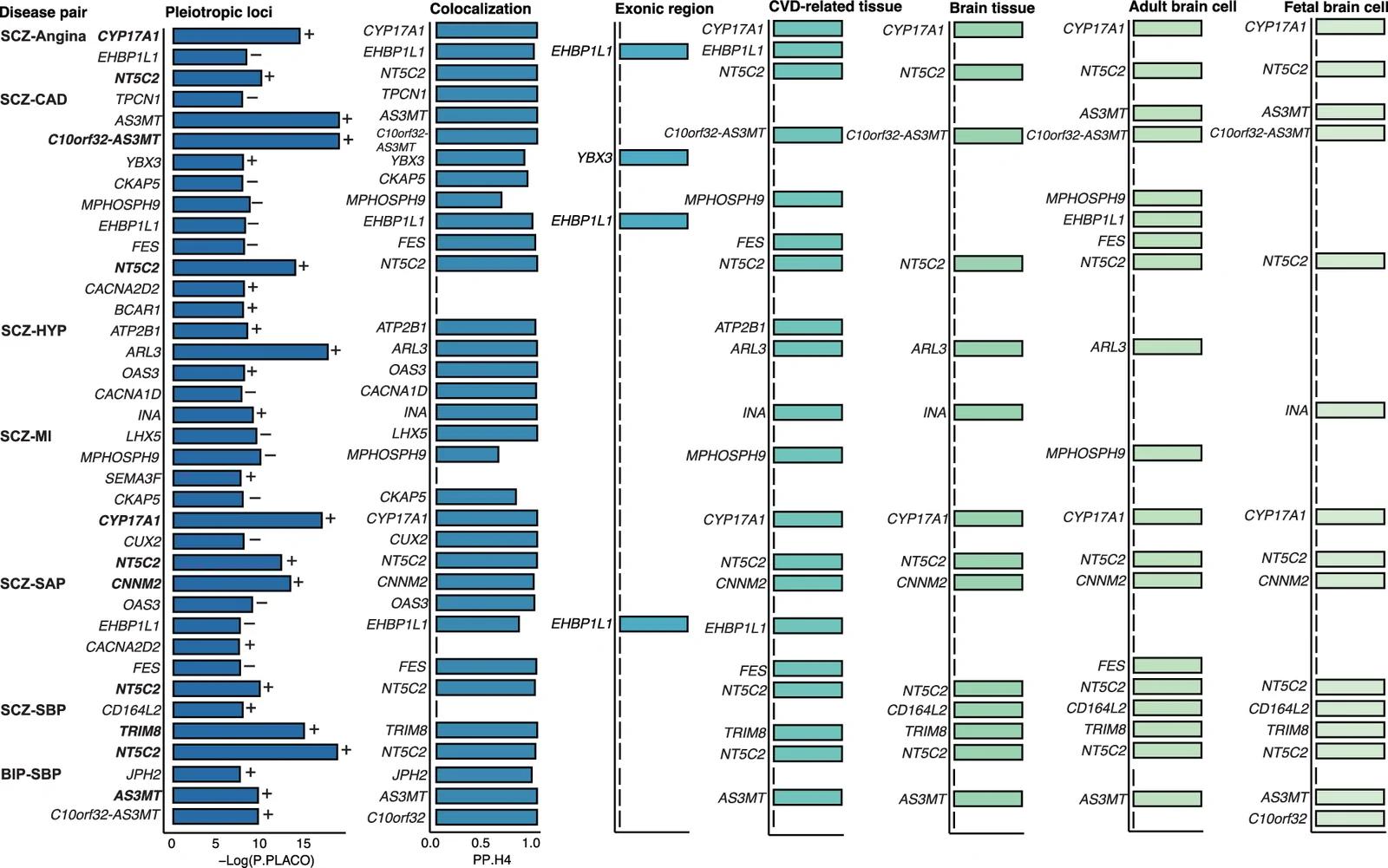
Evidence for the pleiotropic genes shared between psychiatric and cardiovascular diseases. BIP bipolar disorder, SCZ schizophrenia, CAD coronary artery disease, SBP systolic blood pressure, HYP hypertension, MI myocardial infarction, SAP stable angina pectoris, CVD cardiovascular disease, SNP single nucleotide polymorphism, MAGMA gene-level multimarker analysis of GenoMic annotation, E-MAGMA expression quantitative trait loci-informed-MAGMA, H-MAGMA Hi-C-coupled MAGMA. Seven lines of evidence for the candidate pleiotropic genes were shown in the figure, including the pleiotropic association of the top SNP for each pleiotropic locus, the posterior probability of H4 of the causal variants identified in the colocalization analysis, variants annotated in the exon regions, tissue-specific expression in brain and cardiovascular tissues (identified in E-MAGMA analysis), adult and fetal neurons (identified in H-MAGMA analysis). The nearest genes for pleiotropic loci were listed on the left. If top SNP had concordant effect directions on the two diseases (the effect allele increased the risks of both diseases), the pleiotropic locus was marked with a “+” in the line of pleiotropic association. Conversely, if the top SNP had discordant effect directions (effect allele increased the risk of one disease while decreasing the risk of another disease), the pleiotropic locus was marked with a “-”. The pleiotropic genes supported by pleiotropic association, colocalization, expression specificity in brain and cardiovascular tissues, adult and fetal brain cells, were indicated in bold font in the first line. The corresponding results for the expression specificity analysis can be found in Supplemental Tables 13–19.

A total of 18 pleiotropic loci were identified in the PLACO analysis across the seven disease pairs, including 3, 11, 6, 7, 6, 5, and 3 pleiotropic loci shared between SCZ and angina, CAD, HYP, MI, SAP and SBP, and between BIP and SBP, respectively (P.PLACO < 5 × 10^−8^, Fig. 2, Supplemental Table 5, Supplemental Figs. 2–3). The most significant association was observed at rs79680647 near *NT5C2* in the SCZ–SBP pair (P.PLACO = 2.50 × 10^−19^), followed by rs11191447 near *AS3MT, C10orf32-ASMT* in the SCZ–CAD pair (P.PLACO = 1.59 × 10^−19^). Among the top pleiotropic SNPs, 2, 5, 5, 3, 3, 5, and 3 SNPs showed concordant associations with each trait pair (T.PLACO statistics > 0, P.PLACO < 5 × 10^−8^), indicating that these SNPs simultaneously influenced the risks of both psychiatric and cardiovascular traits. The directions of these effects were consistent with those observed in the two corresponding single-disease GWASs (Supplemental Table 6). Several pleiotropic loci were identified across multiple pairwise diseases, such as the pleiotropic locus near *NT5C2* shared across five pairwise diseases, and the pleiotropic locus near *EHBP1L1* shared across three pairwise diseases.

### Cross-ancestry validation and novel pleiotropic loci among the East Asians

Among the 18 identified pleiotropic loci, nine were replicated in European populations. Notably, five of the 11 novel loci were validated in the replication analysis (all P values < 0.05). Details are shown in the Supplemental Table 7. Compared with previously reported pleiotropic loci in Europeans, we identified 11 novel pleiotropic loci and 13 novel pleiotropic genes that were first reported in East Asians. The novel genes included *C10orf32-ASMT, TRIM8, ATP2B1*, *CACNA2D2*, *CACNA1D*, *TPCN1*, et al. (Table 1). Among the novel loci, we observed significantly higher MAFs in East Asians than in Europeans (P = 0.007), while previously known loci didn’t show a significant difference in the MAFs between ancestries (P = 0.369) (Supplemental Fig. 4, Supplemental Table 8).

**Table 1:** Novel pleiotropic loci shared between psychiatric and cardiovascular diseases among East Asians.

Trait pair	Top SNP	CHR	BP	EA	OA	MAF in EAS	MAF in EUR	Nearest Gene	Pleiotropy analysis	
									T.PLACO	P.PLACO
SCZ-CAD	rs11066525	12	113713062	A	C	0.102	0.001	*TPCN1*	−18.639	1.61E-08
	rs11191447	10	104652323	C	T	0.287	0.090	*AS3MT, C10orf32-ASMT*	46.278	1.59E-19
	rs1126501	12	10875488	T	C	0.493	0.178	*YBX3*	18.892	1.27E-08
	rs116846611	11	46805571	A	T	0.040	0.001	*CKAP5*	−18.685	1.54E-08
	rs8180034	3	50518935	G	C	0.390	0.109	*CACNA2D2*	19.154	9.99E-09
SCZ-HYP	rs11105365	12	90070823	G	C	0.361	0.380	*ATP2B1*	19.702	4.25E-09
	rs17004846	4	81158221	A	G	0.455	0.349	*-*	18.771	1.01E-08
	rs312487	3	53545622	T	C	0.426	0.486	*CACNA1D*	−18.072	1.92E-08
	rs7076274	10	105046007	G	A	0.357	0.347	*INA*	21.308	9.62E-10
SCZ-MI	rs11066566	12	113891729	T	C	0.087	-	*LHX5*	−22.416	3.87E-10
	rs4688746	3	50224374	T	C	0.380	0.011	*SEMA3F*	17.954	2.40E-08
	rs56356278	11	46859787	T	A	0.097	0.148	*CKAP5*	−18.541	1.39E-08
SCZ-SAP	rs2072134	12	113409176	G	A	0.119	-	*OAS3*	−21.198	1.16E-09
	rs6788459	3	50520695	T	C	0.387	0.110	*CACNA2D2*	17.500	3.57E-08
SCZ-SBP	rs11105328	12	89942390	A	G	0.215	0.144	*-*	25.223	5.06E-11
	rs12028549	1	27713733	T	C	0.400	0.012	*CD164L2*	19.055	1.39E-08
	rs4146428	10	104401721	G	A	0.432	0.093	*TRIM8*	36.685	1.54E-15
	rs79317416	12	113921346	G	A	0.089	-	*-*	21.256	1.87E-09
BIP-SBP	rs76295717	6	43372476	G	A	0.024	-	*-*	19.916	4.26E-09

*BIP* bipolar disorder, *SCZ* schizophrenia, *CAD* coronary artery disease, *SBP* systolic blood pressure, *HYP* hypertension, *MI* myocardial infarction, *SAP* stable angina pectoris, *SNP* single nucleotide polymorphism, *CHR* chromosome, *BP* base pair position, *EA* effect allele, *OA* other allele, MAF in EAS, MAF in EUR, the minor allele frequencies of the corresponding top SNP in the 1000 Genomes Phase III-East Asians and Europeans; PLACO, pleiotropic analysis under the composite null hypothesis. The 11 novel pleiotropic loci, and 13 novel pleiotropic genes that were first reported among East Asians were shown in the table. *AS3MT* was the nearest gene to the rs11191447, while it was not a novel gene.

### Functional annotation, colocalization, and tissue and cell-type specificity

Functional annotation showed that rs3741380, located in the *EHBP1L1* gene, and rs1126501, located in the *YBX3* gene, were in the exonic regions with nonsynonymous effects (Supplemental Table 9). The colocalization analysis identified 15 of the 18 pleiotropic loci with PP.H4 > 0.6, among which 13 pleiotropic loci also met a more stringent threshold of PP.H4 > 0.8. In total, 23 shared causal SNPs were identified (Supplemental Table 10, Supplemental Fig. 5).

The E-MAGMA analysis showed that 83.3, 80.0, 48.0, 81.3, 31.6, 50.0, and 50.0% of the pleiotropic genes between BIP and SBP, and between SCZ and angina, CAD, HYP, MI, SAP, and SBP had tissue-specific expression in the brain and cardiovascular tissues (Supplemental Table 11–15). The H-MAGMA analysis indicated neuron-specific expression for more than half of the pleiotropic genes, with the proportion ranging from 50.0–100.0% (Supplemental Table 16–19, Supplemental Fig. 6–7). *AS3MT*, *CYP17A1*, *NT5C2*, *C10orf32*, *CNNM2*, and *TRIM8* were supported by colocalization, expression specificity in brain and cardiovascular tissues, and adult and fetal brain cell types (Fig. 2).

### Enrichment and PPI analyses

**Fig. 3:**
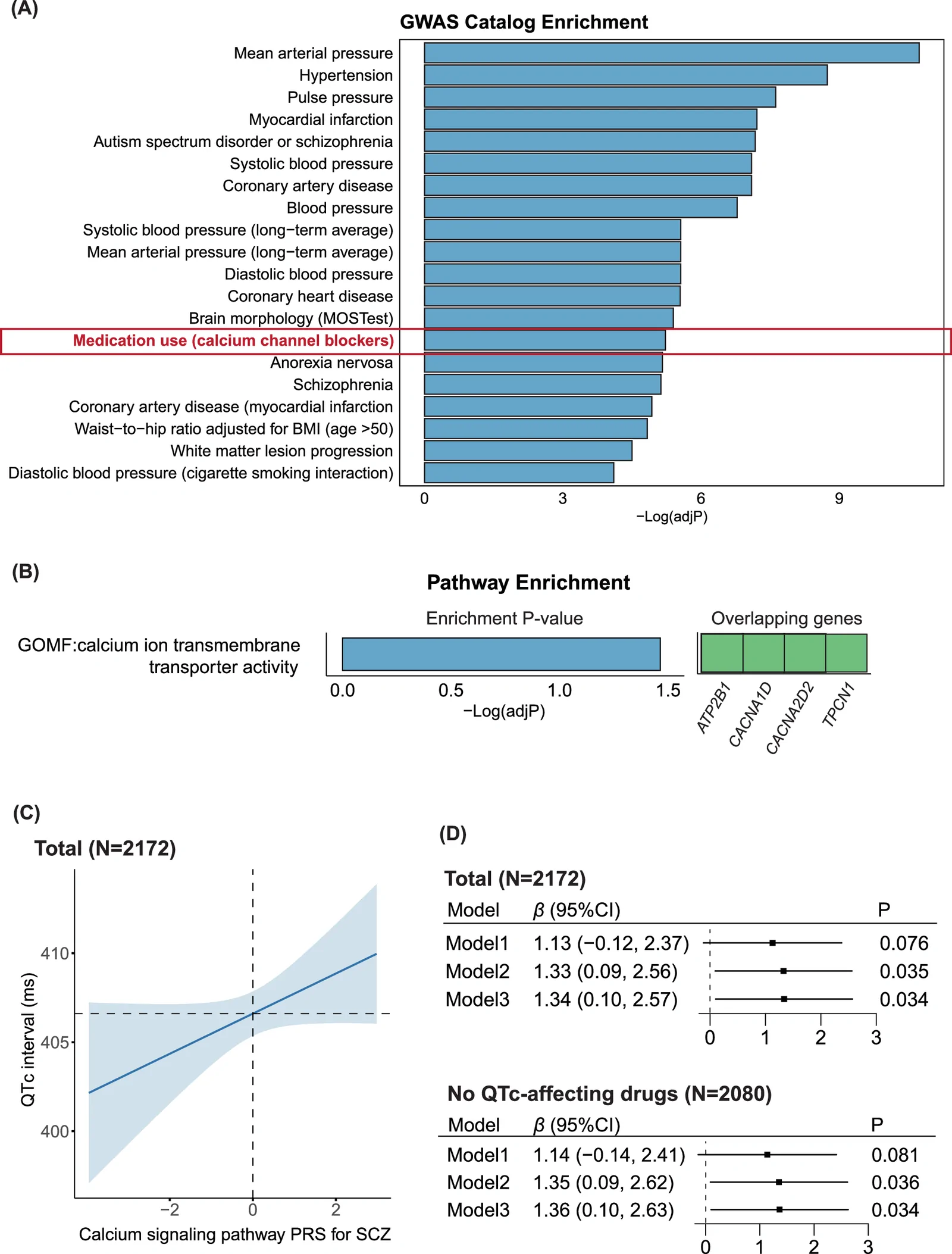
Enrichment and pathway-specific polygenic risk score (PRS) analyses. **A, B** displays the results of phenotype (**A**) and pathway enrichment analysis (**B**). Only the first 20 significant phenotypes and the significant biological pathway in the enrichment analysis were listed here. Please see Supplemental Table 20 for the results of enrichment analysis. **C, D** showed the associations between the calcium-specific pathway (GO:0007223) polygenic risk score (PRS) for schizophrenia (SCZ) on the corrected QT (QTc) interval among the Han Chinese individuals from the Chinese Antipsychotics Pharmacogenomics Consortium. In (**C**), the means of PRS and QTc interval were displayed in the dashed lines of the x and y axes, respectively. **D** displayed the associations in the full cohort (N = 2172, top) and participants not prescribed QTc-affecting medications (N = 2080, bottom). The forest plot showed beta estimates and 95% confidence intervals (CIs), Model 1 had no adjustment. Model 2 was adjusted for age, age^2^, sex, research centers, and the first five principal components. Model 3 was additionally adjusted for BMI, course of disease. The full result can be found in the Supplemental Tables 25–26.

The pleiotropic genes were overrepresented in the medication of calcium channel blockers (P_FDR_ = 5.91 × 10^−6^), cardiometabolic (e.g., adiposity), and neuropsychiatric (e.g., white matter lesion progression) phenotypes (P_FDR_ < 0.05) (Fig. 3A, Supplemental Table 20). *ATP2B1*, *CACNA1D*, *CACNA2D2*, and *TPCN1* were enriched in the pathway of calcium ion transmembrane transporter activity (GO: 0015085) (P_FDR_ = 0.034) (Fig. 3B, Supplemental Table 20).

PPI analysis identified two major clusters. In one cluster, *AS3MT* was the core node (node degree = 5), followed by *CNNM2, CYP17A1*, and *NT5C2* (node degree = 3). In the other cluster, *CACNA1D* served as the core node (node degree = 2), which connected *CACNA2D2* and *JPH2*. The strongest interactions were observed between *CACNA1D* and *CACNA2D2* (combined score = 0.971), and *AS3MT* and *CNNM2* (combined score = 0.818) (Supplemental Fig. 8, Supplemental Table 21–22).

### Pathway-specific PRS analyses

A total of 2172 participants of Han Chinese ancestry were included in the pathway-specific PRS analysis. The mean QTc interval was 406.6 ms (standard error = 0.64 ms), its distribution is shown in Supplemental Fig. 9. Other baseline characteristics are presented in Supplemental Table 23. No significant difference was observed between included and excluded samples (P > 0.05, Supplemental Table 24). We observed a positive association between the genome-wide SCZ PRS and the prolonged QTc interval (Model 3: *β* [95% confidence interval, 95% CI] = 1.36 [0.04, 2.67]). Three calcium-specific pathway PRSs were significantly associated with the risk of SCZ (self-contained and competitive P values < 0.05, Supplemental Table 25). Only one calcium signaling pathway-specific PRS (GO:0007223) of SCZ was positively associated with the level of QTc interval (Model 3: *β* [95% CI] = 1.34 [0.10, 2.57]) (Fig. 3C, D). The association was robust among those without prescribing drugs affecting the QTc interval (N = 2080) (Model 3: *β* [95% CI] = 1.36 [0.10, 2.63]) (Fig. 3D, Supplemental Table 26). This association was also observed in participants aged > 30 years (N = 1129) (Model 3: *β* [95% CI] = 1.93 [0.16, 3.70]) (Supplemental Table 26).

### Association between drug target gene expressions and risks of psychiatric disorders

**Fig. 4:**
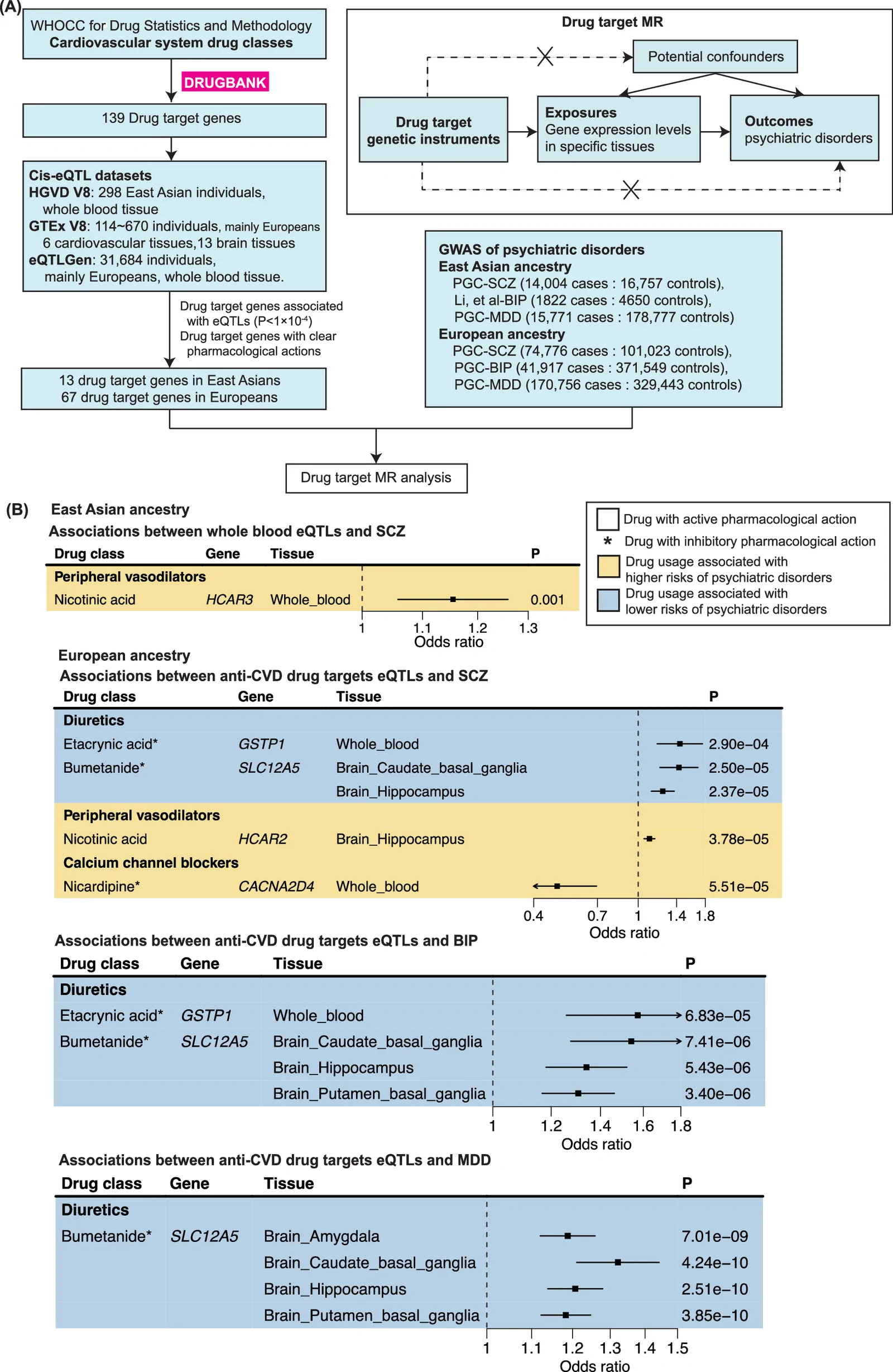
MR analysis between anti-CVD drug target expressions in brain and cardiovascular tissues with psychiatric disorders. Abbreviations: WHOCC World Health Organization Collaborating Centre, MR mendelian randomization, eQTL expression quantitative trait loci, HGVD Human Genetic Variation Database, GTE Genotype-Tissue Expression, SCZ schizophrenia, BIP bipolar disorder, MDD major depressive disorder, CVD cardiovascular disease. **A** displays the workflow of MR analysis. **B** showed the associations between expression levels of anti-CVD drug target genes and psychiatric disorders in East Asian and European populations. The target genes with active pharmacological actions by the corresponding drugs were unmarked, those with inhibitory pharmacological actions by the corresponding drugs were marked with “^*^”. MR results showed that anti-CVD drugs associated with a higher risk of psychiatric disorders were highlighted with a yellow background, while those associated with a lower risk were highlighted with a blue background. The corresponding results are in the Supplemental Table 28.

Among East Asians, increased *HCAR3* (an active target of nicotinic acid) expression in the blood was associated with a higher risk of SCZ (odds ratio [OR] [95% CI] = 1.15 [1.06, 1.26]). A similar association was observed among the Europeans, where the increased expression of *HCAR2* (an active target of nicotinic acid) in the brain was associated with SCZ (OR [95% CI] = 1.10 [1.05, 1.16]). As for diuretics, increased *GSTP1* expression in the blood and increased *SLC12A5* expression in the brain (both of which are inhibitory targets) elevated the risk of SCZ. The expression level of *CACNA2D4* in the whole blood (inhibitory target of nicardipine) was associated with a lower risk of SCZ in Europeans (OR [95% CI] = 0.49 [0.35, 0.69]). The expression levels of *SLC12A5* in brain tissues and *GSTP1* in the whole blood elevated the risk of BIP among Europeans. The expression levels of *SLC12A5* in brain tissues were also associated with a higher risk of MDD (Fig. 4, Supplemental Table 27–29). In addition, the elevated levels of SBP and DBP were not associated with the three psychiatric disorders (P > 0.05) (Supplemental Table 30–32).

## Discussion

In East Asians, we found genetic overlaps between SCZ, BIP, and diverse CVDs. We pinpointed 11 novel pleiotropic loci identified for the first time among Asians. Half of the pleiotropic loci were replicated in the cross-ancestry validation. The pleiotropic genes showed tissue-specific expression in the brain and cardiovascular tissues, and were overrepresented in the psychiatric and cardiometabolic phenotypes. Several novel loci were enriched in the calcium signaling-related pathway, suggesting shared genetic components between SCZ and QTc intervals in the Han Chinese population. The MR findings warrant great pharmacovigilance regarding the adverse effects of calcium channel blockers, peripheral vasodilators, and the potential benefits of diuretics for psychiatric disorders.

The present study estimated the shared genetic components between psychiatric and cardiovascular diseases in Asia from multiple aspects. The overall shared genomic components were estimated by genetic overlap analysis. To deeply probe into the shared genetic basis and underlying mechanism, we identified the pleiotropic loci, tissue- and cell-type specificity, and biological pathways. Drug target MR analysis brought clinical relevance to the shared genetic components. Beyond summary statistics-level analyses, we validated the role of the calcium signaling pathway through PRS analysis among Han Chinese individuals.

The genetic basis of complex diseases varies across different ancestries [10, 11]. Thus, we focused on the shared genetic components in East Asians and the differences compared with the findings from Europeans. Beyond the previous findings [12, 13, 37, 38], we applied the GWASs of diverse types of CVDs among East Asians and observed genetic overlaps between SCZ and MI, and SAP for the first time. In the replication among Europeans, nine of the 18 identified shared loci were successfully validated, underscoring the robustness of these pleiotropic associations. Further East Asian-based GWASs of psychiatric disorders with enriched patients are required to independently validate our findings within the same ancestral population.

Among the 13 novel pleiotropic genes identified in the Asian population, *C10orf32-AS3MT* and *TRIM8* showed convergent evidence in the association, colocalization, and expression levels, indicating that they were potential risk genes for both diseases. The *C10orf32-AS3MT* showed elevated expression in brain and heart tissues [57], upregulated during stem cell differentiation to neurons [58], and associated with SCZ and CVDs in East Asians [15, 26]. In vivo experiments åare warranted to explore the underlying mechanisms linking *C10orf32-AS3MT* to CVDs. *TRIM8* expression inhibited neuronal differentiation and excitatory synaptic transmission [59], contributing to the etiology of SCZ among Asians [60]; its overexpression in the heart exacerbated cardiac hypertrophy [61].

Notably, novel shared genes *CACNA1D, CACNA2D2, ATP2B1*, and *TPCN1* are key components of cellular calcium signaling pathways. *CACNA1D* and *CACNA2D2* encode subunits of the voltage-dependent calcium channel complex. *CACNA1D* mediates L-type calcium currents. Its dysregulation can impair cardiomyocyte Ca²⁺ handling, leading to electrical instability, arrhythmias, cardiac hypertrophy, and hypertension [62–64]. In the nervous system, L-type calcium channels shape neuronal excitability and circuit development, with *CACNA1D* alterations affecting hippocampal neurogenesis and dendritic growth [65]. *CACNA2D2*, an auxiliary alpha-2-delta subunit, modulates L-type and P/Q-type calcium channels, contributing to cardiac contractility and vascular tone [66]. It also regulates Ca²⁺-dependent neurotransmitter release and neuronal plasticity [67, 68]. In addition, *CACNA2D2* polymorphisms are associated with schizophrenia risk in Han Chinese women [69]. Furthermore, our present PPI analysis supported a direct functional interaction between *CACNA1D* and *CACNA2D2*, which showed the strongest connectivity in the network, reinforcing their coordinated roles within the voltage-dependent calcium channel complex. *ATP2B1* encodes PMCA1, a plasma membrane Ca²⁺ pump that regulates vascular tone by extruding cytosolic Ca²⁺, and has been consistently identified as a blood pressure susceptibility locus [70]. In neurons, PMCA1 also contributes to Ca²⁺ clearance and synaptic Ca²⁺ dynamics [71]. *TPCN1* encodes the endolysosomal Ca²⁺ channel two-pore channel 1 (TPC1) [72]. TPC1/2-dependent signaling promotes cardiac hypertrophy and remodeling [73] and is upregulated in failing human hearts [74]. In the central nervous system, TPC-mediated Ca²⁺ signaling contributes to synaptic plasticity and astrocytic autophagy [75], processes that are relevant to SCZ. Future clinical studies based on patients with psychiatric disorders are necessary to investigate the effects of polymorphisms in pleiotropic genes on CVDs.

The novel pleiotropic loci were enriched in the calcium signaling pathway, indicating a positive association between genetic predisposition to SCZ and the prolonged QTc interval in an independent Han Chinese cohort. These findings emphasized the calcium signaling pathway as a potential mechanism for the underlying shared genetic basis of the brain-heart axis in Asians. The dysfunction of voltage-gated calcium channels caused an influx of calcium ions, leading to irregular activity of cardiac muscle and vessel smooth muscle cells, influencing the progression of CVDs [76]. Meanwhile, the calcium ion signaling pathway was responsible for regulating neuronal gene transcription, neuronal excitability, functions of the prefrontal cortex and hippocampus, and impacted psychiatric disorders [21]. Furthermore, from the clinical aspect, our drug target MR analysis demonstrated that nicardipine (targeting *CACNA2D4*), a calcium channel blocker applied for angina and hypertension, was associated with an increased risk of SCZ. Previous studies indicated that both *CACNA2D2* and *CACNA1D* were associated with the pharmacological activities of antiarrhythmic and antihypertensive drugs [26, 77]. The findings reinforced the mechanistic link between calcium dysregulation and the co-occurrence of psychiatric and cardiovascular disorders, highlighting the cautious pharmacovigilance when prescribing calcium channel blockers to patients affected by both conditions.

Together with the calcium channel blockers, our drug target MR analysis identified a total of five target genes across three classes of anti-CVD drugs that were associated with risks of psychiatric disorders, suggesting potential therapy targets for both diseases. Through diverse mechanisms, different types of anti-CVD drugs showed inconsistent effects on psychiatric disorders. And their effects were independent of blood pressure [78]. Notably, the expression levels of active target genes (*HCAR2*, *HCAR3*) for nicotinic acid elevated the risk of SCZ, both in East Asians and in Europeans. Hydroxycarboxylic acid receptor 2 (HCAR2) and HCAR3 are involved in lipolysis and cellular immunity, the nicotinic acid has well-established antiatherogenic effects [79]. In addition, HCAR2 may exert neuroprotective effects by inhibiting NF-κB–mediated inflammatory activation in microglia and rescuing synaptic dysregulation in the inflamed central nervous system [80]. The *HCAR2* region-specific expression observed in the hippocampus may be biologically relevant, given its critical role in memory processing [81] and stress regulation [82], and has been closely linked to schizophrenia [83]. Together, the effects of *HCAR2* and *HCAR3* on lipid metabolism, cellular immunity, and neuroprotection contribute to SCZ pathogenesis. The clinical findings showed that nicotinic acid was associated with worsened psychotic symptoms [84]. Diuretics reduced risks of psychiatric disorders, in line with clinical studies reporting protective effects of bumetanide on neuropsychiatric symptoms [85–87]. The identified targets are involved in neuropsychiatric protection. *SLC12A5* was involved in potassium-chloride cotransport [88] and dendritic spine formation [89]. *GSTP1* was related to the prevention of neurodegeneration through regulation of cyclin-dependent kinase 5 activity [90]. Notably, the brain regions implicated in *SLC12A5* expression—including the caudate and putamen of the basal ganglia, the amygdala, and the hippocampus—are closely linked to psychiatric pathophysiology, given their respective roles in cortico-striatal circuitry [91], emotional regulation [92], and stress-related processing [82]. Further randomized controlled trials are indispensable to validate the causal effects of these anti-CVD drugs on aggravation or remission of psychiatric disorders to improve the current prescription guidelines. Future studies integrating neuroimaging with region-specific gene expression are warranted to clarify how altered expression of these drug targets in implicated brain regions influences the risk of psychiatric disorders.

To our knowledge, we performed the first genome-wide pleiotropic study that depicted a shared genetic basis for the brain-heart axis from multiple perspectives in Asians. Several limitations could not be avoided. First, even though eQTLs in the blood among East Asians were applied, the expression levels in the brain were derived from Europeans, which might bias the tissue-specific expression findings when leveraging the pleiotropic loci identified in Asians. Second, although cases in SCZ and BIP GWASs were clinically ascertained, GWAS data for clinically diagnosed MDD remain limited in East Asians, and relatively low heritability and case proportion in available datasets might reduce our power to detect shared genetic mechanisms related to MDD. In addition, the relatively small sample size of clinically diagnosed BIP GWAS might constrain our identification of BIP-related shared genetic signals. Last, drug-target genetic instruments represent lifelong exposures, which differ from short-term randomized pharmacologic trials, and real-world compounds might have off-target and side effects that require further clinical evaluation.

## Conclusions

The present study illustrated the shared genetic architecture of the brain-heart axis in East Asians based on large-scale GWASs of psychiatric disorders and CVDs. We identified 18 pleiotropic loci, among which 11 were novel. The pleiotropic loci showed tissue-specific expression in both the brain and cardiovascular systems, and were enriched in the calcium signaling pathway. Our findings firstly suggested calcium channel blocker therapy warrants clinical prudence in the management of patients with concurrent psychiatric and cardiovascular diseases, which may be further validated and highlighted in clinical guidance. The present study not only brought novel insights into genetic risk factors and precision clinical diagnosis, but also paved the way to tailored interventions and therapeutic targets for both disease groups in the Asian population.

## Supplementary information

Supplementary Methods and Supplementary Figure Legends

Supplementary Figures

Supplementary Tables

## Data Availability

The GWAS and expression quantitative trait loci (eQTL) summary statistics were publicly available. The GWAS summary statistics for psychiatric disorders in the Psychiatric Genomics Consortium could be obtained through the PGC website (https://pgc.unc.edu/for-researchers/download-results/). GWAS summary statistics for cardiovascular diseases (CVDs) from the Biobank of Japan and the National Biobank of Korea could be obtained from the corresponding websites (https://pheweb.jp/; https://koges.leelabsg.org/). The eQTL data from the Human Genetic Variation Database could be obtained from the website (https://www.hgvd.genome.med.kyoto-u.ac.jp/download.html). The eQTL data from the Genotype Tissue Expression (GTEx) and eQTLGen were obtained from the corresponding portals (https://gtexportal.org/home/downloads/; https://www.eqtlgen.org/). The GWAS summary statistics of bipolar disorder in Chinese were obtained by contacting the author (Prof. Ming Li, limingkiz@mail.kiz.ac.cn) of *Novel Risk Loci Associated With Genetic Risk for Bipolar Disorder Among Han Chinese Individuals: A Genome-Wide Association Study and Meta-analysis* (PMID: 33263727). Constrained by the local law on the management of human genetic resources and the requirements of the research project, the sharing of individual-level data of the Chinese Antipsychotics Pharmacogenomics Consortium study was restricted from public access. The data that support the findings of this study are available from the corresponding author, WY (dryue@bjmu.edu.cn), upon reasonable request with the proposal. All requests must be approved by the relevant ethics boards and data custodians.

## Code availability

The codes used for the data analyses in this study can be made available by contacting the corresponding author, WY (dryue@bjmu.edu.cn).
