# Supplementary material for: Calcium signaling pathway implicates a shared genetic basis between psychiatric and cardiovascular diseases
Source: Transl Psychiatry. 2026 Jul 11;16:527. doi: 10.1038/s41398-026-04222-z (PMC13642958; doi:10.1038/s41398-026-04222-z)
Supplement: Supplementary file 1 [file 41398_2026_4222_MOESM2_ESM.pdf]

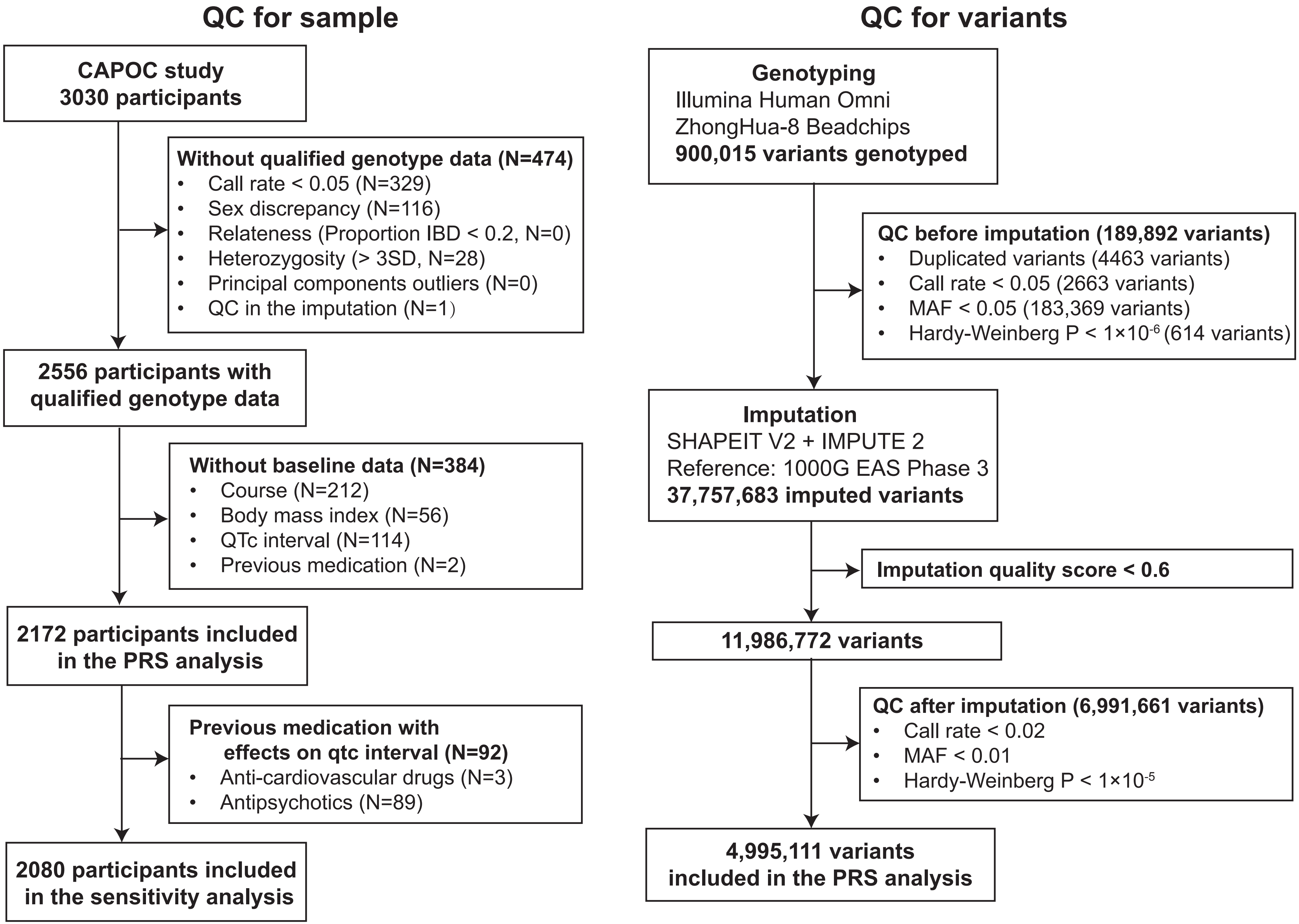

Supplemental Figure 1. Quality control for the sample and variants in the Chinese Antipsychotics Pharmacogenomics Consortium study

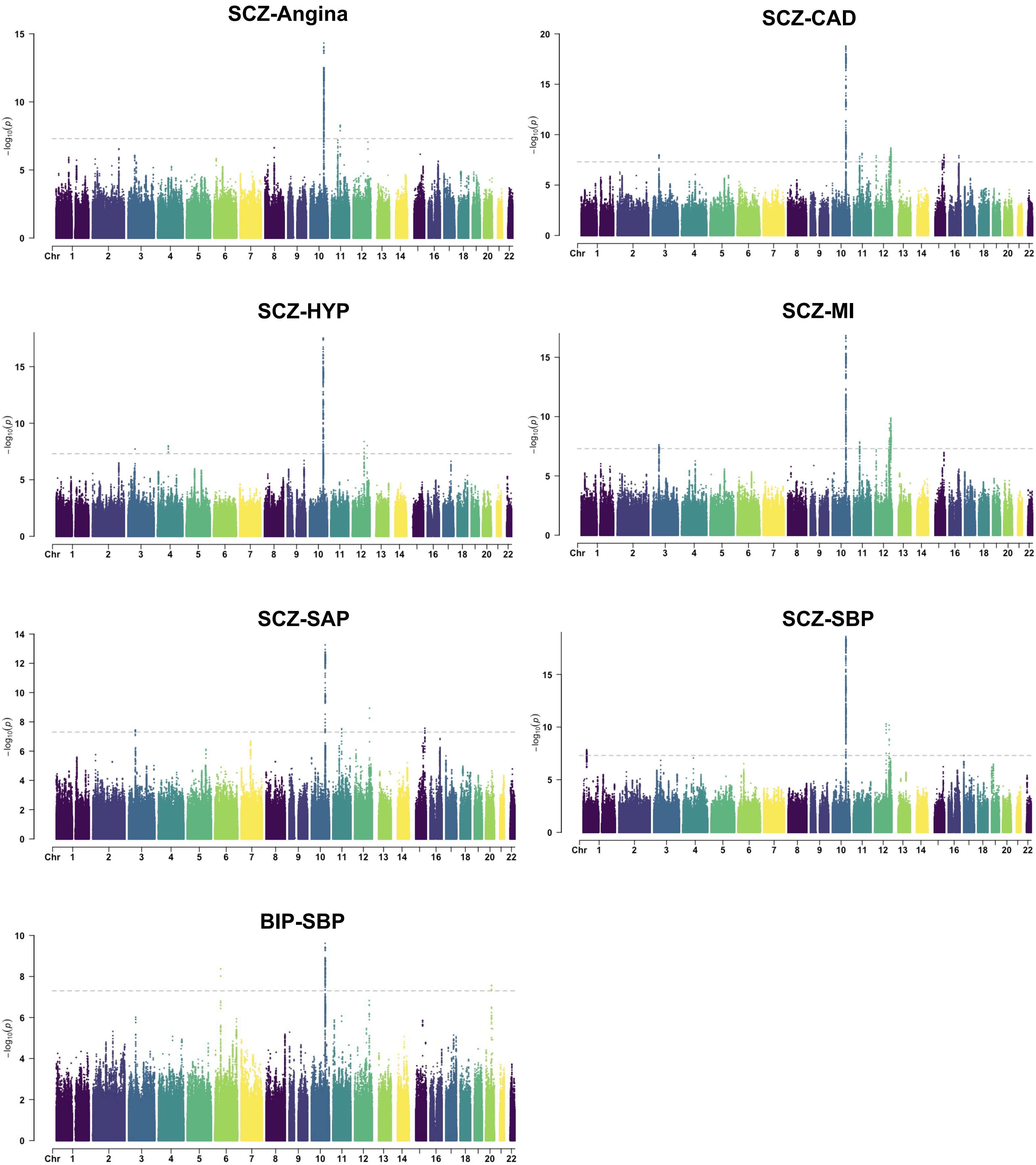

Supplemental Figure 2. Manhattan plots for the genome-wide pleiotropic analysis

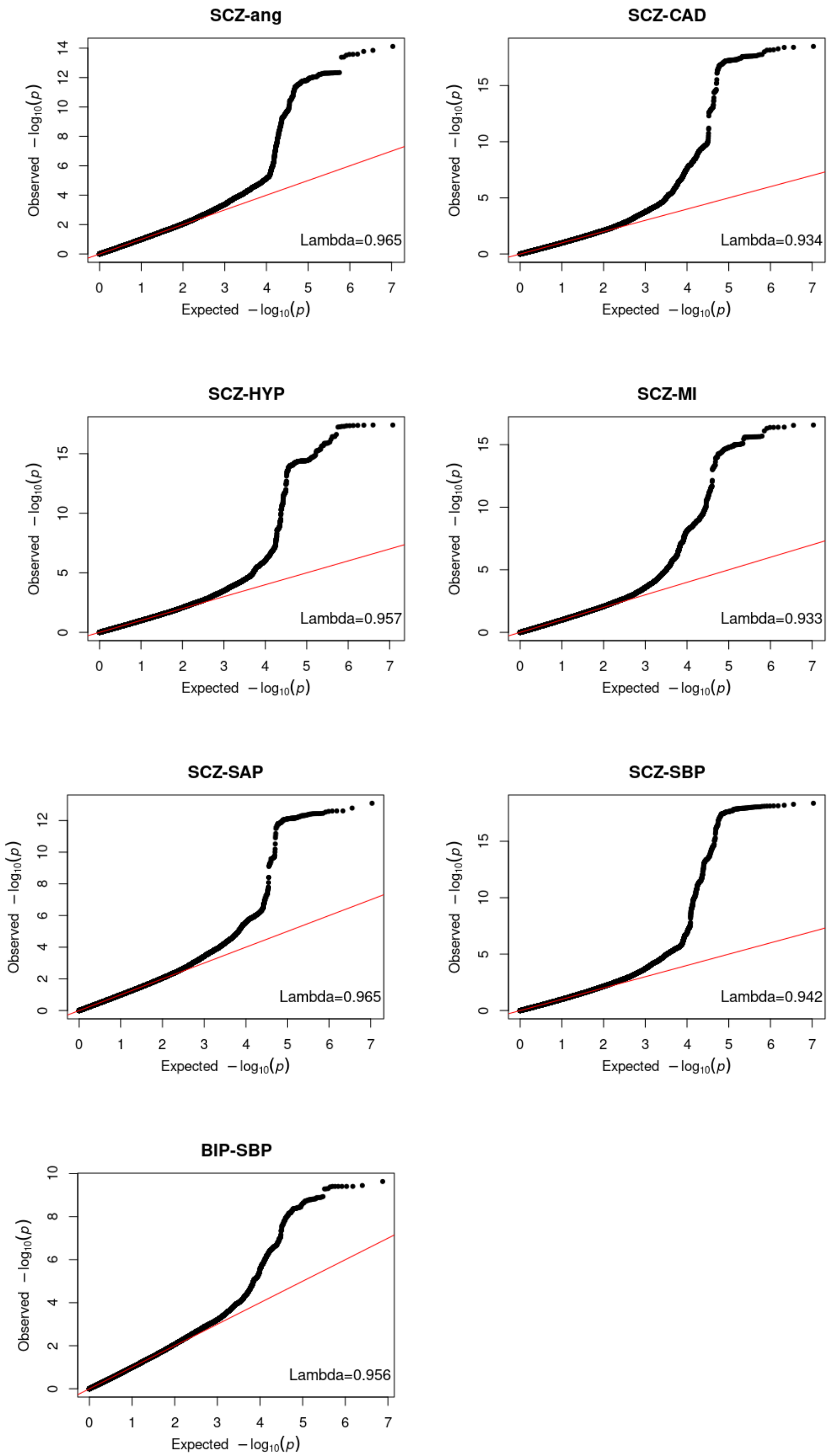

Supplemental Figure 3. QQ plots for the genome-wide pleiotropic analysis

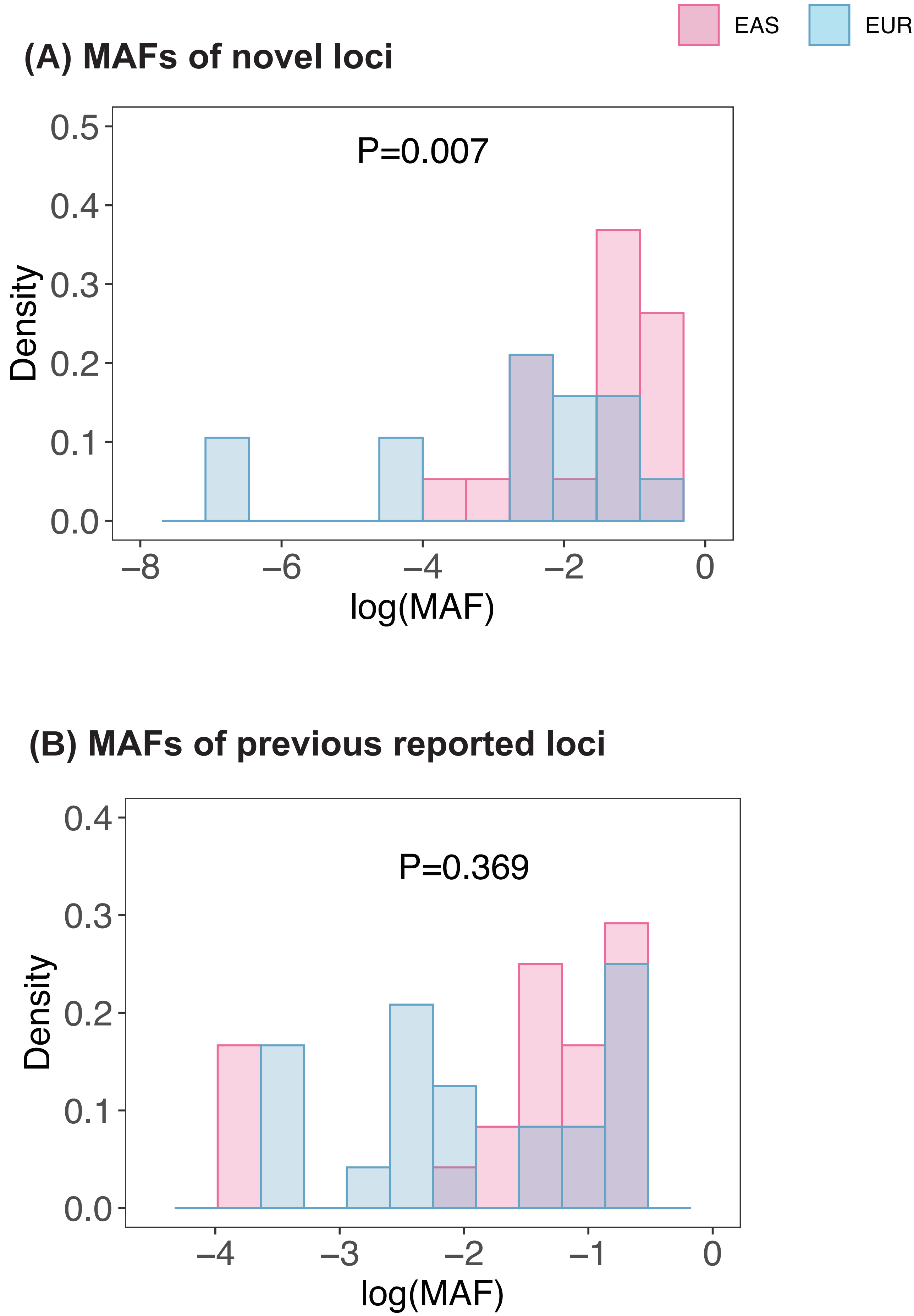

Supplemental Figure 4. Comparison of minor allele frequencies (MAFs) of the pleiotropic loci between East Asian (EAS) and European (EUR) populations

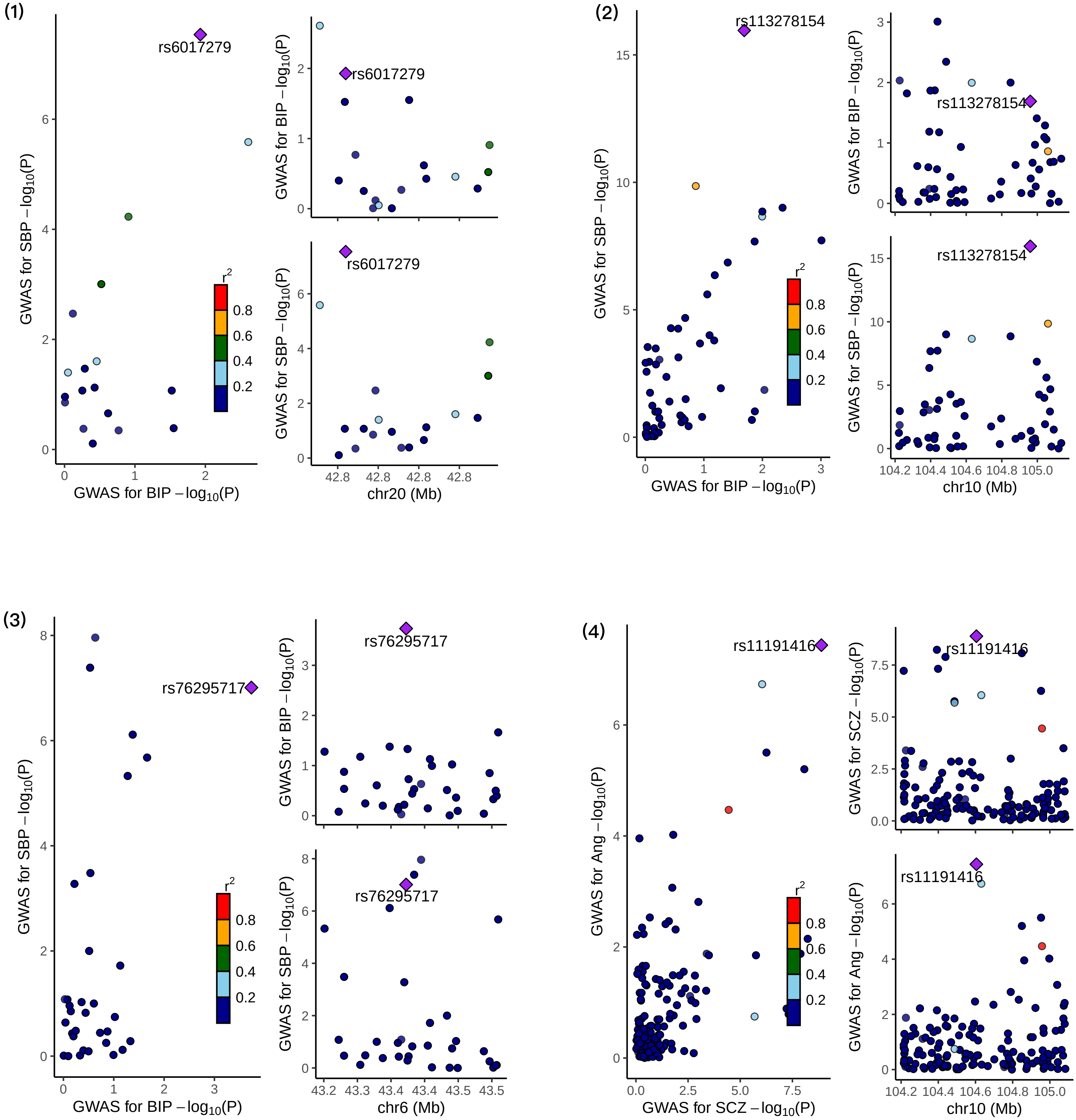
(1)
rs6017279
GWAS for SBP −log10(P)
GWAS for BIP −log10(P)
r2
0.8
0.6
0.4
0.2
chr20 (Mb)
(2)
rs113278154
GWAS for SBP −log10(P)
GWAS for BIP −log10(P)
chr10 (Mb)
(3)
rs76295717
GWAS for SBP −log10(P)
GWAS for BIP −log10(P)
chr6 (Mb)
(4)
rs11191416
GWAS for Ang −log10(P)
GWAS for SCZ −log10(P)
chr10 (Mb)

(5)

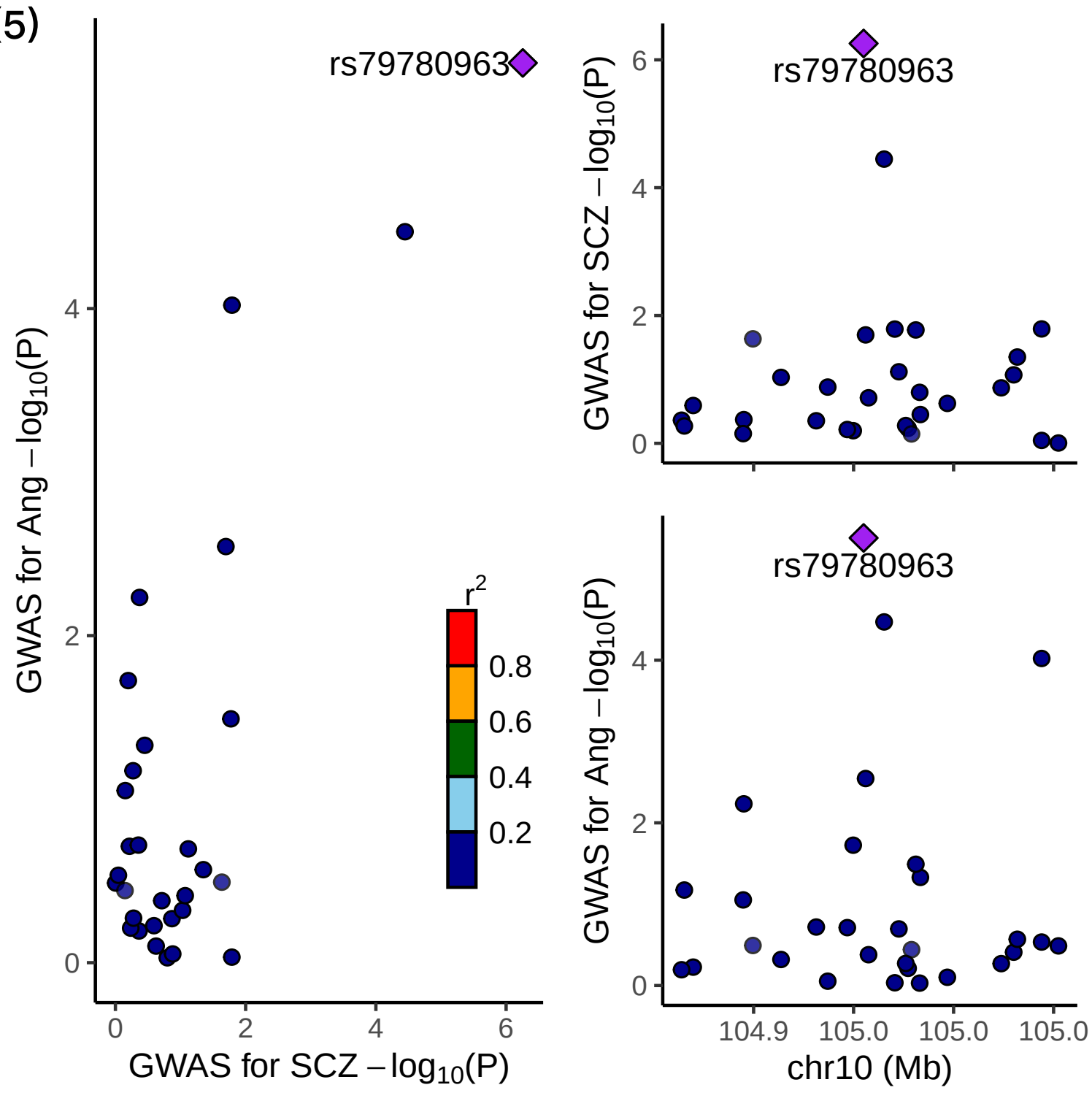
rs79780963
GWAS for Ang – log10(P)
GWAS for SCZ – log10(P)
GWAS for SCZ – log10(P)
GWAS for Ang – log10(P)
chr10 (Mb)
r2
0.8
0.6
0.4
0.2

(6)

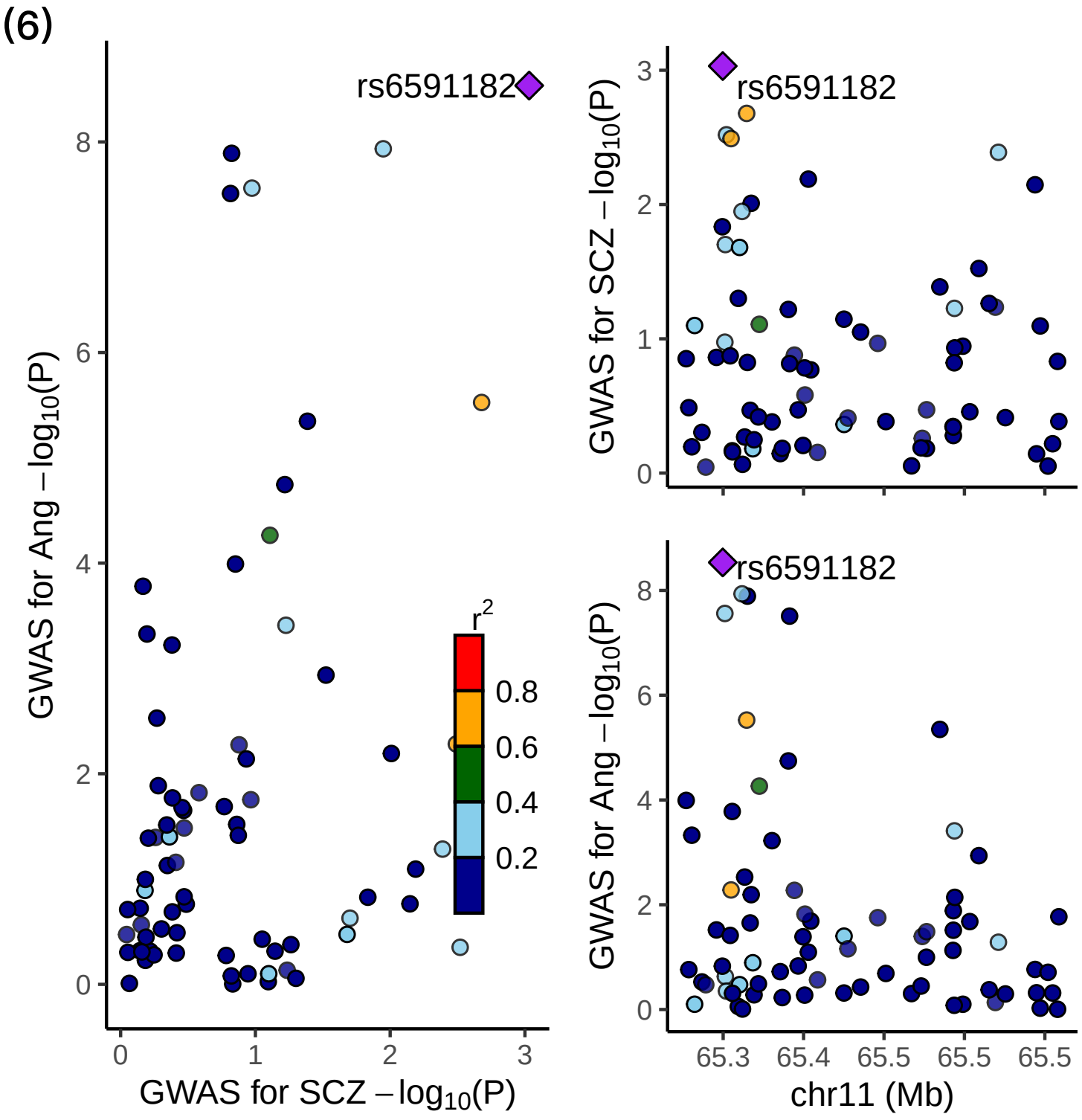
rs6591182
GWAS for Ang – log10(P)
GWAS for SCZ – log10(P)
GWAS for SCZ – log10(P)
GWAS for Ang – log10(P)
chr11 (Mb)
r2
0.8
0.6
0.4
0.2

(7)

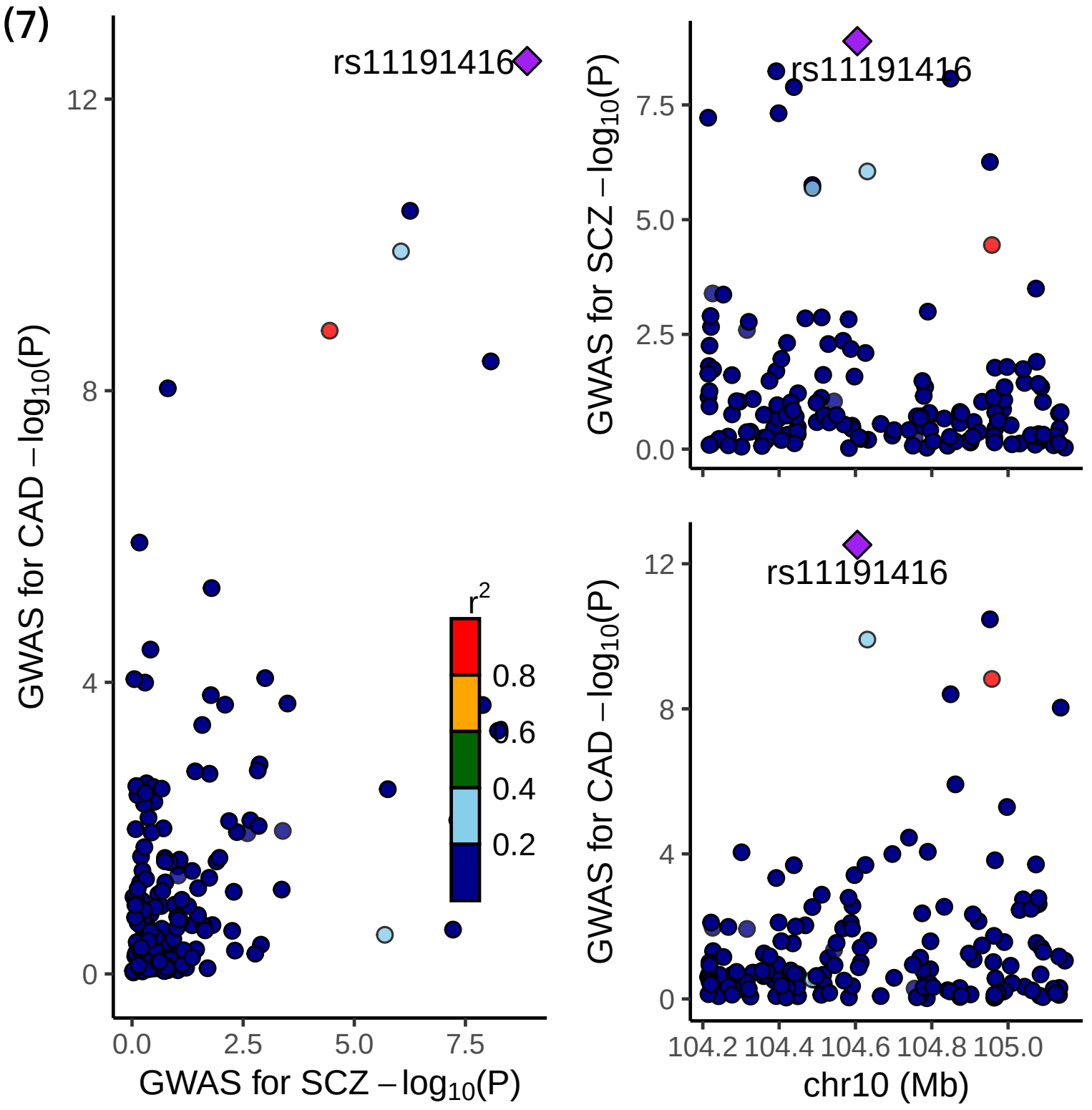
rs11191416
GWAS for CAD – log10(P)
GWAS for SCZ – log10(P)
GWAS for SCZ – log10(P)
GWAS for CAD – log10(P)
chr10 (Mb)
r2
0.8
0.6
0.4
0.2

(8)

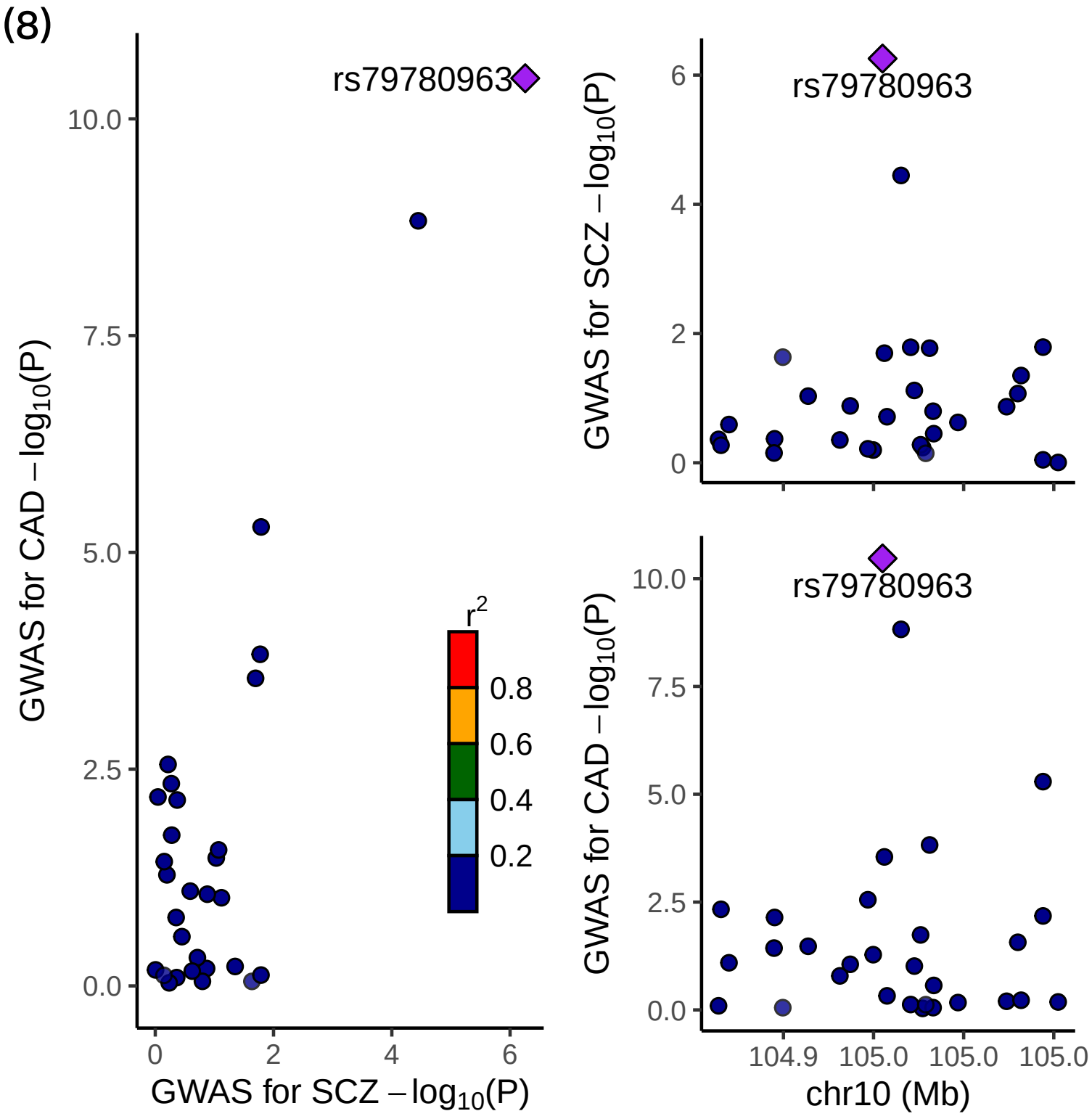
rs79780963
GWAS for CAD – log10(P)
GWAS for SCZ – log10(P)
GWAS for SCZ – log10(P)
GWAS for CAD – log10(P)
chr10 (Mb)
r2
0.8
0.6
0.4
0.2

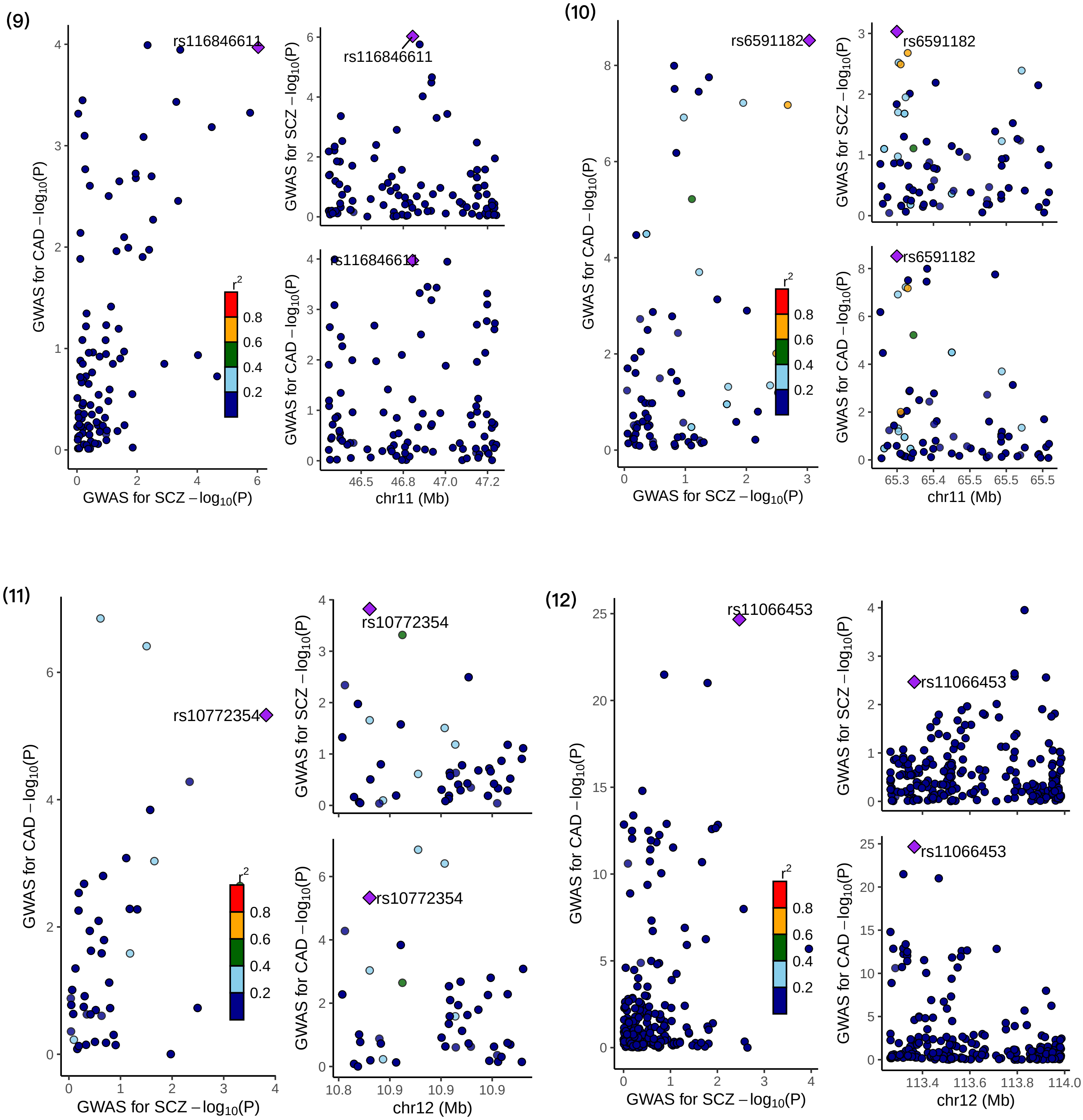
(9)
rs116846611
GWAS for CAD −log10(P)
GWAS for SCZ −log10(P)
GWAS for SCZ −log10(P)
GWAS for CAD −log10(P)
chr11 (Mb)
r2
0.8
0.6
0.4
0.2
(10)
rs6591182
GWAS for CAD −log10(P)
GWAS for SCZ −log10(P)
GWAS for SCZ −log10(P)
GWAS for CAD −log10(P)
chr11 (Mb)
(11)
rs10772354
GWAS for CAD −log10(P)
GWAS for SCZ −log10(P)
GWAS for SCZ −log10(P)
GWAS for CAD −log10(P)
chr12 (Mb)
(12)
rs11066453
GWAS for CAD −log10(P)
GWAS for SCZ −log10(P)
GWAS for SCZ −log10(P)
GWAS for CAD −log10(P)
chr12 (Mb)

(13)

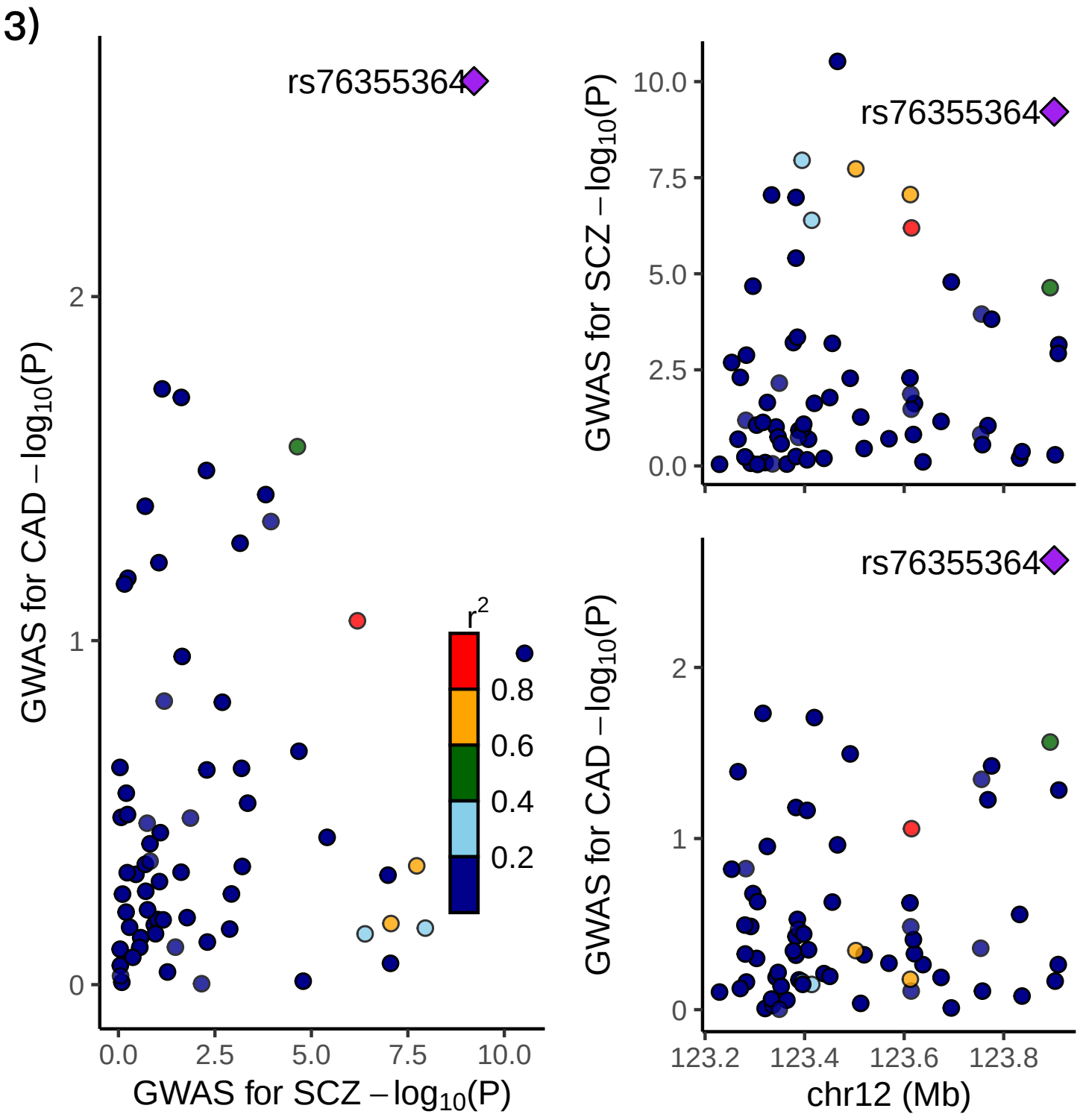
rs76355364
GWAS for CAD −log10(P)
GWAS for SCZ −log10(P)
r2
0.8
0.6
0.4
0.2
GWAS for SCZ −log10(P)
GWAS for CAD −log10(P)
chr12 (Mb)

(14)

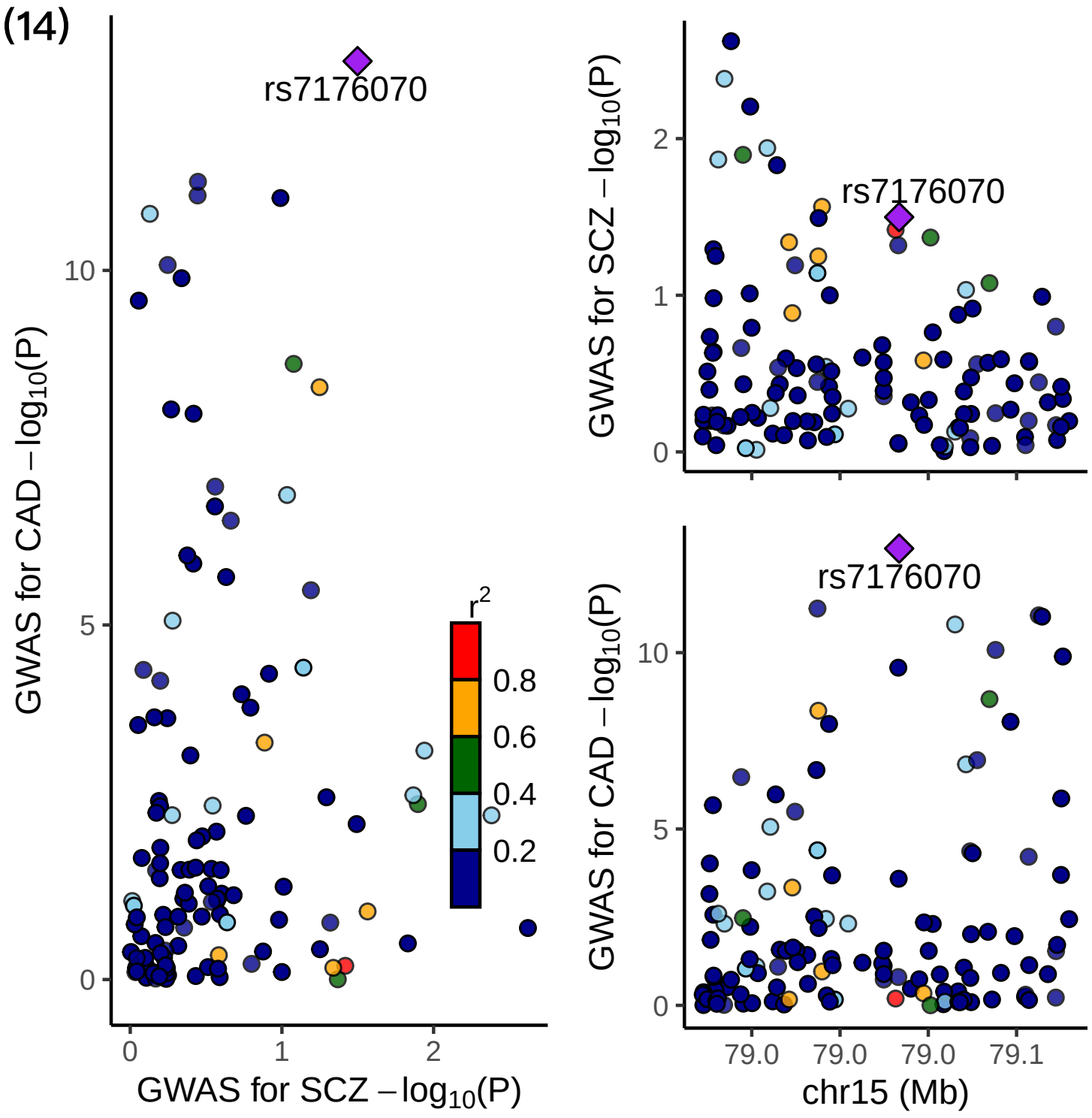
rs7176070
GWAS for CAD −log10(P)
GWAS for SCZ −log10(P)
r2
0.8
0.6
0.4
0.2
GWAS for SCZ −log10(P)
GWAS for CAD −log10(P)
chr15 (Mb)

(15)

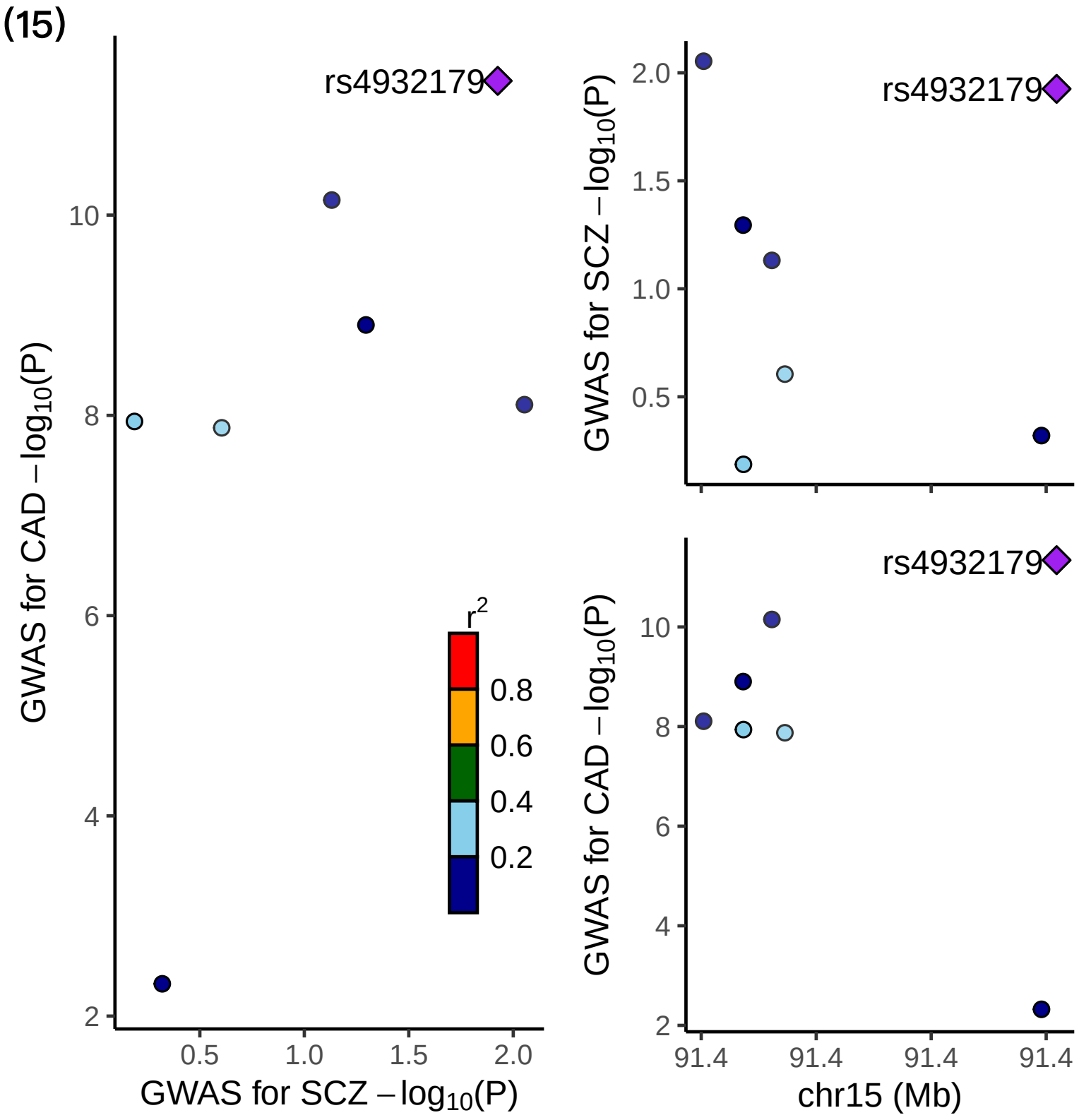
rs4932179
GWAS for CAD −log10(P)
GWAS for SCZ −log10(P)
r2
0.8
0.6
0.4
0.2
GWAS for SCZ −log10(P)
GWAS for CAD −log10(P)
chr15 (Mb)

(16)

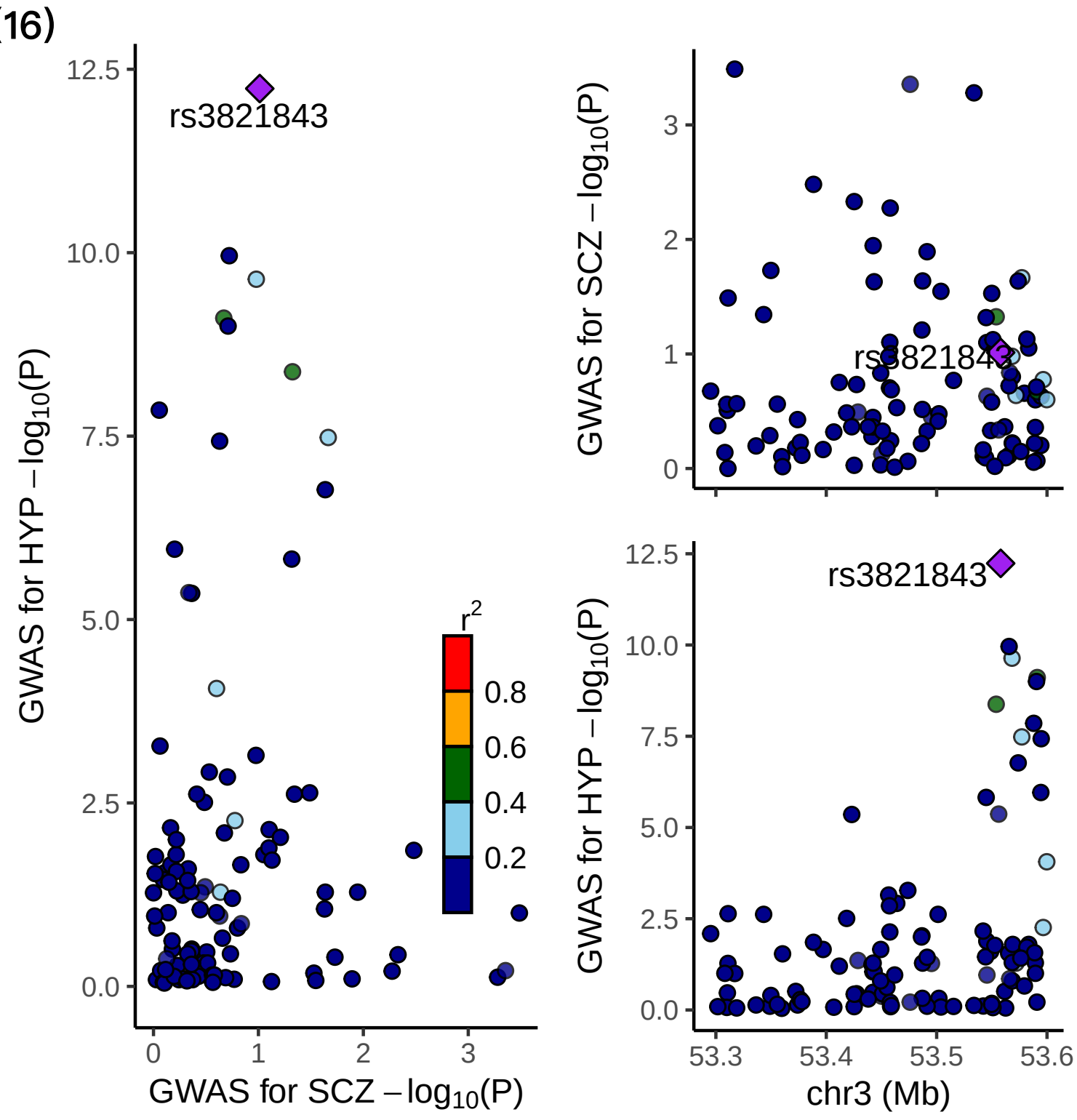
rs3821843
GWAS for HYP −log10(P)
GWAS for SCZ −log10(P)
r2
0.8
0.6
0.4
0.2
GWAS for SCZ −log10(P)
GWAS for HYP −log10(P)
chr3 (Mb)

(17)

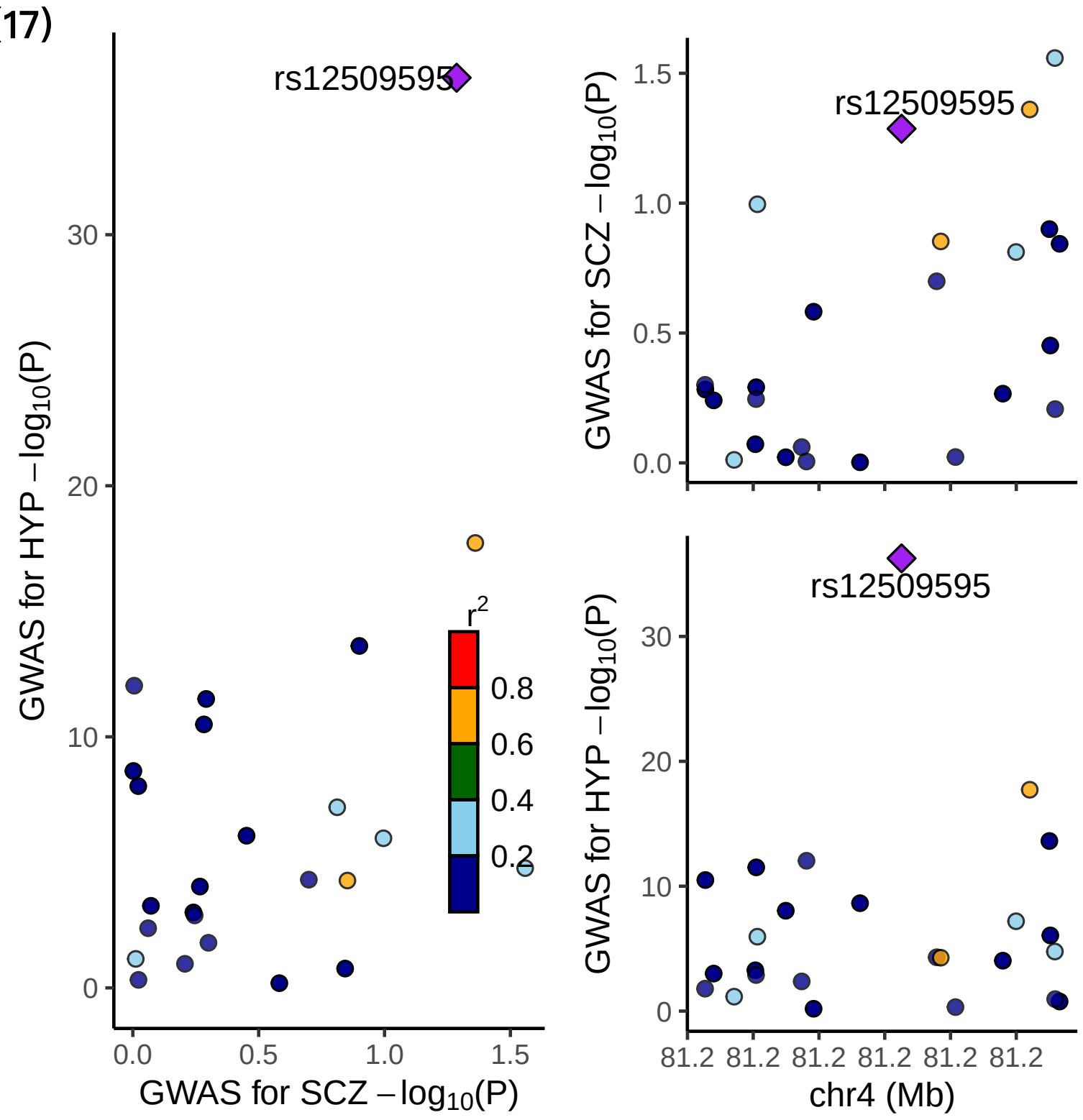
rs12509595
GWAS for HYP $-\log_{10}(P)$
GWAS for SCZ $-\log_{10}(P)$
GWAS for SCZ $-\log_{10}(P)$
GWAS for HYP $-\log_{10}(P)$
chr4 (Mb)
r^2
0.8
0.6
0.4
0.2

(18)

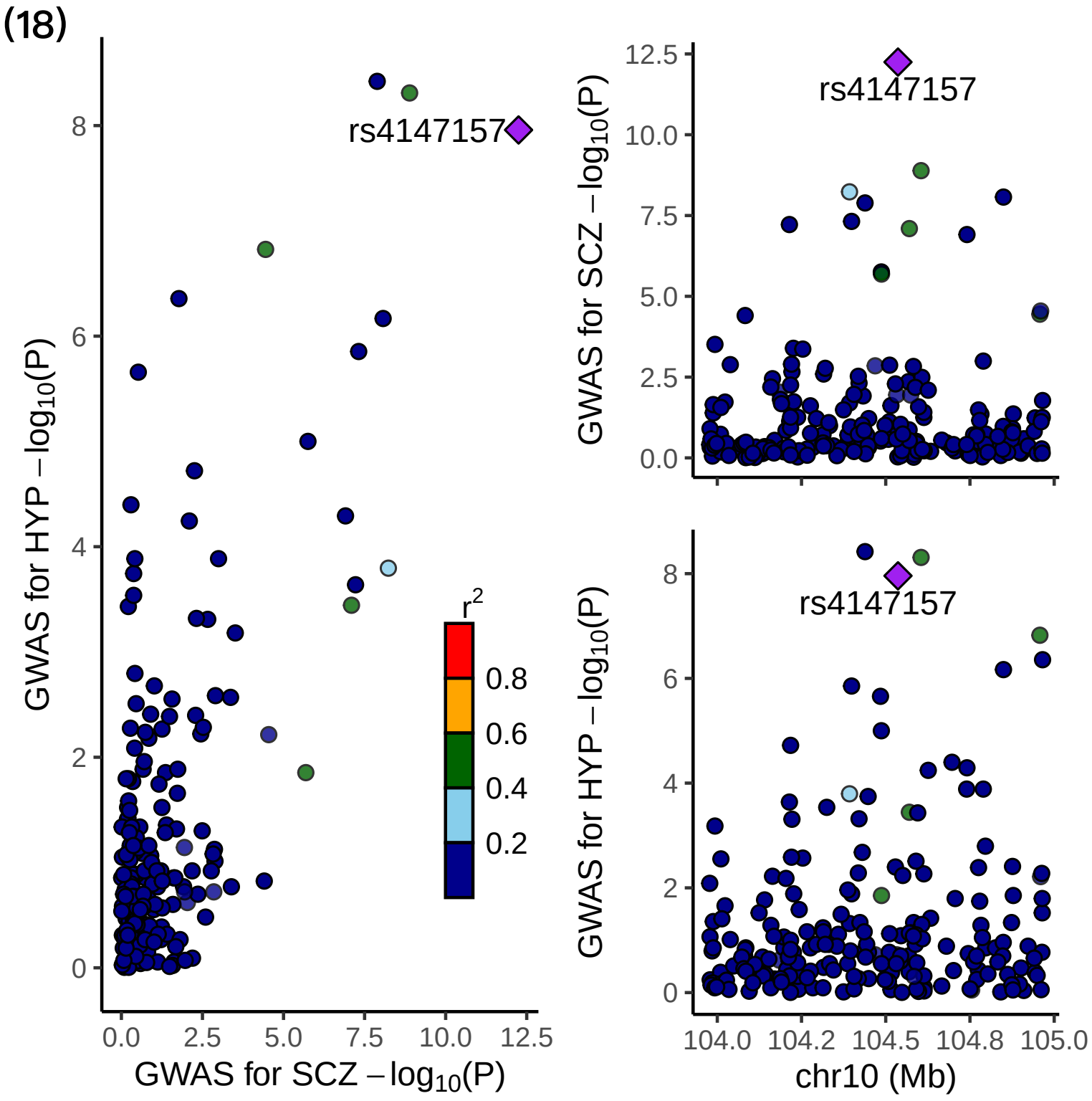
rs4147157
GWAS for HYP $-\log_{10}(P)$
GWAS for SCZ $-\log_{10}(P)$
GWAS for SCZ $-\log_{10}(P)$
GWAS for HYP $-\log_{10}(P)$
chr10 (Mb)
r^2
0.8
0.6
0.4
0.2

(19)

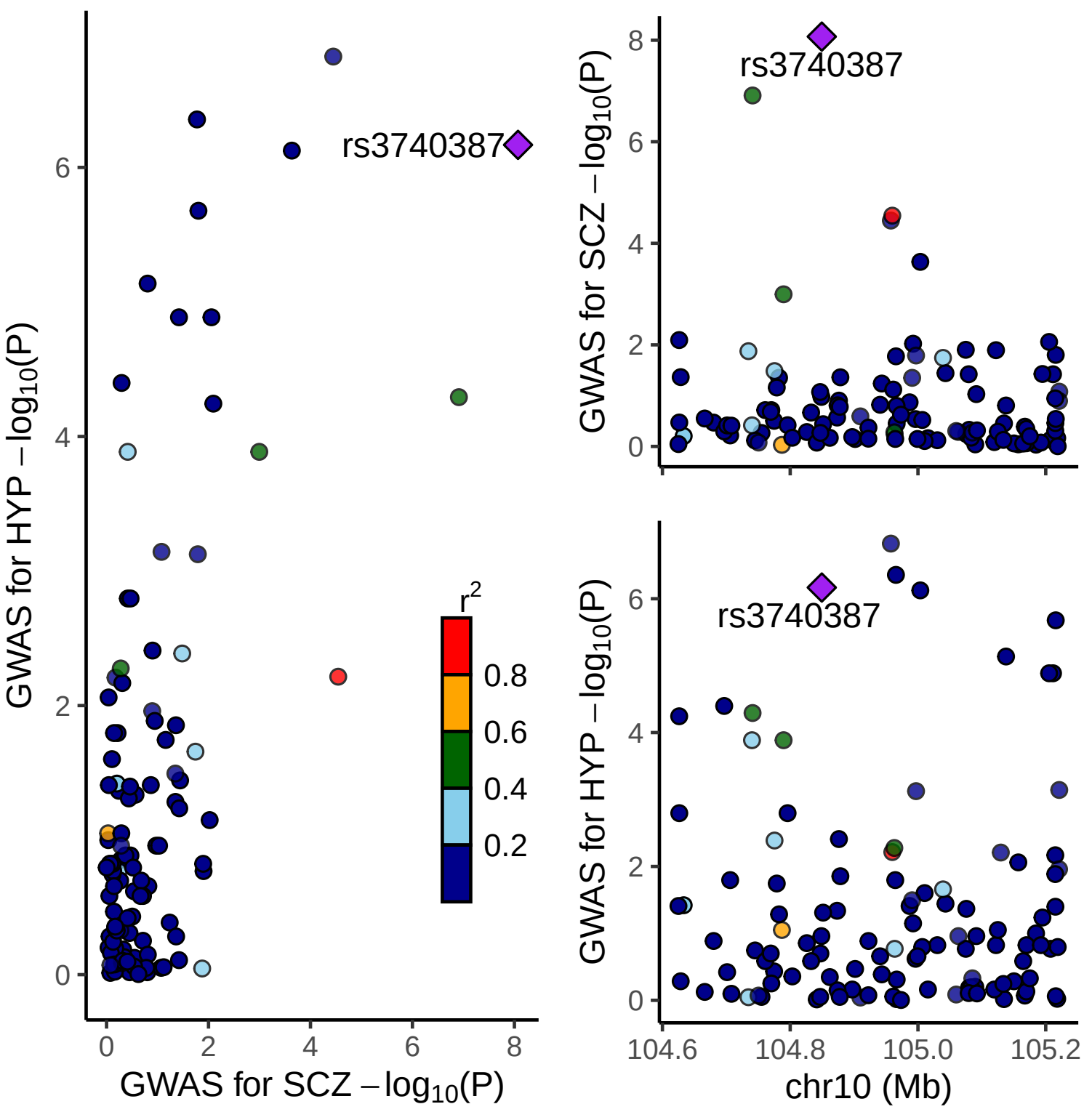
rs3740387
GWAS for HYP $-\log_{10}(P)$
GWAS for SCZ $-\log_{10}(P)$
GWAS for SCZ $-\log_{10}(P)$
GWAS for HYP $-\log_{10}(P)$
chr10 (Mb)
r^2
0.8
0.6
0.4
0.2

(20)

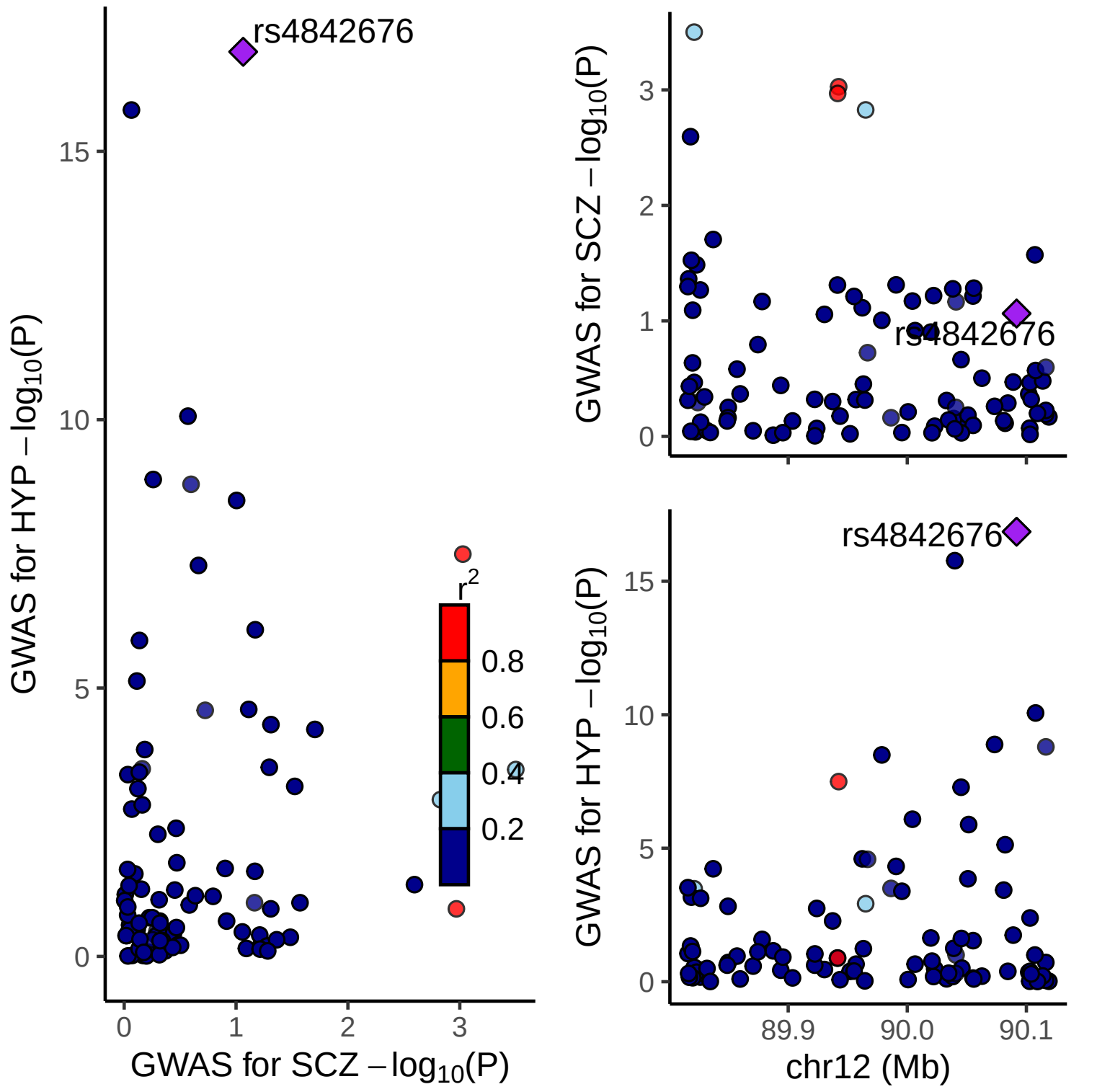
rs4842676
GWAS for HYP $-\log_{10}(P)$
GWAS for SCZ $-\log_{10}(P)$
GWAS for SCZ $-\log_{10}(P)$
GWAS for HYP $-\log_{10}(P)$
chr12 (Mb)
r^2
0.8
0.6
0.4
0.2

(21)

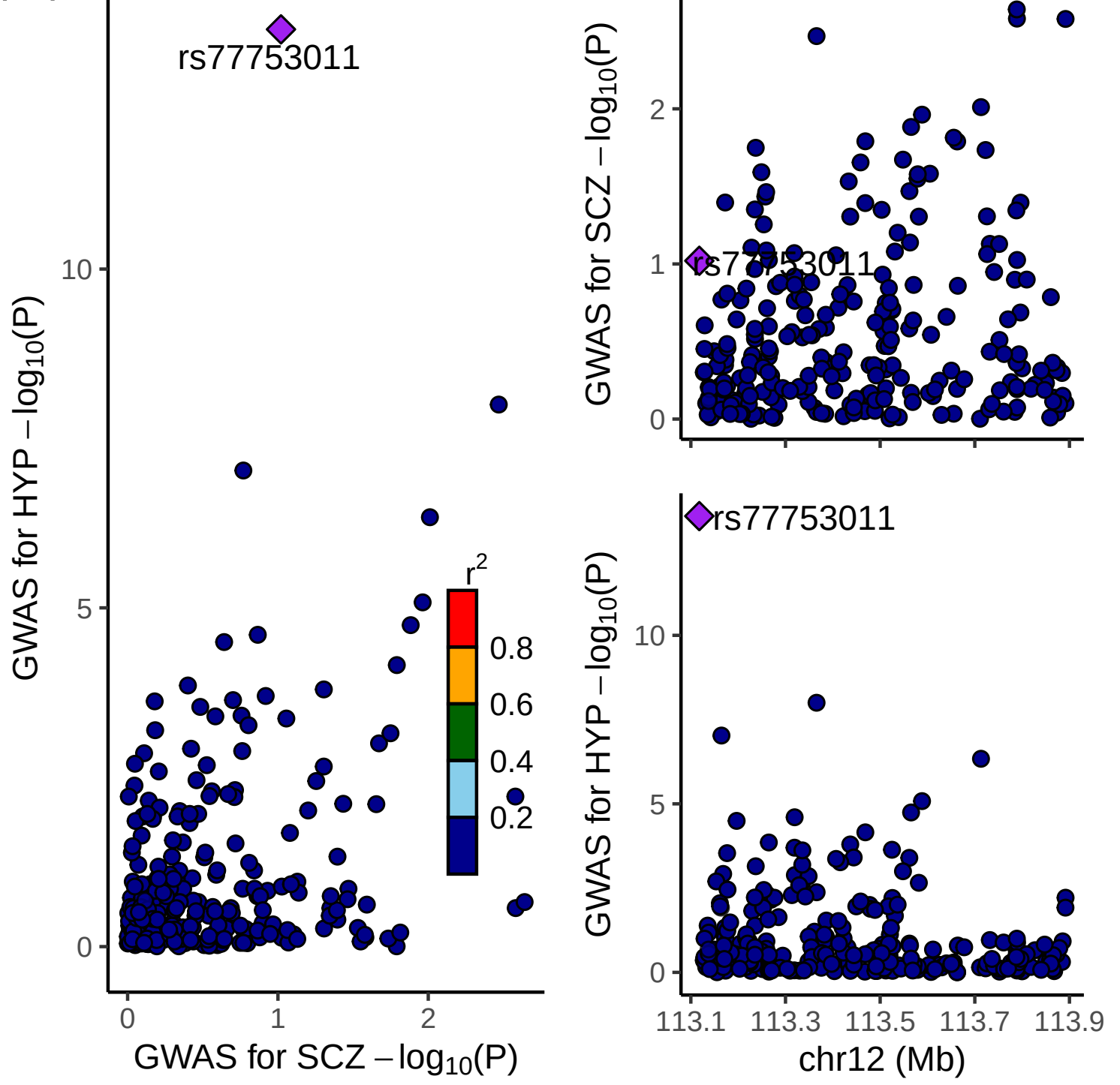
rs77753011
GWAS for HYP −log10(P)
GWAS for SCZ −log10(P)
r2
0.8
0.6
0.4
0.2
GWAS for SCZ −log10(P)
GWAS for HYP −log10(P)
113.1 113.3 113.5 113.7 113.9
chr12 (Mb)

(22)

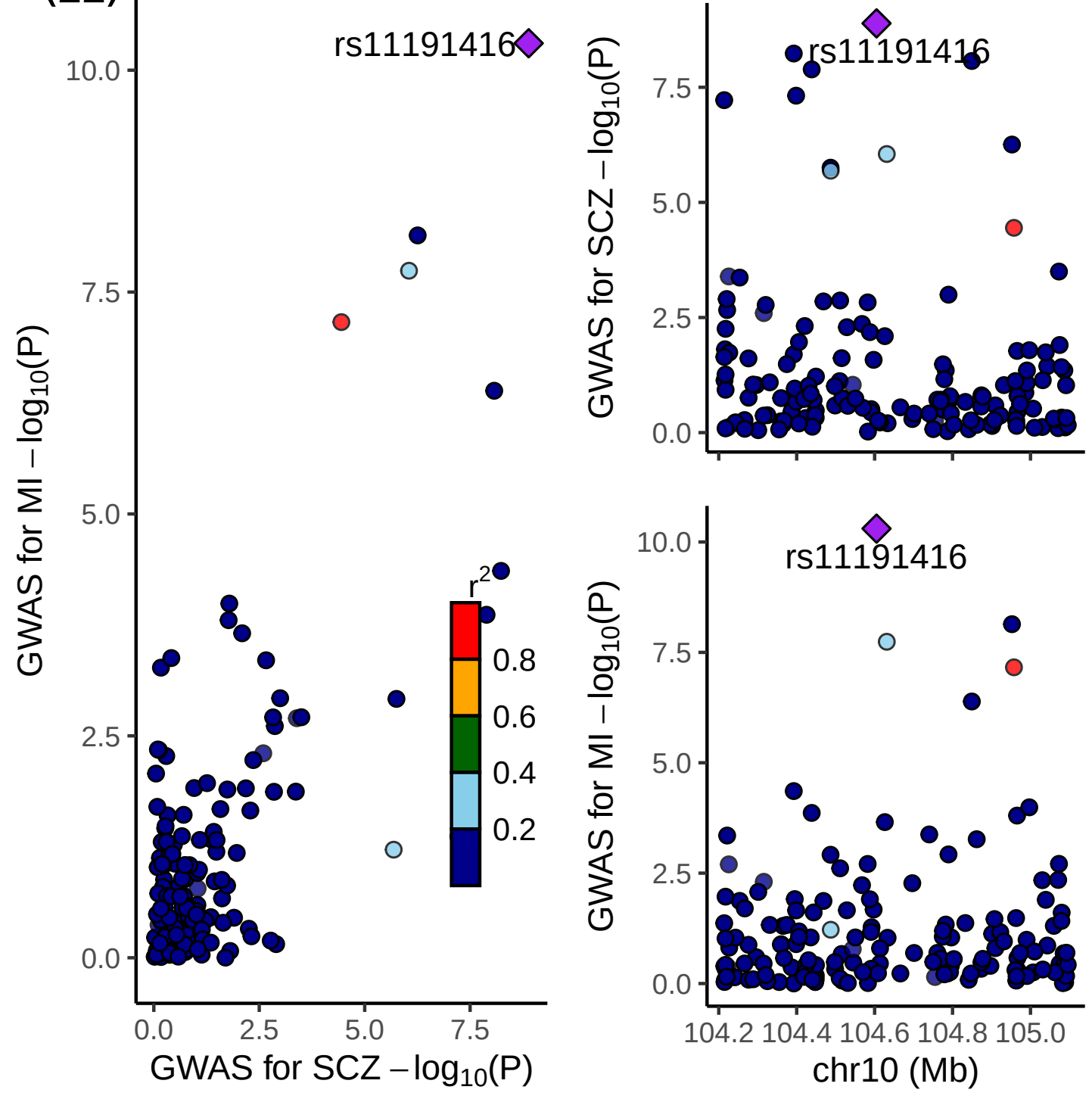
rs11191416
GWAS for MI −log10(P)
GWAS for SCZ −log10(P)
r2
0.8
0.6
0.4
0.2
GWAS for SCZ −log10(P)
GWAS for MI −log10(P)
104.2 104.4 104.6 104.8 105.0
chr10 (Mb)

(23)

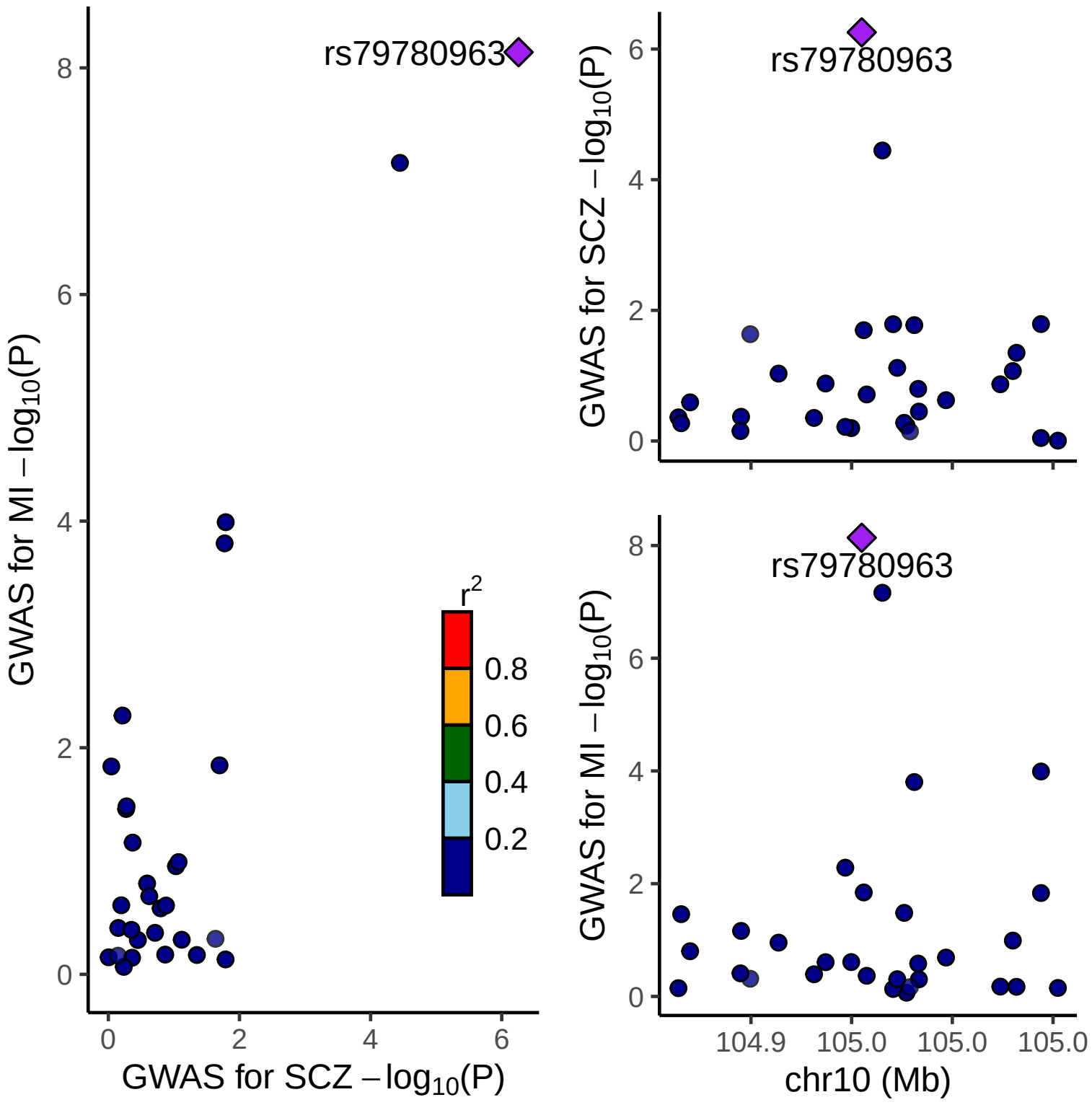
rs79780963
GWAS for MI −log10(P)
GWAS for SCZ −log10(P)
r2
0.8
0.6
0.4
0.2
GWAS for SCZ −log10(P)
GWAS for MI −log10(P)
104.9 105.0 105.0 105.0
chr10 (Mb)

(24)

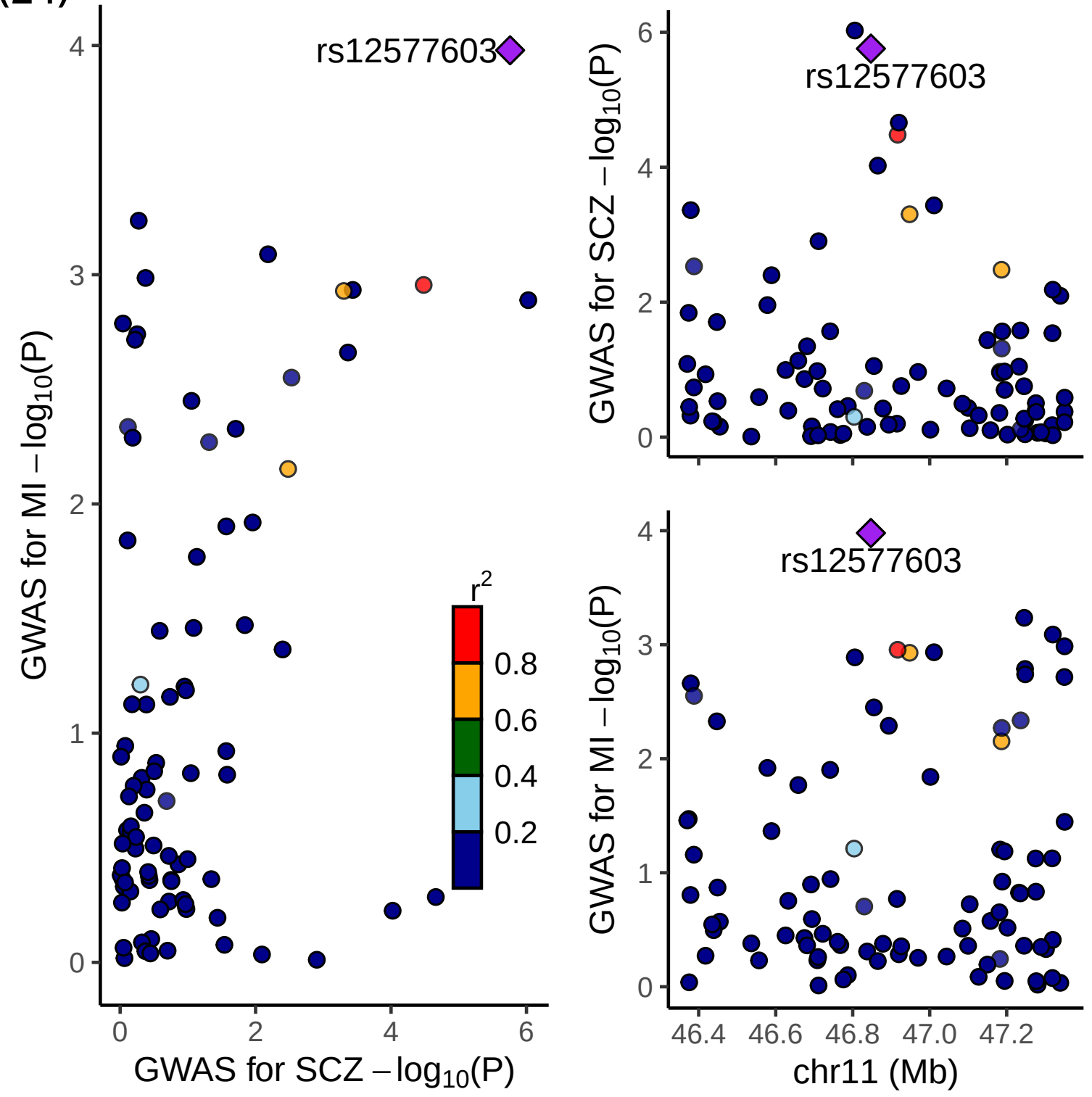
rs12577603
GWAS for MI −log10(P)
GWAS for SCZ −log10(P)
r2
0.8
0.6
0.4
0.2
GWAS for SCZ −log10(P)
GWAS for MI −log10(P)
46.4 46.6 46.8 47.0 47.2
chr11 (Mb)

(25)

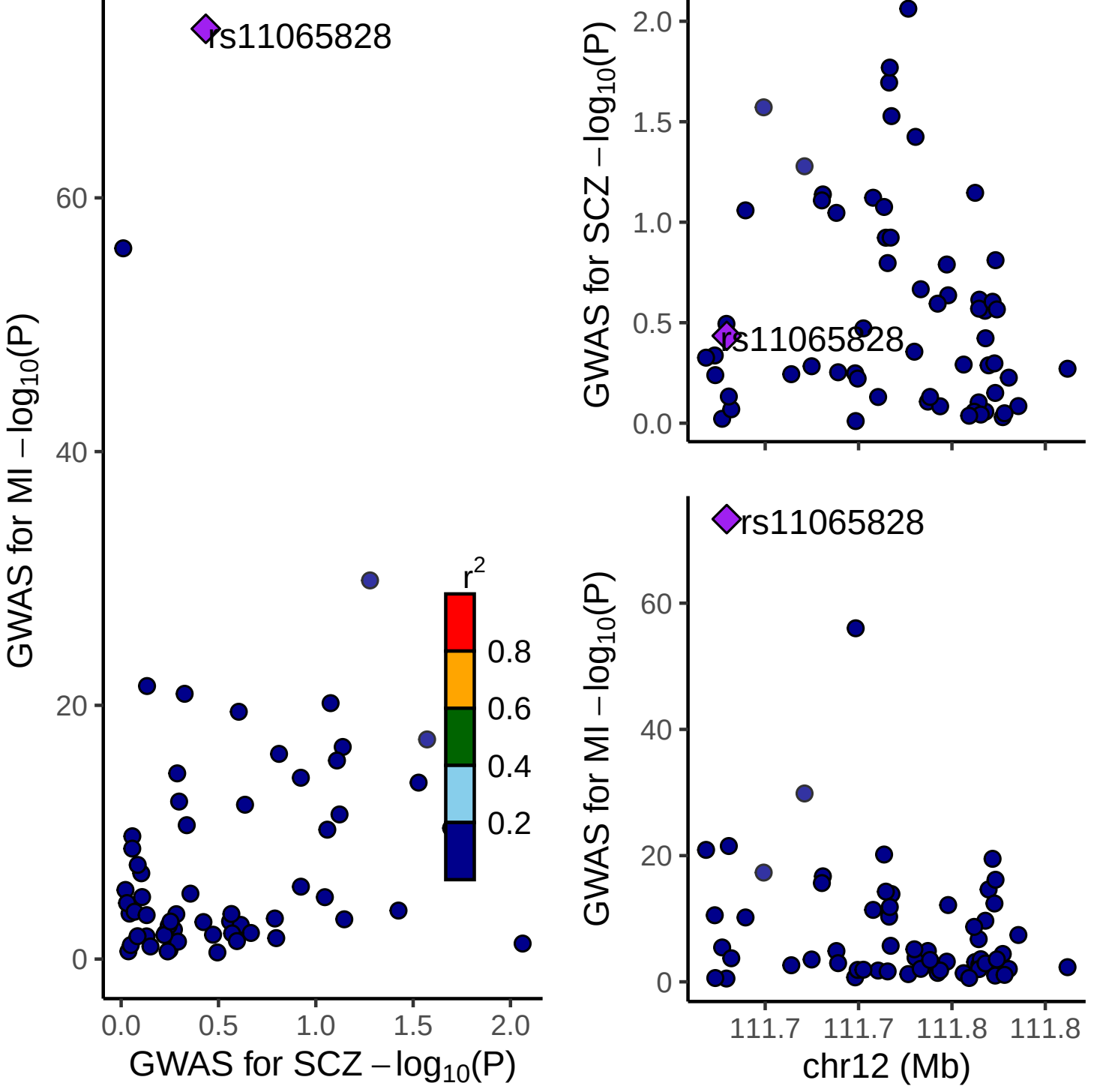
rs11065828
GWAS for MI −log10(P)
GWAS for SCZ −log10(P)
r2
GWAS for SCZ −log10(P)
GWAS for MI −log10(P)
chr12 (Mb)

(26)

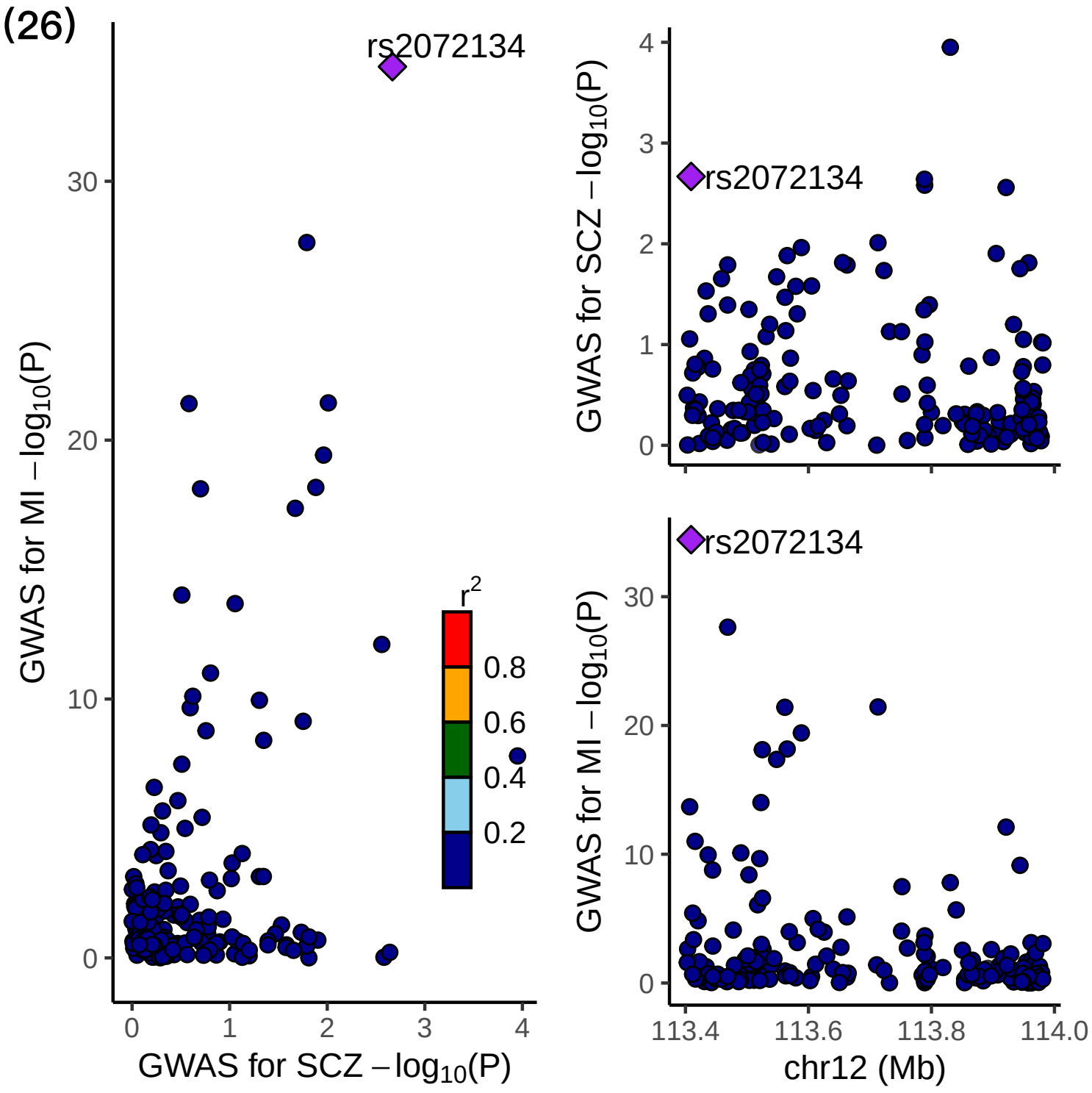
rs2072134
GWAS for MI −log10(P)
GWAS for SCZ −log10(P)
r2
GWAS for SCZ −log10(P)
GWAS for MI −log10(P)
chr12 (Mb)

(27)

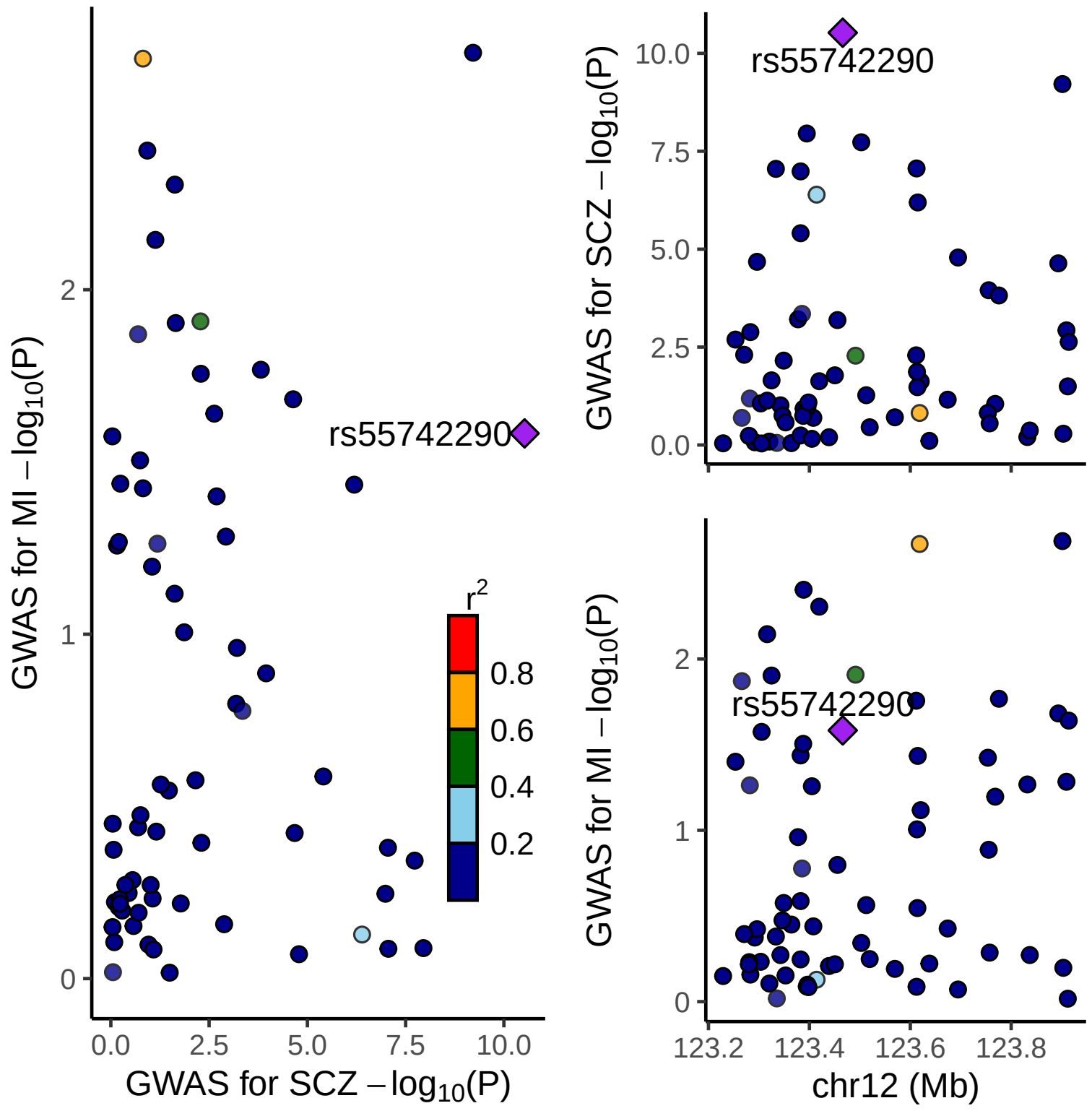
rs55742290
GWAS for MI −log10(P)
GWAS for SCZ −log10(P)
r2
GWAS for SCZ −log10(P)
GWAS for MI −log10(P)
chr12 (Mb)

(28)

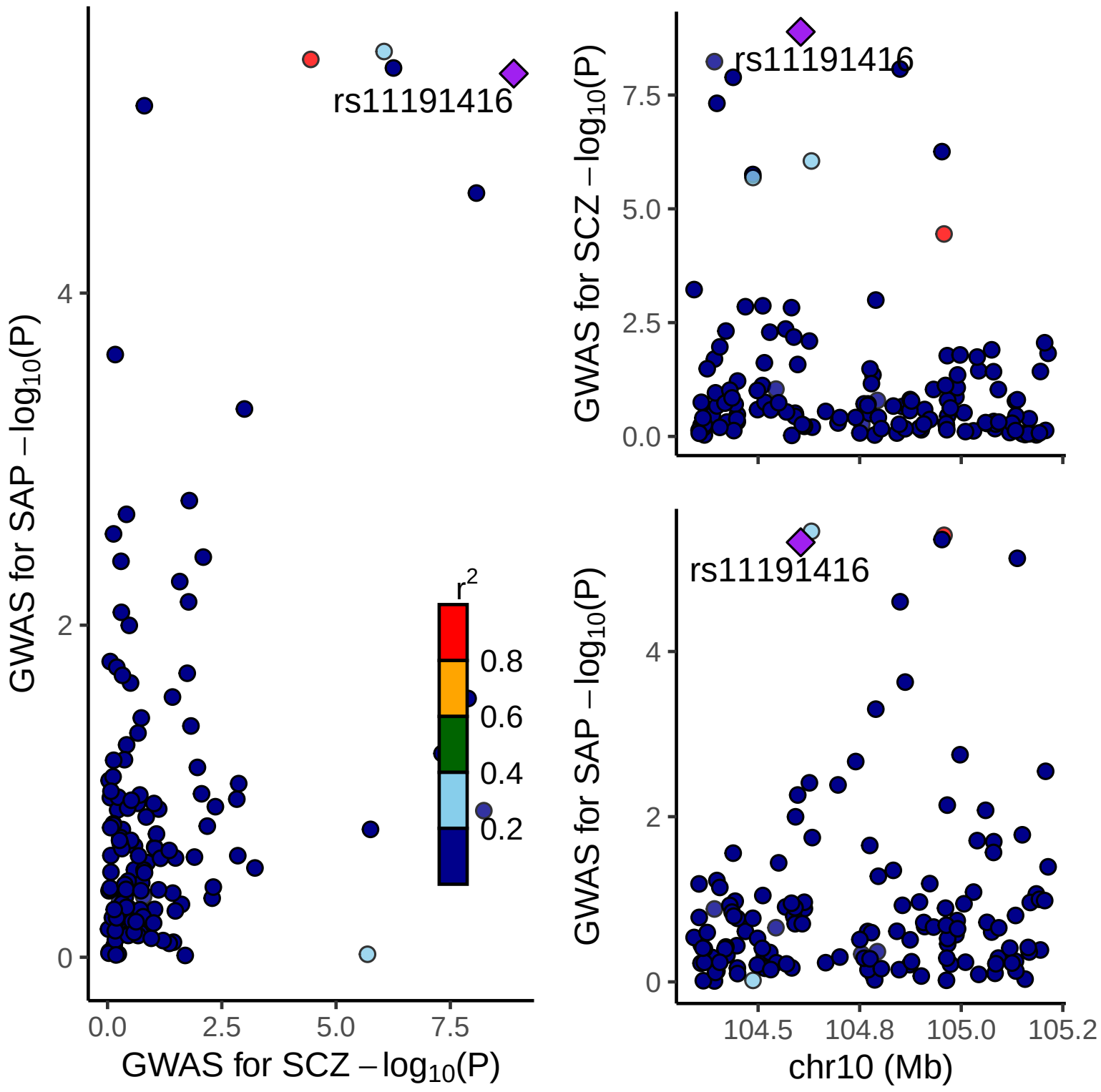
rs11191416
GWAS for SAP −log10(P)
GWAS for SCZ −log10(P)
r2
GWAS for SCZ −log10(P)
GWAS for SAP −log10(P)
chr10 (Mb)

(29)

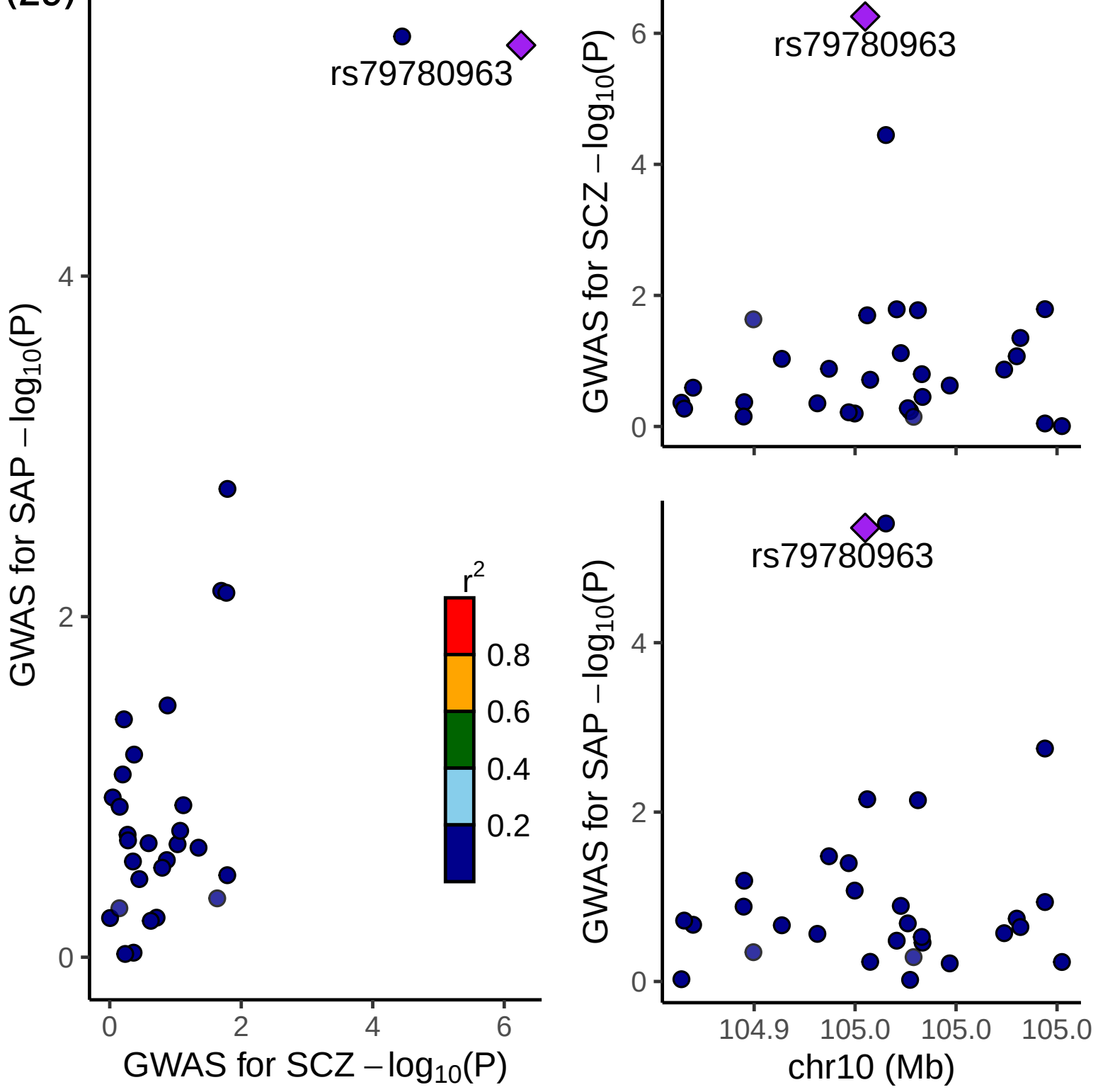
rs79780963
GWAS for SAP – log10(P)
GWAS for SCZ – log10(P)
r2
0.8
0.6
0.4
0.2
rs79780963
GWAS for SCZ – log10(P)
rs79780963
GWAS for SAP – log10(P)
104.9
105.0
105.0
105.0
chr10 (Mb)

(30)

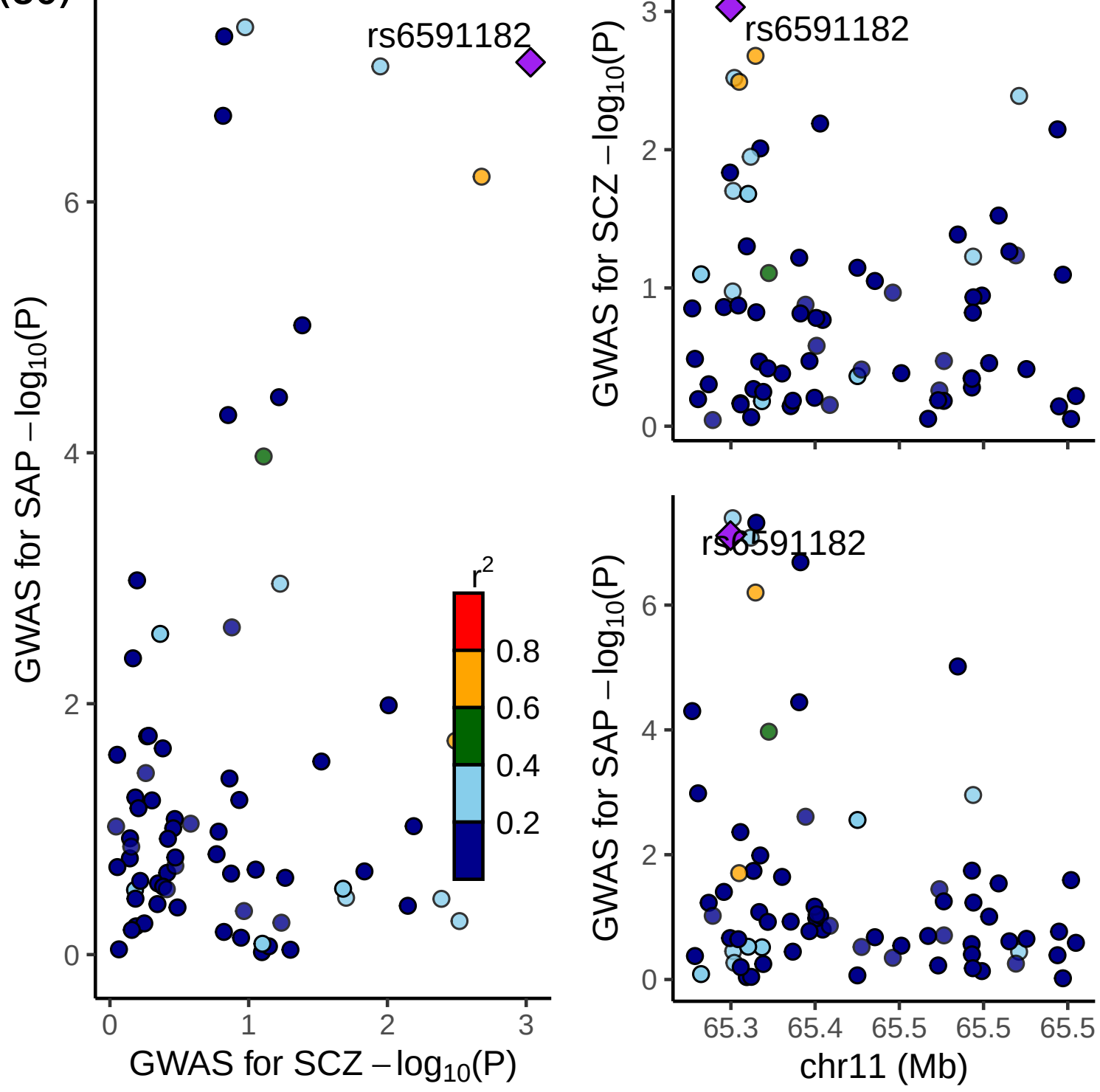
rs6591182
GWAS for SAP – log10(P)
GWAS for SCZ – log10(P)
r2
0.8
0.6
0.4
0.2
rs6591182
GWAS for SCZ – log10(P)
rs6591182
GWAS for SAP – log10(P)
65.3
65.4
65.5
65.5
65.5
chr11 (Mb)

(31)

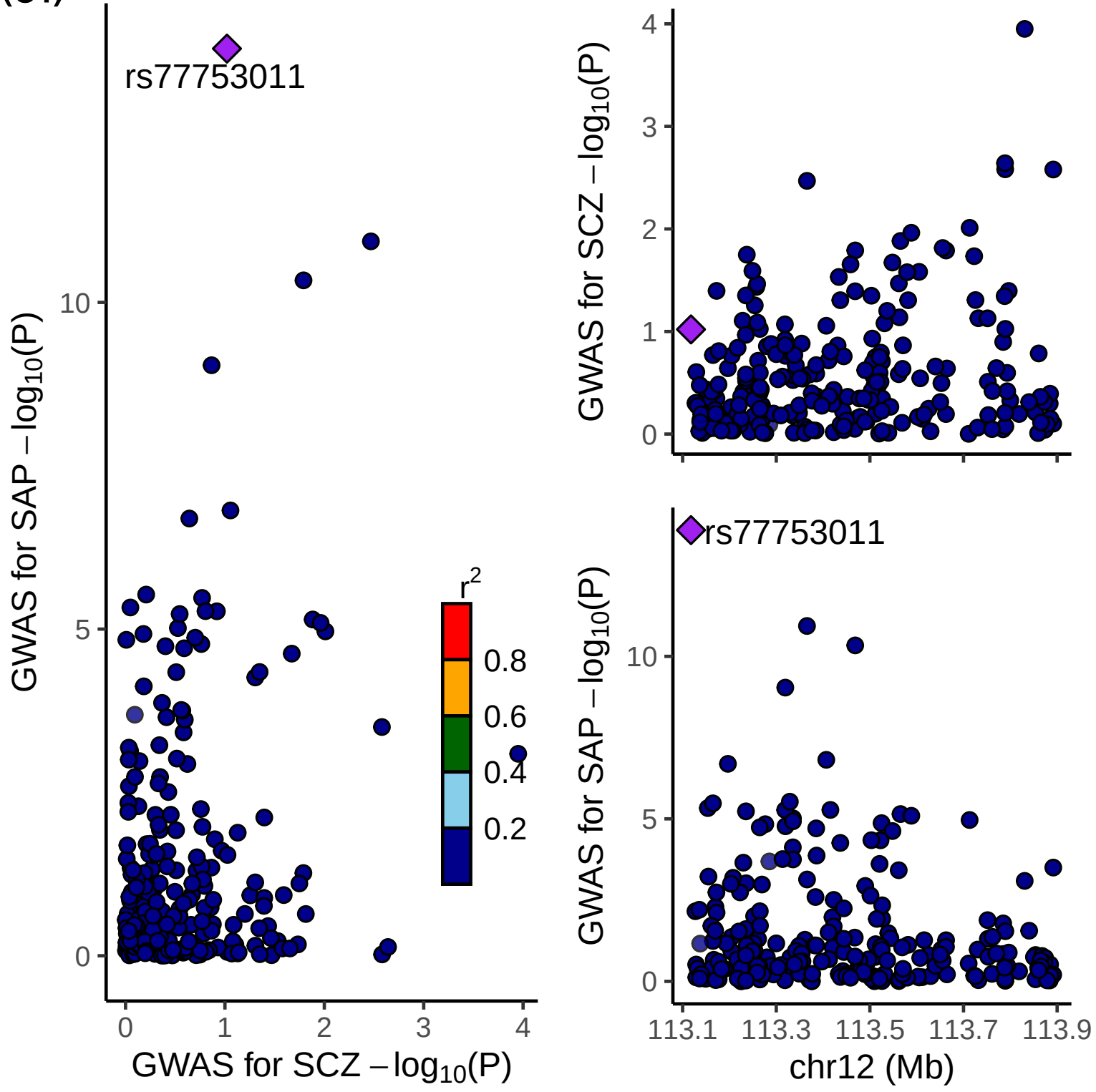
rs77753011
GWAS for SAP – log10(P)
GWAS for SCZ – log10(P)
r2
0.8
0.6
0.4
0.2
GWAS for SCZ – log10(P)
rs77753011
GWAS for SAP – log10(P)
113.1
113.3
113.5
113.7
113.9
chr12 (Mb)

(32)

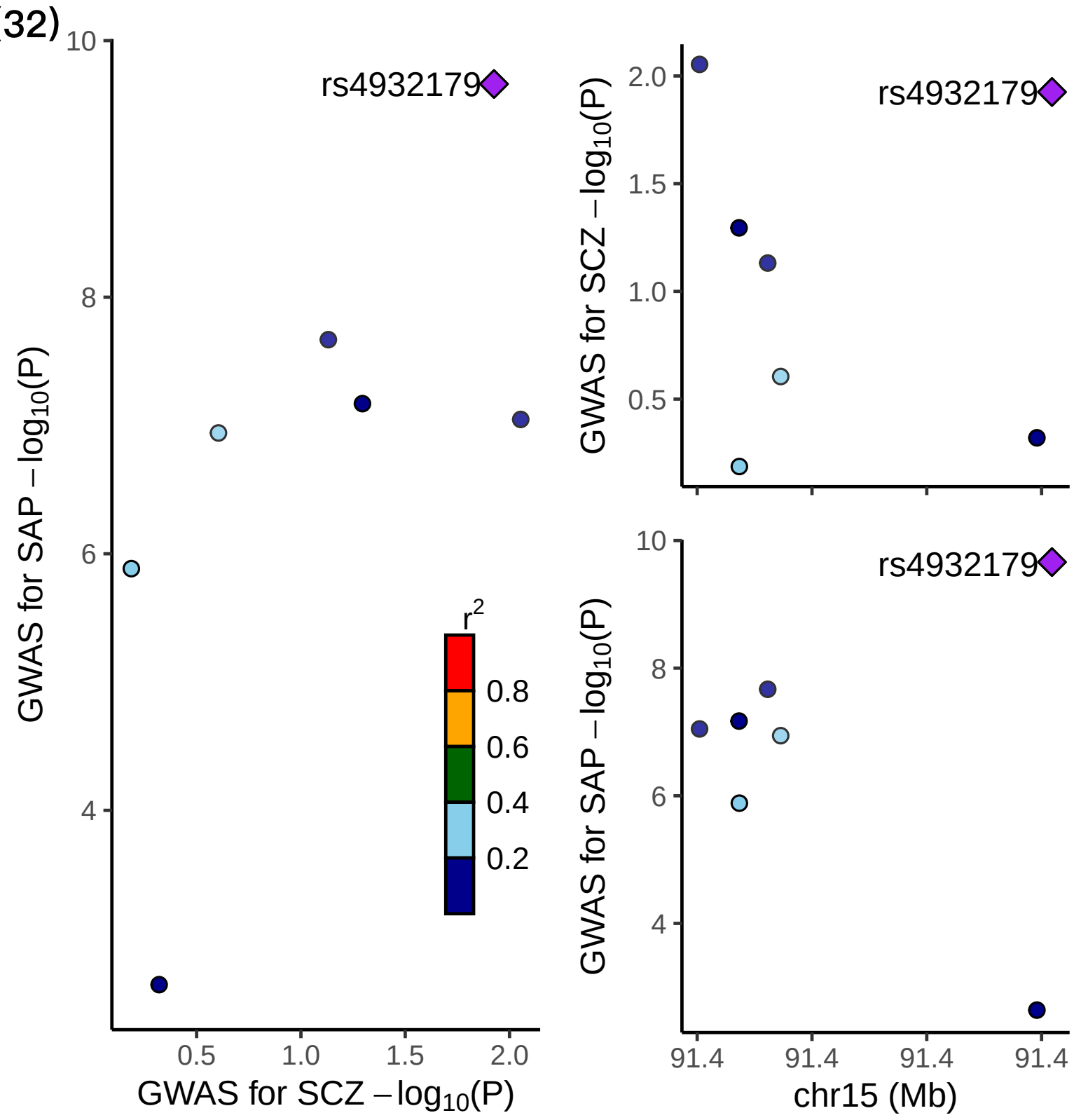
rs4932179
GWAS for SAP – log10(P)
GWAS for SCZ – log10(P)
r2
0.8
0.6
0.4
0.2
rs4932179
GWAS for SCZ – log10(P)
rs4932179
GWAS for SAP – log10(P)
91.4
91.4
91.4
91.4
chr15 (Mb)

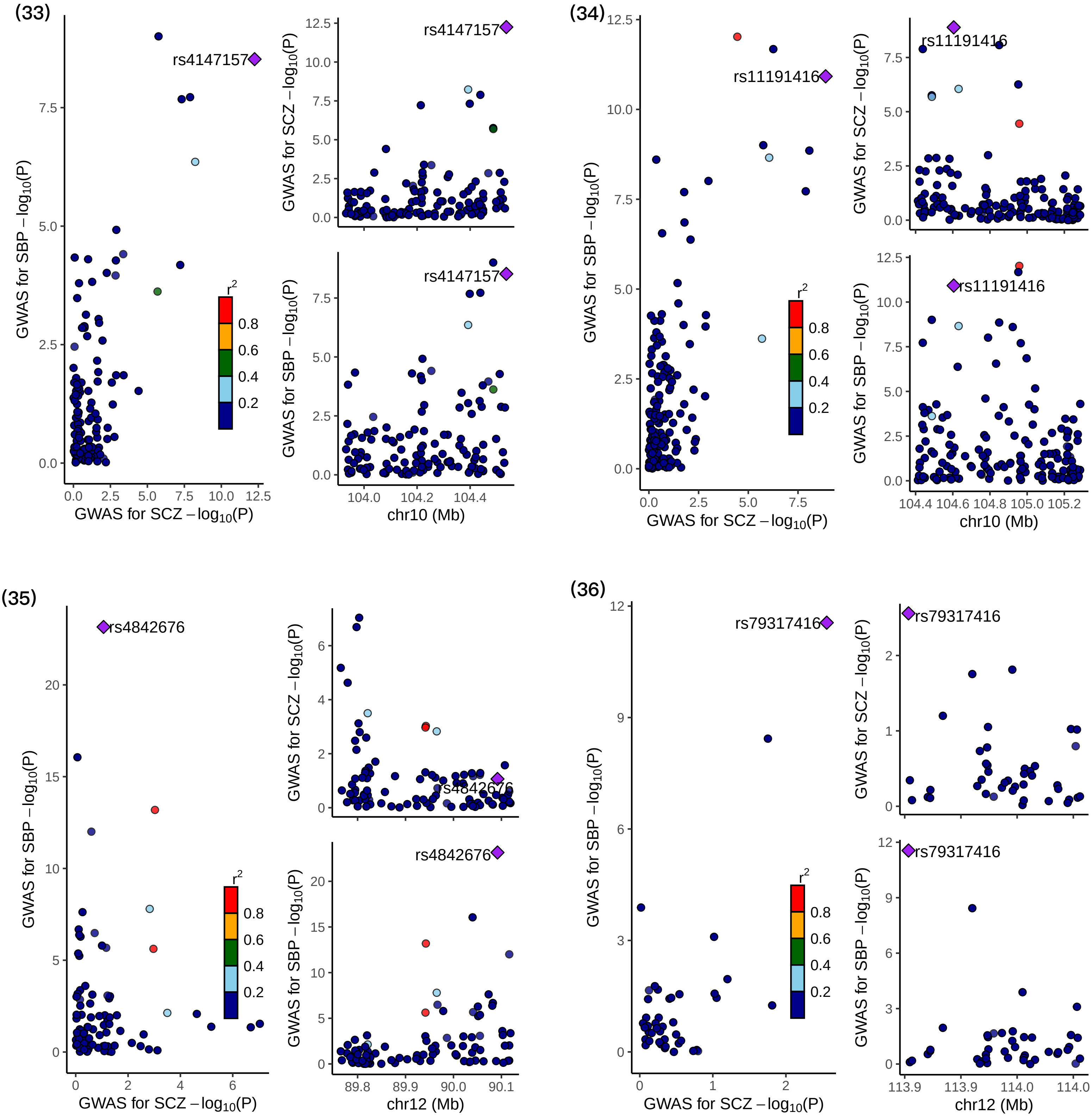

Supplemental Figure 5. Colocalization analysis for the pleiotropic loci

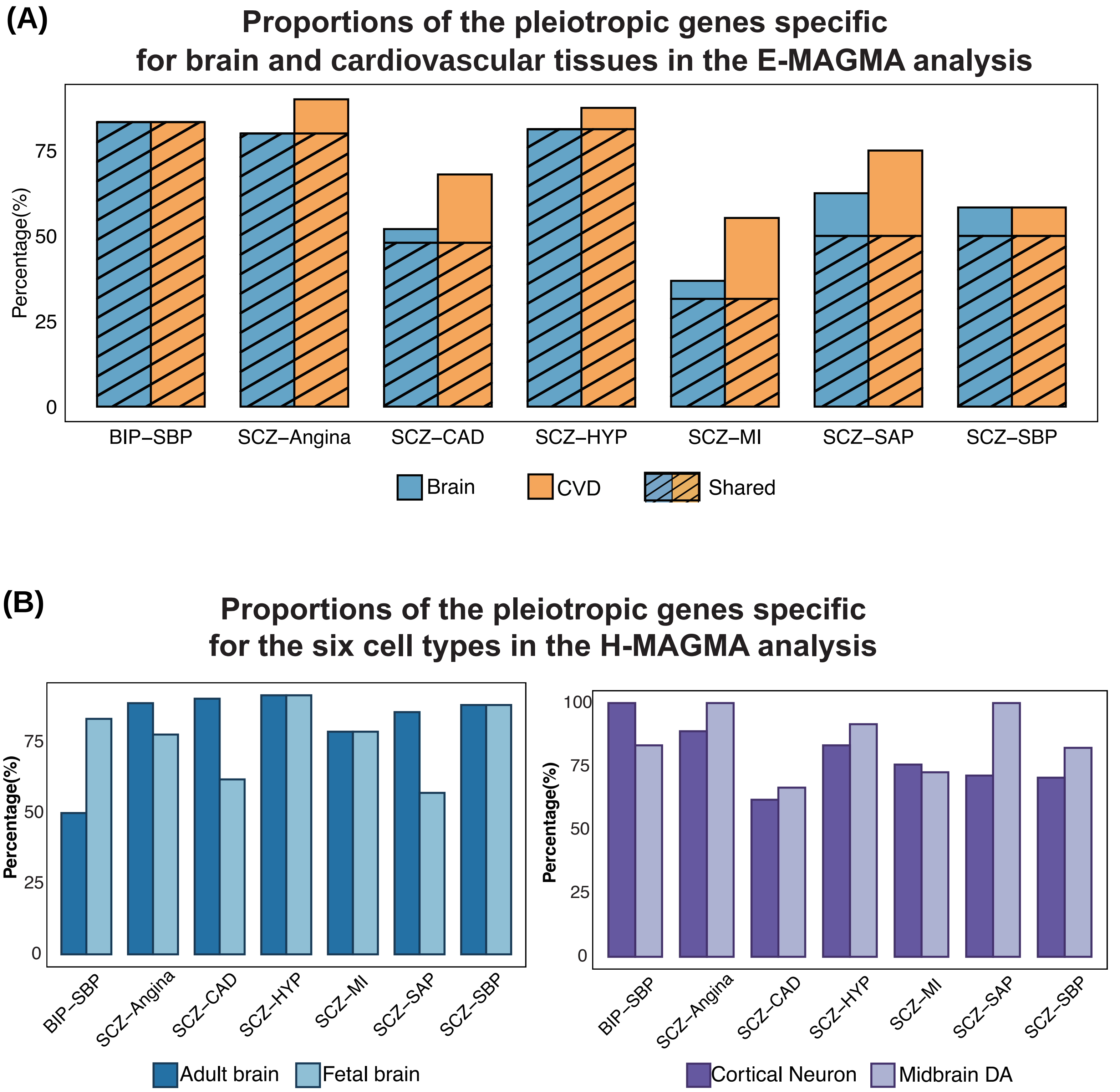

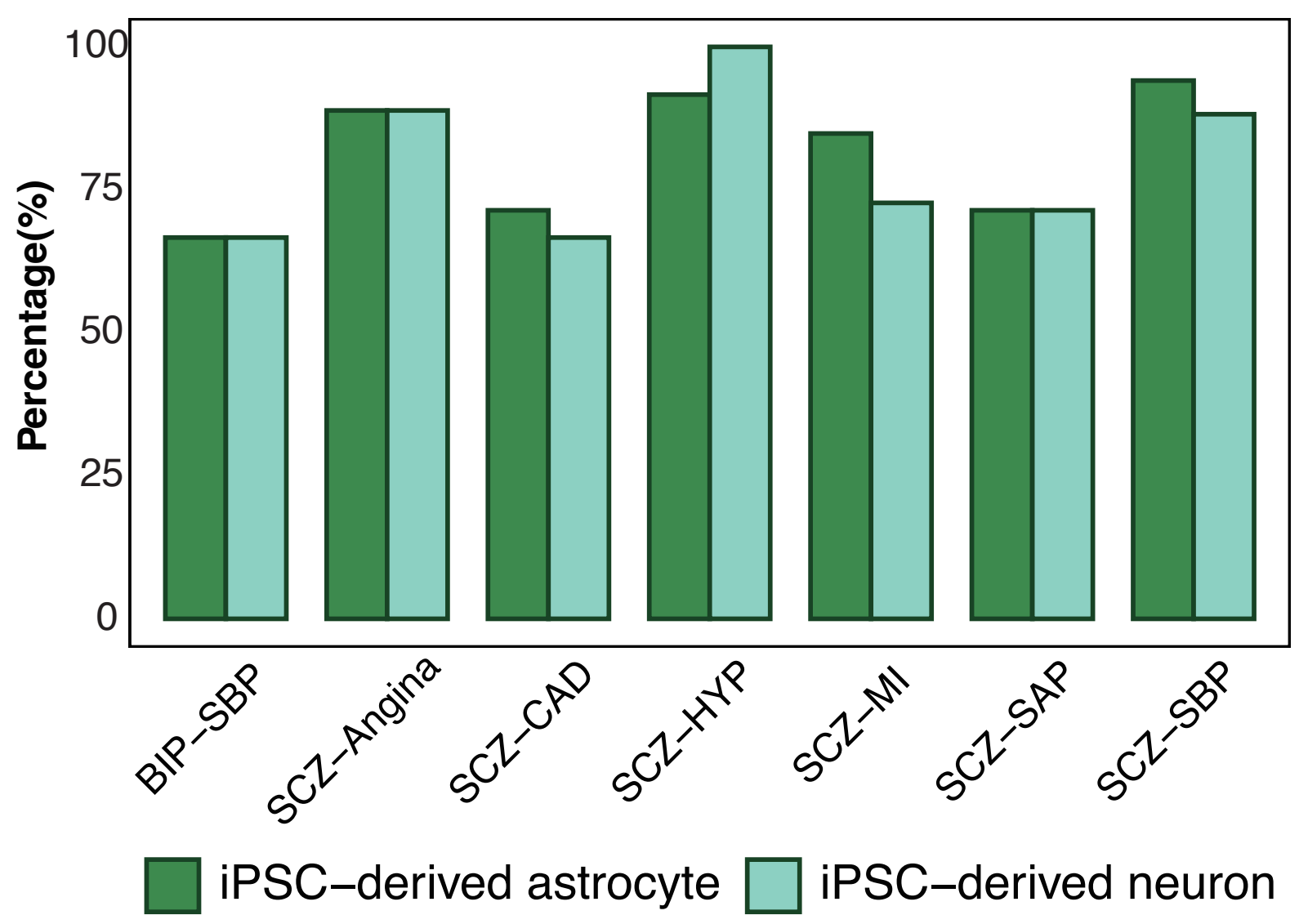

Supplemental Figure 6. Expression specificity for tissues and neurons in the E-MAGMA and H-MAGMA analyses

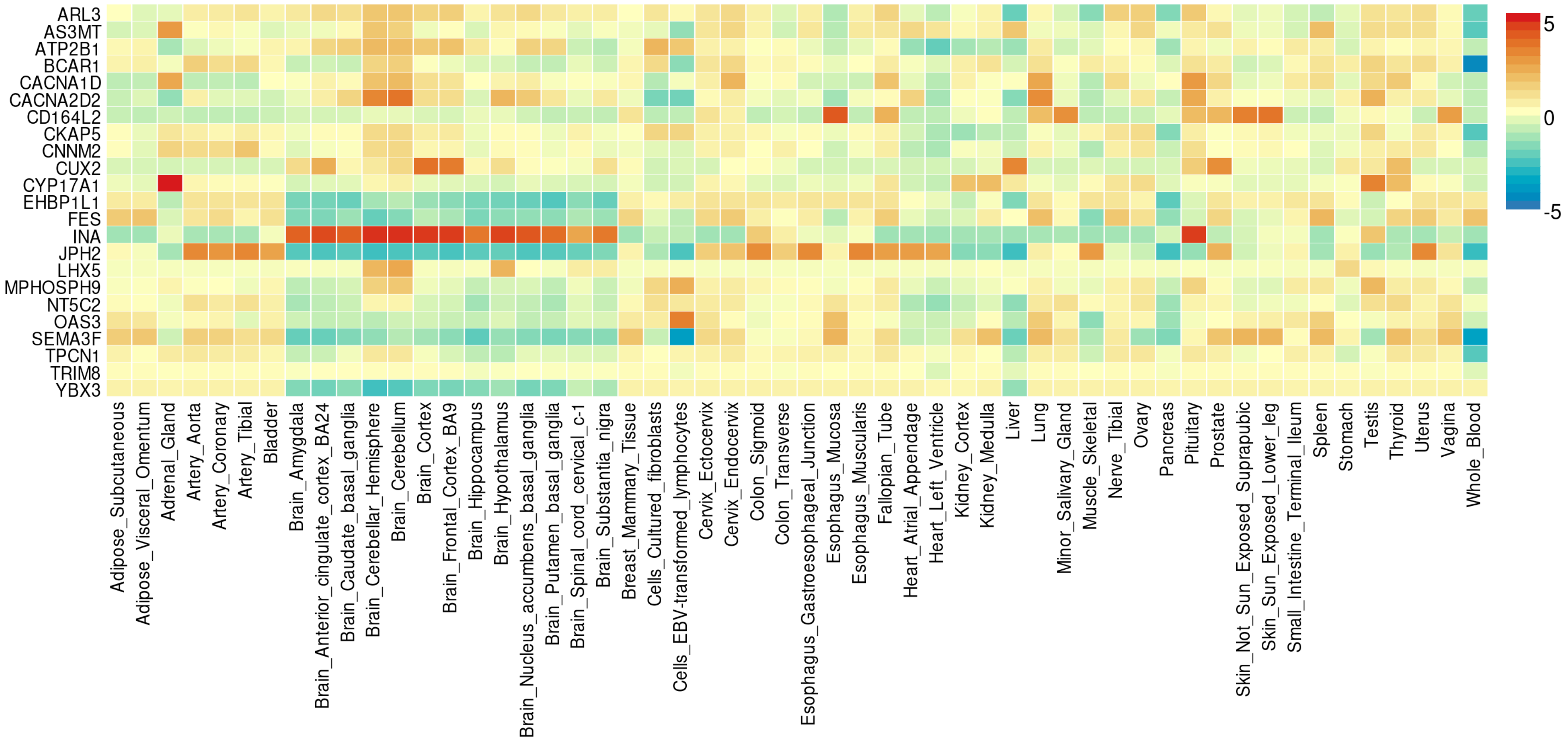

Supplemental Figure 7. Heatmap for the expression levels of the pleiotropic genes in the 54 tissues derived in FUMA platform

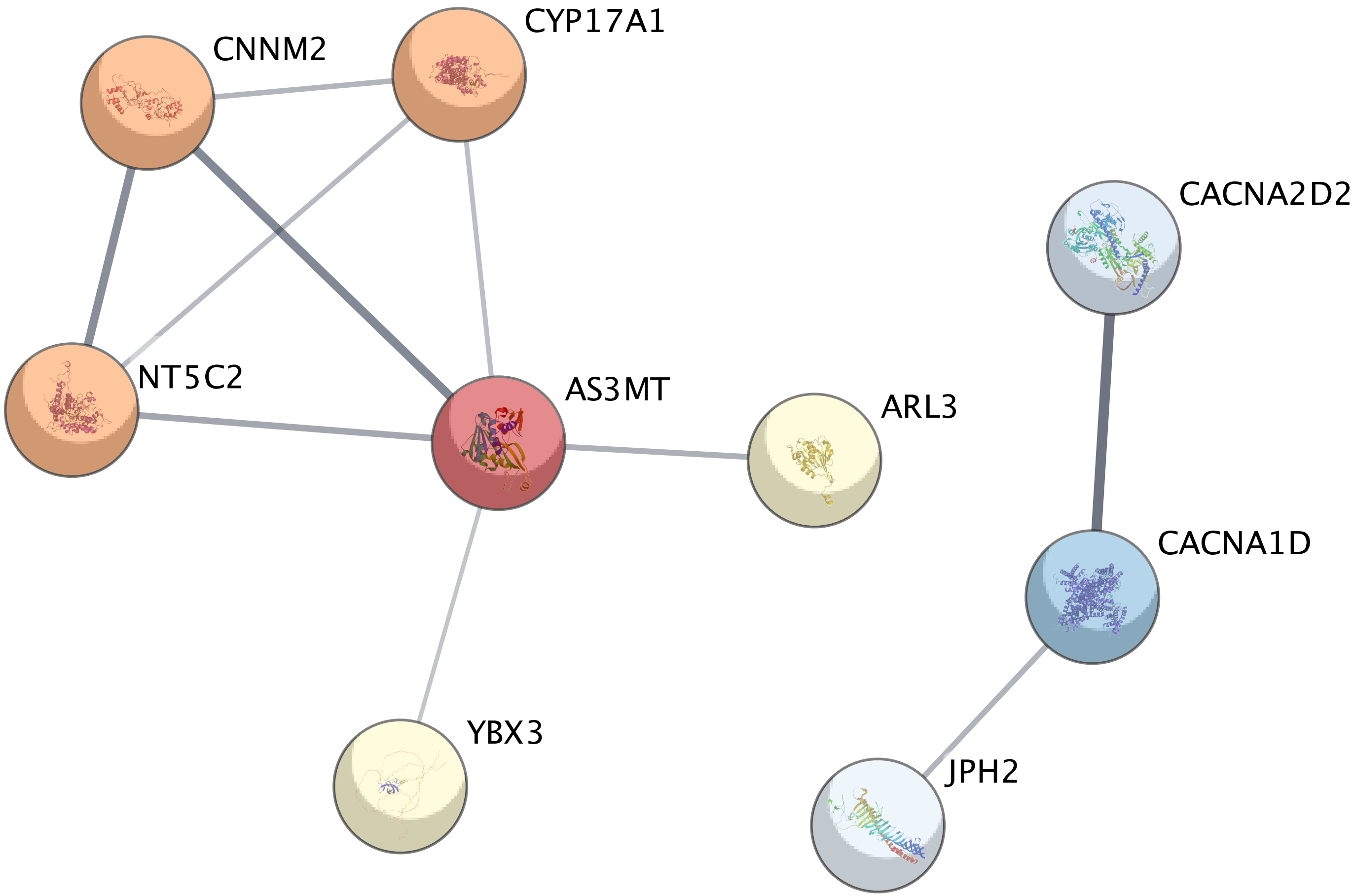

Supplementary Figure 8. Protein–protein interaction (PPI) network of pleiotropic genes identified from the STRING database

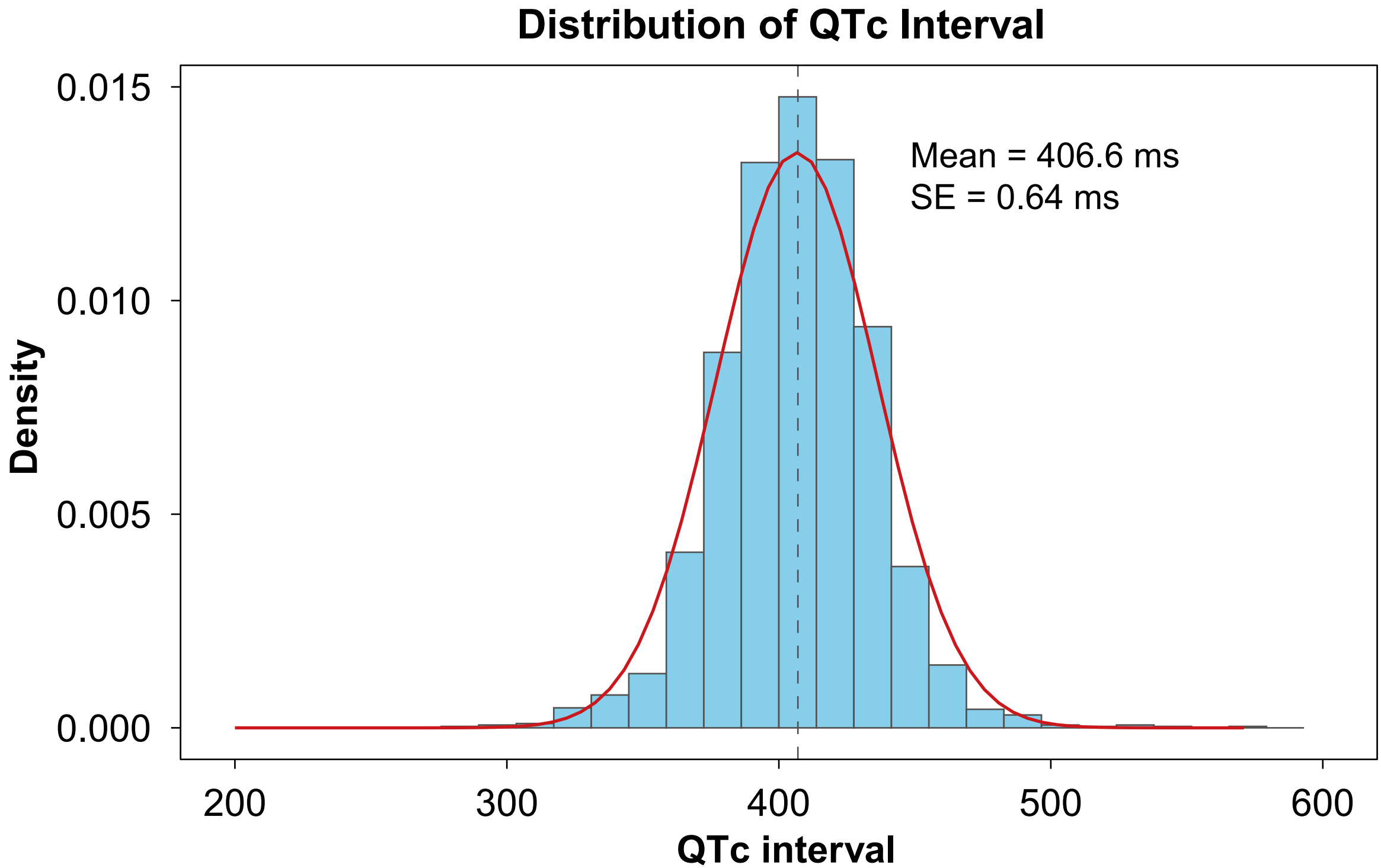

Supplemental Figure 9. Distribution of QTc interval among CAPOC participants
