# Supplementary material for: Calcium signaling pathway implicates a shared genetic basis between psychiatric and cardiovascular diseases
Source: Transl Psychiatry. 2026 Jul 11;16:527. doi: 10.1038/s41398-026-04222-z (PMC13642958; doi:10.1038/s41398-026-04222-z)
Supplement: Supplementary file 2 [file 41398_2026_4222_MOESM1_ESM.docx]

**Supplemental Methods**

**GWAS and eQTL summary statistics**

Given that cardiovascular comorbidity represents a substantial long-term health burden in adults with psychiatric disorders^1^, our study focused on major adult psychiatric disorders. Considering the currently publicly available East Asian GWAS summary statistics, we ultimately included schizophrenia (SCZ), major depressive disorder (MDD), and bipolar disorder (BIP). The genome-wide association studies (GWAS) summary statistics for psychiatric disorders were obtained from the Psychiatric Genomics Consortium in East Asians (PGC-EAS, https://pgc.unc.edu/for-researchers/download-results/) (14,004 SCZ cases, 16,757 controls; 15,771 MDD cases, 178,777 controls)^2,3^, and the GWAS of BIP among Chinese Han participants (1,822 cases, 4,650 controls)^4^.

The GWAS summary statistics of diverse cardiovascular diseases (CVDs) were obtained from the Biobank of Japan study (BBJ, https://pheweb.jp/)^5,6^, including angina (14,007 cases, 145,158 controls), coronary artery disease (CAD, 32,512 cases, 146,214 controls), chronic heart failure (CHF, 10,540 cases, 168,186 controls) , IS (22,664 cases, 152,022 controls), myocardial infarction (MI, 14,992 cases, 146,214 controls), unstable angina pectoris (UAP, 5,891 cases, 146,214 control), stable angina pectoris (SAP, 18,833 cases, 146,214 controls), systolic blood pressure (SBP, N=145,505) and diastolic blood pressure (DBP, N=145,515). Besides, GWAS for hypertension was obtained from the National Biobank of Korea (KoGES, https://koges.leelabsg.org/, 14,834 cases, 57,395 controls)^7^. The GWAS populations across the two types of diseases were independent (Supplemental Table 1).

In the two-sample Mendelian randomization (MR), we leveraged the expression quantitative trait loci (eQTL) data and GWAS summary statistics of psychiatric disorders (SCZ, BIP, and MDD), both in East Asian and European populations. Among East Asians, blood eQTL data from the Human Genetic Variation Database (N=298) were applied^8^. GWASs of psychiatric disorders were from PGC-East Asians^2,3^ and BIP GWAS among Chinese^4^. In Europeans, brain and cardiovascular tissues eQTL data were obtained from Genotype-Tissue Expression version 8 (GTEx V8, N=114~670)^9^ and eQTLGen consortium (N=31,684)^10^. Psychiatric disorders GWASs were obtained from the PGC-Europeans (74,776 SCZ cases, 101,023 controls; 41,917 BIP cases, 371,549 controls; 170,756 MDD cases, 329,443 controls)^2,11,12^ (Supplemental Tables 1-2).

To test whether the associations between drug target gene expressions and psychiatric disorders were independent of the blood pressure-lowering effect, we performed the MR analysis between blood pressure and psychiatric disorders in East Asians and Europeans. The GWAS summary statistics for blood pressures of East Asians were obtained from the BBJ study, and those of Europeans were obtained from a large-scale meta-analysis (N=1,028,980)^13^. The GWAS populations for exposures and outcomes were independent (Supplemental Tables 1-2).

Considering the complex linkage distribution (LD) structure in the major histocompatibility complex (MHC) region, we excluded the single nucleotide polymorphisms (SNPs) in this region (chromosome 6: 25-35 Mb). SNPs with minor allele frequency (MAF) <0.01 were also excluded. The present genomic data were aligned to hg19 human reference genomes, and the 1000 Genomes Phase III-East Asian (1000G EAS) ^14^ was used as a reference.

All procedures contributing to the present study comply with the ethical standards of the relevant national and institutional committees on human experimentation and the Helsinki Declaration of 1975, as revised in 2008. All study protocols were approved by the institutional ethics review boards at each site. Written or verbal informed consent was obtained from all participants.

**Chinese Antipsychotics Pharmacogenomics Consortium study**

**Study design and participants**

For the pathway-specific polygenic risk score (PRS) analysis, we applied the baseline phenotype and genotype data of the Chinese Han participants from the Chinese Antipsychotics Pharmacogenomics Consortium study (CAPOC), conducted in 2010 based on five research centers in China^15^, which was the largest antipsychotic pharmacogenomics study.

The inclusive criteria were mainly participants with diagnosed SCZ based on the Diagnostic and Statistical Manual of Mental Disorders (fourth edition, DSM-IV), aged 18~45 years, were of Chinese Han ancestry, were physically healthy with all laboratory measurements within the normal limitations, and were able to provide informed consent. Those who had severe, unstable physical diseases, such as hypertension, myocardial infarction, or other cardiac diseases; those who had QTc prolongation, or a history of congenital QTc prolongation were excluded. The well-organized inclusive and exclusive criteria can be found in our published works^15^. A total of 3030 participants were enrolled in the CAPOC study. The sociodemographic and clinical questionnaire data, physical measurements, and blood samples were collected upon the baseline assessment.

The present study additionally excluded participants without qualified genotype data (N=474), without complete baseline information for the current analysis (212 participants without course of disease information, 56 participants without body mass index information, 114 participants without QTc interval information, two participants without previous medication information), leaving a total of 2172 participants for the PRS analysis (Supplemental Figure 1). A comparison for the baseline characteristics between the included and excluded samples from CAPOC study was performed. The group difference was tested using linear/logistic regression models between inclusion/exclusion group and baseline characteristics, with adjusting for age, age^2^, sex, research centers.

The CAPOC study was approved by the ethics committee of the Peking University Sixth Hospital and each participating site. The reference number was 2009-LUNSHEN-23 and the approval date was April 21st, 2009. This study was registered at the Chinese Clinical Trial Registry (ChiCTR-TRC-10000934). Informed written consent was obtained from all included participants in accordance with the Declaration of Helsinki. All participants were asked to appoint a family member or close friend involved in the informed consent discussion and help the patients with decision-making.

**QTc interval**

The twelve-lead electrocardiography was applied for the QT interval measurement during the baseline assessment. Considering the clinical relevance, the QT interval was corrected for the heart rate based on Fridericia’s formula, recommended by the International Council for Harmonization of Technical Requirements for Pharmaceuticals for Human Use (ICH)^16,17^.

**Genotyping and quality control**

Procedures for genotyping, imputation, and quality control have been described previously^15,17^. Briefly, 900,015 SNPs were genotyped using the Illumina Human Omni ZhongHua­8 Beadchips (Illumina, San Diego, CA, USA) designed for the Chinese. The genomic quality control was performed and flipped out 189,892 SNPs with the following exclusive criteria: (1) duplicated SNPs, (2) call rate < 0.05, (3) MAF < 0.05, (4) Hardy-Weinberg P (P_HW_) < 10^-6^. Among the 3030 participants, 474 participants without qualified genotype data were excluded before the imputation following the exclusive criteria: (1) call rate < 0.05, (2) sex discrepancy; (3) relatedness (proportion identical-by-descent <0.2); (4) heterozygosity (> three standard deviations); (5) principal components outliers. Qualified samples and genotypes were phased using SHAPEIT (Version 2). The imputation was performed using IMPUTE (Version 2), taking the 1000G EAS^14^ as the reference. 11,986,772 SNPs were left with imputation quality scores > 0.6.

The present study additionally performed the genomic quality control for the imputed SNPs. The SNPs that met any of the criteria were excluded: (1) call rate < 0.02, (2) MAF < 0.01, (3) P_HW_ < 10^-6^. A total of 4,995,111 SNPs and 2,172 participants were included in the PRS analysis (Supplemental Figure 1).

**Genetic analysis incorporating pleiotropy and annotation (GPA)**

The GPA was applied to estimate the overall genetic overlaps between three major psychiatric disorders and ten cardiovascular traits. GPA assumes that p-values of SNPs associated with the trait follow a uniform distribution, whereas those trait-associated SNPs follow a Beta distribution. This assumption is then applied to the two GWASs and the SNPs are classified into four categories referred to the following models: (i) M_00_: SNPs not associated with both diseases; (ii) M_10_: SNPs only associated with the first disease; (iii) M_01_: SNPS only associated with the second disease; (iv) M_11_: SNPs associated with both diseases. GPA estimates proportion of SNPs in each model (PM). The likelihood ratio test is used to test the significance of overall genetic overlap. The pleiotropy association ratio (PAR) was calculated as PM_11_/(PM_10_ + PM_01_ + PM_11_) to evaluate the proportion of pleiotropic SNPs associated with both traits against proportion of SNPs associated with at least one trait. LD pruning was performed before the GPA analysis using PLINK 1.9 (window size=200kb, r^2^=0.2), to condition on the influence of LD^18,19^. The R package “GPA” (version 1.14.0) was applied for the current analysis.

**Pleiotropic analysis under the composite null hypothesis (PLACO)**

Extracting the overlapping loci from the two independent diseases GWASs missed the variants with pleiotropic effect but did not reach the genome-wide significance level in the two disease GWASs^20^. Based on the GWAS summary statistics of the two tested diseases, PLACO analysis^21^ tested against the null hypothesis assuming that the product of Z statistics for the same SNP on two tested diseases was not different from zero (Z_SNP→disease1_×Z_SNP→disease2_ = 0). The Z statistics were equal to the ratio of the effect size of the SNP on the single disease to the corresponding standard error (β_SNP→disease1_/SE_SNP→disease1_). We accounted for the correlation between the two testing diseases, estimated by the correlation matrix derived from the GWAS summary statistics of the two single diseases. We excluded SNPs with squared Z-scores > 80 considering the disturbing signals. P-value < 5×10^-8^ was set as the significant threshold for pleiotropic SNP identification. The positive and negative values of the T statistics indicate the concordant and discordant effect directions for the testing SNPs on the risk of two diseases. The R package “PLACO” (version 0.1.1) was applied for the current analysis.

Based on the 1000G EAS^14^, dependent significant SNPs at r^2^ > 0.1 or closer than 250kb were assigned to the same pleiotropic locus. The SNP with the minimum P-value within each pleiotropic locus was defined as the top SNP.

**Pleiotropic genes mapping and colocalization analysis**

Genes nearest to the pleiotropic loci or within a 10kb window around the pleiotropic loci were mapped as the pleiotropic genes using the “annotation” command in PLINK 1.9. The Single Nucleotide Polymorphism Database (dbSNP) build 129 was applied as the functional SNP attributes (https://www.cog-genomics.org/plink/1.9/resources#attrib), and the gene range list was based on the glist-hg19 (https://www.cog-genomics.org/plink/1.9/resources#genelist). The dbSNP platform (https://www.ncbi.nlm.nih.gov/snp/)^22^ was applied as a complementary approach for gene mapping. We also applied the SNP Nexus platform (https://www.snp-nexus.org/v4/)^23^ to annotate the cytogenetic location (band information) for the pleiotropic loci. Functional annotation for the top SNPs was performed using ANNOVAR software^24^. The amino acid change information was obtained from RefSeq Gene and UCSC Known Gene.

Furthermore, a Bayesian colocalization analysis was performed to pinpoint the possible causal SNP in each pleiotropic locus. The colocalization analysis tested the posterior probability of five hypotheses: H_0_: SNP was not associated with either disease. H_1_: SNP was only associated with the first disease. H_2_: SNP was only associated with the second trait; H_3_: Two independent SNPs were separately associated with the two diseases; H_4_: A shared SNP was associated with both diseases. The LD-pruning was performed before the Bayesian test using PLINK 1.9 (window size=500kb, r^2^=0.8). The posterior probability of H4 (PP.H4) larger than 0.6 was applied as the primary threshold to support causal SNPs^25^, and the SNP with the highest PP.H4 was reported as the causal variant within each pleiotropic locus^26,27^. A sensitivity analysis using a more stringent threshold of PP.H4 > 0.8 was also performed^28^.

**Tissue and cell-type specificity analysis**

The eQTL-informed MAGMA (E-MAGMA) and Hi-C–coupled MAGMA (H-MAGMA) were applied to indicate the tissue and cell-type specificities of the pleiotropic loci, respectively^29^. Based on the genome-wide pleiotropic results derived in the PLACO analysis, a gene-based analysis was performed to derive the pleiotropic genes, which were used as the input in the E-MAGMA and H-MAGMA analyses. Bonferroni correction for multiple testing of pleiotropic loci was applied. The expression levels of genes in 13 brain tissues and six cardiovascular tissues were derived from the GTEx V8, which were downloaded from the E-MAGMA GitHub website (https://github.com/eskederks/eMAGMA-tutorial) (Supplemental Table 13). The proportions of genes specifically expressed in brain tissues, cardiovascular tissues, and both types of tissues among all the input pleiotropic genes identified in the gene-based analysis were calculated. We tested the relationship between each of the prioritized genes and tissues using the functional mapping and annotation (FUMA) platform - GENE2FUNC function (https://fuma.ctglab.nl/gene2func)^30^. The gene expression heatmap showed the average of normalized expression per tissue, which allowed the comparison of the expression levels across different tissues within a gene.

H-MAGMA was applied to investigate the cell-type specificity^31^. Hi-C annotations of six types of neurons (fetal brain, adult brain, iPSC-derived neuron, iPSC-derived astrocyte, cortical neuron, adult midbrain dopaminergic) were included, which were downloaded from H-MAGMA GitHub website (https://github.com/thewonlab/H-MAGMA/tree/master/Input_Files) (Supplemental Table 16). The proportions of genes specifically expressed in the specific neurons among all the input pleiotropic genes identified in the gene-based analysis were calculated. Bonferroni correction for multiple testing of pleiotropic loci was applied.

**Enrichment and protein–protein interaction analyses**

The gene-set enrichment analysis for the pleiotropic genes identified in the annotation analysis was performed using the FUMA - GENE2FUNC function (https://fuma.ctglab.nl/gene2func)^30^. The Gene Ontology (GO) gene sets were obtained from the Molecular Signatures Database (MSigDB)^32^. The phenotypic gene sets were derived from the GWAS catalog. FDR was applied for multiple testing correction. Protein–protein interaction (PPI) analysis was performed using the STRING database (version 12.0, https://string-db.org/), which characterizes known and predicted functional associations among proteins^33^. Each interaction is assigned a confidence score ranging from 0 to 1, reflecting the probability that the association is biologically meaningful rather than the strength or specificity of the interaction itself. The combined score is calculated by integrating evidence from multiple channels, including genomic context, co-expression, experiments, curated databases, and text mining, while correcting for the probability of random co-occurrence. The resulting PPI network was visualized in STRING, with nodes representing proteins and edges representing protein associations; edge thickness was proportional to the combined confidence score. Node degree, defined as the number of direct connections for each protein, was used to indicate its relative connectivity within the network.

**Construction of whole-genome PRS and pathway-specific of SCZ**

We constructed a whole-genome PRS of SCZ using the PRS-CS in the CAPOC study, based on the GWAS summary statistics of SCZ from East Asians in PGC (14,004 SCZ cases, 16,757 controls)^2^. The PRS-CS is a Bayesian approach that applies continuous shrinkage priors on SNP effect sizes and therefore eliminates the needs to specify a GWAS P-value threshold for SNP selection^34^.

The PRSet was applied to calculate the gene-set-based PRSs for SCZ. The GWAS of SCZ from East Asians in PGC^2^ was applied as the base dataset. The genetic pathways related to calcium were selected from GO in the MSigDB^32^. A P-value threshold of 1.00 was used for PRSet considering a small proportion of SNPs in each gene set. We applied both the self-contained and competitive P values as the criteria for testing the significance of each gene set. The self-contained P values tested the association with SCZ, whereas the competitive P values tested the enrichment of specific gene sets. The competitive P value was derived by comparing the observed gene-set PRS association against the 10 000 permuted null P value distribution of random gene-set polygenic risk scores. The calcium PRSs associated with the genetic predisposition to SCZ were included for further analysis (self-contained P value < 0.05, competitive P value < 0.05). The original PRS was transformed into the standard normal distribution.

We described the baseline characteristics of participants in different calcium pathway–specific PRS (GO:0007223) tertiles of SCZ, which meant the low, intermediate, and high genetic risk of SCZ mediated by the specific calcium pathway. The linear trend was tested by regressions between the PRS and the baseline characteristics, in which the PRS was treated as a continuous variable, adjusting for age, age^2^, sex, research centers, and the top five genetic principal components.

Linear regression was used to estimate the association between whole-genome PRS of SCZ, and each of the calcium pathway–specific PRSs on the QTc interval at baseline. Model 1 estimated the crude associations between PRS and the QTc interval. To account for population stratification, age, age^2^, sex, and research centers, the top five genetic principal components were applied as covariates in Model 2. Body mass index (kg/m^2^) and course of disease (years) were additionally adjusted in Model 3 to explore whether the effect of the pathway-specific shared genetics could be confounded by these non-genetic exposures^35^.

**Stratified analysis**

Considering that the medication history might influence the QT interval, we performed a sensitivity analysis excluding participants previously prescribed anti-CVD drugs (N=3, one participant with atenolol medication, one participant with isosorbide dinitrate medication, one participant with dioscorea panthaica prain et burkill medication), or antipsychotics with moderate to high effects on QT interval (N=89, 27 participants with chlorpromazine medication, 58 participants with quetiapine medication, four participants with ziprasidone)^36^. In addition, given that the clinical profiles of SCZ and CVD are likely to differ across age groups, we performed age-stratified analyses in a younger group (<30 years, N = 1043) and an older group (≥30 years, N = 1129).

**MR analysis for anti-CVD drug target gene expressions on psychiatric disorders**

We performed a two-sample MR between the expression levels of anti-CVD drug target genes in the brain and cardiovascular tissues and psychiatric disorders among East Asians and Europeans. Besides, to estimate whether the effect of anti-CVD drugs on psychiatric disorders was independent of blood pressure, we performed a two-sample MR analysis between blood pressure and the three psychiatric disorders in the two ancestries.

**Instrument selection**

Referring to the World Health Organization Collaborating Centre for Drug Statistics Methodology, we selected six classes of anti-CVD drugs (C01: Cardiac therapy, C03: Diuretics, C04: Peripheral vasodilators, C07: Beta blocking agents, C08: Calcium channel blockers, C09: Agents acting on the renin-angiotensin system), which were classified by the Anatomical Therapeutic Chemical classification system. We listed the specific types of drugs within each drug class in Supplemental Table 3. The target genes for each drug were defined using the DrugBank platform (https://go.drugbank.com/)^37^. Only the target genes with clear pharmacological actions (inhibitor or agonist) were included. Searching on the GWAS catalog (https://www.ebi.ac.uk/gwas/), we excluded the drug target genes previously reported to be associated with psychiatric disorders among the East Asian and European populations (P < 5 × 10^−8^) (*ADRA1D*, *KCNA5*, *KCND2*, *KCND3*, *KCNJ3*, *SCN9A*, *SLC8A1* for cardiac therapy agents; *ASIC1*, *CA12*, *KCNMA1*, *NR3C2*, *PDE4B* for diuretics; *ADRB2*, *HTR1B* for beta blocking agents; *ABCB1*, *CACNA1B*, *CACNA1C*, *CACNA1D*, *CACNA1G*, *CACNA1I*, *CACNA1S*, *CACNA2D1*, *CACNA2D2*, *CACNA2D3*, *CACNB2*, *CACNB3*, *CACNG1*, *CHRM3*, *CHRM5* for calcium channel blockers).

To minimize the potential horizontal pleiotropy due to the trans-effect^38^, we selected cis-variants within each encoding gene (±2,500 base pairs), and only included the SNPs associated with the expressions of the drug target genes (P < 1 × 10^−4^). The SNPs within each target gene region were then clumped (r^2^=0.001, window=10,000kb, referred to the 1000G EAS or EUR population^14^ as appropriate). The top SNPs were applied as the instrumental variables (IVs) for MR analysis. In total, SNPs in 13 target genes significantly expressed in whole blood among East Asians, and 67 target genes significantly expressed in the brain and cardiovascular tissues among the Europeans, were selected for analysis (Supplemental Tables 3,27).

For the blood pressure applied as the exposure, SNPs with P < 5×10^-8^ were clumped for independence (r^2^=0.001, window=10,000kb, referred to the 1000G EAS or EUR population^14^ as appropriate). Proxy SNPs (r^2^ > 0.8) were applied when the selected SNPs were not available in the outcome dataset. SNPs with MAF <0.01 were excluded. The LDtrait Tool (https://ldlink.nih.gov/?tab=ldtrait) was used^39^, and SNPs or the proxy SNPs (r^2^>0.8) strongly associated (P<1×10^-5^) with the outcomes were excluded. Palindromic SNPs with intermediate allele frequencies (MAF>0.4) were disregarded. MR-Pleiotropy RESidual Sum and Outlier (MR-PRESSO) test was applied, and the outlier SNPs were removed when the MR-PRESSO global test was significant^40^. The IVs selected to proxy blood pressure could be found in Supplemental Table 30.

To test the weak IVs bias, the present study calculated the *F* statistics for each SNP (*F* = (*β* / SE)^2^) and the average *F* statistics for all the IVs for the same exposure (*F* = R^2^ (n - k - 1) / k (1 - R^2^)). *β* meant the effect of each SNP on the exposure, and SE meant the standard error of *β*. R^2^ was the variance of exposure explained by IVs, obtained from the MR Steiger direction test, n meant the sample size, and k meant the number of IVs. *F* statistic greater than ten meant the possibility of the weak IVs bias was small^41^.

**Mendelian randomization analysis**

The Wald ratio test was applied in the causal association estimated with one single IV. For the causal estimation with multiple IVs, inverse variance weighted (IVW)^42^ was applied as the primary analysis, along with the weighted median (WM)^43^, MR-Egger (ME)^44^, and MR-robust adjusted profile score (RAPS)^45^ methods as sensitivity analyses. IVW method provided an unbiased causal estimate if there was no horizontal pleiotropy. WM gave valid tests even if the prevalence of invalid SNPs was up to 50%^43^. Test for intercepts of ME regression was used to indicate the degree of directional horizontal pleiotropy. RAPS method was robust to the systematic and idiosyncratic pleiotropy and could give a robust estimate for MR analysis with weak IVs, and was recommended when the exposure and outcome were complex traits. Cochrane’s *Q* test was performed to assess the heterogeneity among SNPs, and a random-effect model in IVW was applied if heterogeneity existed (P<0.05). Steiger directionality test was applied to test the causal direction between the hypothesized exposure and outcomes^46^.

Analyses were carried out using R packages “TwoSampleMR” (version 0.5.6)^47^, “MR.RAPS” (version 0.2)^45^, “MR-PRESSO” (version 1.0)^40^, and “MendelianRandomization” (version 0.6.0) in the R environment (version 4.1.3).

**Supplemental Figure Legends**

**Supplemental Figure 1. Quality control for the sample and variants in the Chinese Antipsychotics Pharmacogenomics Consortium study.**

Abbreviations: QC, quality control. CAPOC, Chinese Antipsychotics Pharmacogenomics Consortium; PRS, polygenic risk score; MAF, minor allele frequency; 1000 Genomes Phase III-East Asian (1000G EAS).

**Supplemental Figure 2. Manhattan plots for the genome-wide pleiotropic analysis.**

Abbreviations: SCZ, schizophrenia, BIP, bipolar disorder; HYP, hypertension; MI, myocardial infarction; SAP, stable angina pectoris; SBP, systolic blood pressure.

Notes: For the Manhattan plots, the X-axis denoted the genomic position (chromosomes 1-22), Y-axis denoted the log_10_(P-value) of the association test. Genome-wide significance level (P=5×10^-8^) was denoted by the dashed line.

**Supplemental Figure 3. QQ plots for the genome-wide pleiotropic analysis.**

Abbreviations: SCZ, schizophrenia, BIP, bipolar disorder; HYP, hypertension; MI, myocardial infarction; SAP, stable angina pectoris; SBP, systolic blood pressure.

Notes: The lambda values were shown in the bottom right corners.

**Supplemental Figure 4. Comparison of** **minor allele frequencies of the pleiotropic loci between East Asian and European populations.**

Abbreviations: MAF, minor allele frequency; EAS, East Asian; EUR, European.

Notes: The novel loci (Panel A) and known loci (Panel B) were separately compared for the MAFs between East Asians and Europeans based on the 1000 Genomes Phase III. The Mann-Whitney U test was applied for comparison. The P values were presented above each bar plot. The corresponding values of MAFs are shown in Supplemental Table 8.

**Supplemental Figure 5. Colocalization analysis for the pleiotropic loci.**

Abbreviations: SCZ, schizophrenia, BIP, bipolar disorder; HYP, hypertension; MI, myocardial infarction; SAP, stable angina pectoris; SBP, systolic blood pressure.

Notes: Each panel showed a colocalized locus (PP.H4>0.6) identified for the corresponding disease pair. The associations for each disease were shown on the right, and the combined association for pairwise disease was shown on the left. The candidate-shared causal variant identified by pairwise colocalization analysis was shown in purple diamond. The color of each variant represented its linkage distribution (LD) relationship with the candidate-shared causal variant. All genomic location is based on reference genome hg19, and LD calculation was based on 1000 Genomes in Phase III of the East Asian population.

(1) 20q13.12 for BIP-SBP; (2) 10q24.32 for BIP-SBP; (3) 6p21.1 for BIP-SBP; (4) 10q24.32 for SCZ-Angina; (5) 10q24.33 for SCZ-Angina; (6) 11q13.1 for SCZ-Angina; (7) 10q24.32 for SCZ-CAD; (8) 10q24.33 for SCZ-CAD; (9) 11p11.2 for SCZ-CAD; (10) 11q13.1 for SCZ-CAD; (11) 12p13.2 for SCZ-CAD; (12) 12q24.13 for SCZ-CAD; (13) 12q24.31 for SCZ-CAD; (14) 15q25.1 for SCZ-CAD; (15) 15q26.1 for SCZ-CAD; (16) 3p21.1 for SCZ-HYP; (17) 4q21.21 for SCZ-HYP; (18) 10q24.32 for SCZ-HYP; (19) 10q24.33 for SCZ-HYP; (20) 12q21.33 for SCZ-HYP; (21) 12q24.13 for SCZ-HYP; (22) 10q24.32 for SCZ-MI; (23) 10q24.33 for SCZ-MI; (24) 11p11.2 for SCZ-MI; (25) 12q24.11 for SCZ-MI; (26) 12q24.13 for SCZ-MI; (27) 12q24.31 for SCZ-MI; (28) 10q24.32 for SCZ-SAP; (29) 10q24.33 for SCZ-SAP; (30) 11q13.1 for SCZ-SAP; (31) 12q24.13 for SCZ-SAP; (32) 15q26.1 for SCZ-SAP; (33) 10q24.32 for SCZ-SBP; (34) 10q24.33 for SCZ-SBP; (35) 12q21.33 for SCZ-SBP; (36) 12q24.13 for SCZ-SBP. The corresponding results are shown in Supplemental Table 10.

**Supplemental Figure 6. Expressive specificities for tissues and neurons in the E-MAGMA and H-MAGMA analyses.**

Abbreviations: E-MAGMA, eQTL-informed MAGMA; H-MAGMA, Hi-C–coupled MAGMA; SCZ, schizophrenia; BIP, bipolar disorder; CAD, coronary artery disease; HYP, hypertension; MI, myocardial infarction; SAP, stable angina pectoris; UAP, unstable angina pectoris; SBP, systolic blood pressure.

Notes: The proportions of the pleiotropic genes specifically expressed in the tissue and cell types among all the pleiotropic genes derived in the E-MAGMA (Panel A) and H-MAGMA (Panel B) analyses were shown in the histogram. Besides, proportions of pleiotropic genes showed expressive specificities in both brain and cardiovascular tissues were presented in a shadowed histogram. The corresponding results are shown in Supplemental Tables 13-19.

**Supplemental Figure 7. Heatmap for the expression levels of the pleiotropic genes in the 54 tissues derived from the FUMA platform.**

Notes: The gene expression heatmap showed the average of normalized expression per tissue (zero mean across samples) from Genotype-Tissue Expression version 8, which allowed the comparison of gene expression across tissues within a gene. The corresponding results are shown in Supplemental Table 12.

**Supplemental Figure 8. Protein–protein interaction (PPI) network of** **pleiotropic genes identified from the STRING database**

Notes: Nodes represent proteins and edges represent protein–protein interactions. The network was grouped into two clusters and colored using two separate color schemes. Within each cluster, darker colors indicate higher node degree. Edge thickness is proportional to the STRING combined score. The corresponding results are shown in Supplemental Table 21-22.

**Supplemental Figure 9. Distribution of QTc interval among CAPOC participants**

Notes: CAPOC, Chinese Antipsychotics Pharmacogenomics Consortium. The mean and standard error (SE) values are displayed.

**References**

1.	Vaccarino V., Prescott E., Shah A.J., Bremner J.D., Raggi P., Dobiliene O. *et al.* Mental health disorders and their impact on cardiovascular health disparities. *Lancet Reg Health Eur* **56**, 101373 (2025).

2.	Trubetskoy V., Pardiñas A.F., Qi T., Panagiotaropoulou G., Awasthi S., Bigdeli T.B. *et al.* Mapping genomic loci implicates genes and synaptic biology in schizophrenia. *Nature* **604**, 502-508 (2022).

3.	Giannakopoulou O., Lin K., Meng X., Su M.H., Kuo P.H., Peterson R.E. *et al.* The Genetic Architecture of Depression in Individuals of East Asian Ancestry: A Genome-Wide Association Study. *JAMA Psychiatry* **78**, 1258-1269 (2021).

4.	Li H.J., Zhang C., Hui L., Zhou D.S., Li Y., Zhang C.Y. *et al.* Novel Risk Loci Associated With Genetic Risk for Bipolar Disorder Among Han Chinese Individuals: A Genome-Wide Association Study and Meta-analysis. *JAMA Psychiatry* **78**, 320-330 (2021).

5.	Sakaue S., Kanai M., Tanigawa Y., Karjalainen J., Kurki M., Koshiba S. *et al.* A cross-population atlas of genetic associations for 220 human phenotypes. *Nat Genet* **53**, 1415-1424 (2021).

6.	Masahiro Kanai J.C.U., Juha Karjalainen, Mitja Kurki, Konrad J Karczewski, Eric Fauman, Qingbo S Wang, Hannah Jacobs, François Aguet, Kristin G Ardlie, Nurlan Kerimov, Kaur Alasoo, Christian Benner, Kazuyoshi Ishigaki, Saori Sakaue, Steven Reilly, The BioBank Japan Project, FinnGen, Yoichiro Kamatani, Koichi Matsuda, Aarno Palotie, Benjamin M Neale, Ryan Tewhey, Pardis C Sabeti, Yukinori Okada, Mark J Daly, Hilary K Finucane. Insights from complex trait fine-mapping across diverse populations. (medRxiv, 2021).

7.	Nam K., Kim J. & Lee S. Genome-wide study on 72,298 individuals in Korean biobank data for 76 traits. *Cell Genom* **2**, 100189 (2022).

8.	Narahara M., Higasa K., Nakamura S., Tabara Y., Kawaguchi T., Ishii M. *et al.* Large-scale East-Asian eQTL mapping reveals novel candidate genes for LD mapping and the genomic landscape of transcriptional effects of sequence variants. *PLoS One* **9**, e100924 (2014).

9.	Consortium T.G., Aguet F., Anand S., Ardlie K.G., Gabriel S., Getz G.A. *et al.* The GTEx Consortium atlas of genetic regulatory effects across human tissues. *Science* **369**, 1318-1330 (2020).

10.	Võsa U., Claringbould A., Westra H.J., Bonder M.J., Deelen P., Zeng B. *et al.* Large-scale cis- and trans-eQTL analyses identify thousands of genetic loci and polygenic scores that regulate blood gene expression. *Nat Genet* **53**, 1300-1310 (2021).

11.	Mullins N., Forstner A.J., O'Connell K.S., Coombes B., Coleman J.R.I., Qiao Z. *et al.* Genome-wide association study of more than 40,000 bipolar disorder cases provides new insights into the underlying biology. *Nat Genet* **53**, 817-829 (2021).

12.	Howard D.M., Adams M.J., Clarke T.K., Hafferty J.D., Gibson J., Shirali M. *et al.* Genome-wide meta-analysis of depression identifies 102 independent variants and highlights the importance of the prefrontal brain regions. *Nat Neurosci* **22**, 343-352 (2019).

13.	Keaton J.M., Kamali Z., Xie T., Vaez A., Williams A., Goleva S.B. *et al.* Genome-wide analysis in over 1 million individuals of European ancestry yields improved polygenic risk scores for blood pressure traits. *Nat Genet* **56**, 778-791 (2024).

14.	Auton A., Abecasis G.R., Altshuler D.M., Durbin R.M., Abecasis G.R., Bentley D.R. *et al.* A global reference for human genetic variation. *Nature* **526**, 68-74 (2015).

15.	Yu H., Yan H., Wang L., Li J., Tan L., Deng W. *et al.* Five novel loci associated with antipsychotic treatment response in patients with schizophrenia: a genome-wide association study. *Lancet Psychiatry* **5**, 327-338 (2018).

16.	Funk M.C., Beach S.R., Bostwick J.R., Celano C., Hasnain M., Pandurangi A. *et al.* QTc Prolongation and Psychotropic Medications. *Am J Psychiatry* **177**, 273-274 (2020).

17.	Lu Z., Zhang Y., Yan H., Su Y., Guo L., Liao Y. *et al.* ATAD3B and SKIL polymorphisms associated with antipsychotic-induced QTc interval change in patients with schizophrenia: a genome-wide association study. *Transl Psychiatry* **12**, 56 (2022).

18.	Chung D., Yang C., Li C., Gelernter J. & Zhao H. GPA: a statistical approach to prioritizing GWAS results by integrating pleiotropy and annotation. *PLoS Genet* **10**, e1004787 (2014).

19.	Gong W., Guo P., Li Y., Liu L., Yan R., Liu S. *et al.* Role of the Gut-Brain Axis in the Shared Genetic Etiology Between Gastrointestinal Tract Diseases and Psychiatric Disorders: A Genome-Wide Pleiotropic Analysis. *JAMA Psychiatry* **80**, 360-370 (2023).

20.	Andreassen O.A., Djurovic S., Thompson W.K., Schork A.J., Kendler K.S., O'Donovan M.C. *et al.* Improved detection of common variants associated with schizophrenia by leveraging pleiotropy with cardiovascular-disease risk factors. *Am J Hum Genet* **92**, 197-209 (2013).

21.	Ray D. & Chatterjee N. A powerful method for pleiotropic analysis under composite null hypothesis identifies novel shared loci between Type 2 Diabetes and Prostate Cancer. *PLoS Genet* **16**, e1009218 (2020).

22.	Smigielski E.M., Sirotkin K., Ward M. & Sherry S.T. dbSNP: a database of single nucleotide polymorphisms. *Nucleic Acids Res* **28**, 352-5 (2000).

23.	Oscanoa J., Sivapalan L., Gadaleta E., Dayem Ullah A.Z., Lemoine Nicholas R. & Chelala C. SNPnexus: a web server for functional annotation of human genome sequence variation (2020 update). *Nucleic Acids Research* **48**, W185-W192 (2020).

24.	Wang K., Li M. & Hakonarson H. ANNOVAR: functional annotation of genetic variants from high-throughput sequencing data. *Nucleic Acids Res* **38**, e164 (2010).

25.	Han X., Gharahkhani P., Hamel A.R., Ong J.S., Rentería M.E., Mehta P. *et al.* Large-scale multitrait genome-wide association analyses identify hundreds of glaucoma risk loci. *Nat Genet* **55**, 1116-1125 (2023).

26.	Giambartolomei C., Vukcevic D., Schadt E.E., Franke L., Hingorani A.D., Wallace C. *et al.* Bayesian test for colocalisation between pairs of genetic association studies using summary statistics. *PLoS Genet* **10**, e1004383 (2014).

27.	Wallace C. A more accurate method for colocalisation analysis allowing for multiple causal variants. *PLoS Genet* **17**, e1009440 (2021).

28.	Roychowdhury T., Klarin D., Levin M.G., Spin J.M., Rhee Y.H., Deng A. *et al.* Genome-wide association meta-analysis identifies risk loci for abdominal aortic aneurysm and highlights PCSK9 as a therapeutic target. *Nat Genet* **55**, 1831-1842 (2023).

29.	Gerring Z.F., Mina-Vargas A., Gamazon E.R. & Derks E.M. E-MAGMA: an eQTL-informed method to identify risk genes using genome-wide association study summary statistics. *Bioinformatics* **37**, 2245-2249 (2021).

30.	Watanabe K., Taskesen E., van Bochoven A. & Posthuma D. Functional mapping and annotation of genetic associations with FUMA. *Nat Commun* **8**, 1826 (2017).

31.	Sey N.Y.A., Hu B., Mah W., Fauni H., McAfee J.C., Rajarajan P. *et al.* A computational tool (H-MAGMA) for improved prediction of brain-disorder risk genes by incorporating brain chromatin interaction profiles. *Nat Neurosci* **23**, 583-593 (2020).

32.	Liberzon A., Birger C., Thorvaldsdóttir H., Ghandi M., Mesirov J.P. & Tamayo P. The Molecular Signatures Database (MSigDB) hallmark gene set collection. *Cell Syst* **1**, 417-425 (2015).

33.	Szklarczyk D., Kirsch R., Koutrouli M., Nastou K., Mehryary F., Hachilif R. *et al.* The STRING database in 2023: protein-protein association networks and functional enrichment analyses for any sequenced genome of interest. *Nucleic Acids Res* **51**, D638-d646 (2023).

34.	Ge T., Chen C.Y., Ni Y., Feng Y.A. & Smoller J.W. Polygenic prediction via Bayesian regression and continuous shrinkage priors. *Nat Commun* **10**, 1776 (2019).

35.	Kappel D.B., Smart S.E., Owen M.J., O'Donovan M.C., Pardiñas A.F. & Walters J.T.R. Association between genetic liability to physical health conditions and comorbidities in individuals with severe mental illness: an analysis of two cross-sectional observational studies in the UK. *Lancet Psychiatry* **12**, 447-456 (2025).

36.	Lambiase P.D., de Bono J.P., Schilling R.J., Lowe M., Turley A., Slade A. *et al.* British Heart Rhythm Society Clinical Practice Guidelines on the Management of Patients Developing QT Prolongation on Antipsychotic Medication. *Arrhythm Electrophysiol Rev* **8**, 161-165 (2019).

37.	Wishart D.S., Feunang Y.D., Guo A.C., Lo E.J., Marcu A., Grant J.R. *et al.* DrugBank 5.0: a major update to the DrugBank database for 2018. *Nucleic Acids Res* **46**, D1074-d1082 (2018).

38.	Richardson T.G., Zheng J., Davey Smith G., Timpson N.J., Gaunt T.R., Relton C.L. *et al.* Mendelian Randomization Analysis Identifies CpG Sites as Putative Mediators for Genetic Influences on Cardiovascular Disease Risk. *Am J Hum Genet* **101**, 590-602 (2017).

39.	Lin S.H., Brown D.W. & Machiela M.J. LDtrait: An Online Tool for Identifying Published Phenotype Associations in Linkage Disequilibrium. *Cancer Res* **80**, 3443-3446 (2020).

40.	Verbanck M., Chen C.Y., Neale B. & Do R. Detection of widespread horizontal pleiotropy in causal relationships inferred from Mendelian randomization between complex traits and diseases. *Nat Genet* **50**, 693-698 (2018).

41.	Burgess S. & Thompson S.G. *Mendelian Randomization Methods for Causal Inference Using Genetic Variants*, (CRC Press. Taylor & Francis Group, 2021).

42.	Burgess S., Butterworth A. & Thompson S.G. Mendelian randomization analysis with multiple genetic variants using summarized data. *Genet Epidemiol* **37**, 658-65 (2013).

43.	Bowden J., Davey Smith G., Haycock P.C. & Burgess S. Consistent Estimation in Mendelian Randomization with Some Invalid Instruments Using a Weighted Median Estimator. *Genet Epidemiol* **40**, 304-14 (2016).

44.	Bowden J., Davey Smith G. & Burgess S. Mendelian randomization with invalid instruments: effect estimation and bias detection through Egger regression. *Int J Epidemiol* **44**, 512-25 (2015).

45.	Zhao Q., Wang J., Bowden J. & Small D. Statistical inference in two-sample summary-data Mendelian randomization using robust adjusted profile score. *Annals of Statistics* **48**, 1742-69 (2018).

46.	Hemani G., Tilling K. & Davey Smith G. Orienting the causal relationship between imprecisely measured traits using GWAS summary data. *PLoS Genet* **13**, e1007081 (2017).

47.	Hemani G., Zheng J., Elsworth B., Wade K.H., Haberland V., Baird D. *et al.* The MR-Base platform supports systematic causal inference across the human phenome. *Elife* **7**, 1-29 (2018).
